# Transposable elements cause the loss of self‐incompatibility in citrus

**DOI:** 10.1111/pbi.14250

**Published:** 2023-12-01

**Authors:** Jianbing Hu, Chenchen Liu, Zezhen Du, Furong Guo, Dan Song, Nan Wang, Zhuangmin Wei, Jingdong Jiang, Zonghong Cao, Chunmei Shi, Siqi Zhang, Chenqiao Zhu, Peng Chen, Robert M. Larkin, Zongcheng Lin, Qiang Xu, Junli Ye, Xiuxin Deng, Maurice Bosch, Vernonica E. Franklin‐Tong, Lijun Chai

**Affiliations:** ^1^ National Key Laboratory for Germplasm Innovation and Utilization of Horticultural Crops, College of Horticulture and Forestry Sciences Huazhong Agricultural University Wuhan P. R. China; ^2^ Hubei Hongshan Laboratory Wuhan P. R. China; ^3^ Guangxi Subtropical Crops Research Institute Nanning P. R. China; ^4^ Horticultural Institute, Hunan Academy of Agricultural Sciences Changsha China; ^5^ Institute of Biological, Environmental and Rural Sciences (IBERS) Aberystwyth University Aberystwyth UK; ^6^ School of Biosciences, College of Life and Environmental Sciences University of Birmingham Birmingham UK

**Keywords:** Self‐incompatibility, citrus, *S*‐RNase, *S*‐locus, MITE, evolution

## Abstract

Self‐incompatibility (SI) is a widespread prezygotic mechanism for flowering plants to avoid inbreeding depression and promote genetic diversity. Citrus has an *S*‐RNase‐based SI system, which was frequently lost during evolution. We previously identified a single nucleotide mutation in *S*_*m*_ *‐RNase,* which is responsible for the loss of SI in mandarin and its hybrids. However, little is known about other mechanisms responsible for conversion of SI to self‐compatibility (SC) and we identify a completely different mechanism widely utilized by citrus. Here, we found a 786‐bp miniature inverted‐repeat transposable element (MITE) insertion in the promoter region of the *FhiS*_*2*_ *‐RNase* in *Fortunella hindsii* Swingle (a model plant for citrus gene function), which does not contain the *S*_*m*_ *‐RNase* allele but are still SC. We demonstrate that this MITE plays a pivotal role in the loss of SI in citrus, providing evidence that this MITE insertion prevents expression of the *S‐RNase*; moreover, transgenic experiments show that deletion of this 786‐bp MITE insertion recovers the expression of *FhiS*_*2*_ *‐RNase* and restores SI. This study identifies the first evidence for a role for MITEs at the *S*‐locus affecting the SI phenotype. A family‐wide survey of the *S*‐locus revealed that MITE insertions occur frequently adjacent to *S‐RNase* alleles in different citrus genera, but only certain MITEs appear to be responsible for the loss of SI. Our study provides evidence that insertion of MITEs into a promoter region can alter a breeding strategy and suggests that this phenomenon may be broadly responsible for SC in species with the *S*‐RNase system.

## Introduction

Transposable elements (TEs) are the most variable components of the genome, which can be replicated and integrated into positions and mediate the genetic diversity and evolution in plants by affecting the expression levels of adjacent genes (Bennetzen and Wang, 2014; Lisch, 2013; Serrato‐Capuchina and Matute, 2018). Miniature inverted‐repeat transposable elements (MITEs) are considered to be deletion derivatives of DNA transposons, which have many copies in the genome and tend to be inserted in or near genes. They play an important role in the regulation of genes through various mechanisms (Chen *et al*., 2014; Feschotte *et al*., 2002; Lu *et al*., 2012). Previous studies have shown that TEs are crucial for plant reproduction (Wang *et al*., 2017; Xia *et al*., 2017), development (Xu *et al*., 2020), disease or stress resistance (Mao *et al*., 2015; Wu *et al*., 2022; Zhang *et al*., 2016) and domestication (Li *et al*., 2018; Lisch, 2013; Niu *et al*., 2019).

Over 40% of flowering plant species are self‐incompatible (SI) and thus, can produce fertile seeds after outcrossing but cannot produce zygotes after self‐pollination (Igic *et al*., 2008). SI systems can be divided into sporophytic self‐incompatibility (SSI) and gametophytic self‐incompatibility (GSI) by genetic mode of action. In SSI, the recognition specificity is determined by the genotype of the pollen‐producing plant (the sporophyte). In GSI, the *S*‐specificity is controlled by the genotype carried by the haploid gamete (i.e. the pollen) (Fujii *et al*., 2016; Takayama and Isogai, 2005). The *S*‐*RNase*‐based GSI is the most widely distributed system (Zhao *et al*., 2022). At present, six eudicot families (Solanaceae, Plantaginaceae, Rosaceae, Rubiaceae, Rutaceae and Cactaceae) have been demonstrated to use the *S*‐*RNase*‐based SI system (Du *et al*., 2021; Igic and Kohn, 2001; Liang *et al*., 2020; Ramanauskas and Igic, 2021; Sassa *et al*., 2007; Sijacic *et al*., 2004; Ushijima *et al*., 2003; Wang *et al*., 2003). This has an *S*‐locus composed of highly polymorphic, tightly linked pistil and pollen *S* determinants comprising the *S‐RNase* and multiple *S‐locus F‐box* (*SLF*) genes, respectively (Fujii *et al*., 2016; Takayama and Isogai, 2005; Wang *et al*., 2003). The *S‐RNase* ribonuclease activity is critical for self‐pollen rejection (Huang *et al*., 1994) and RNase activity in style tissue is positively correlated with ability to reject self‐pollen (Murfett *et al*., 1994); the *S*‐RNases enter pollen tubes indiscriminately and if not removed, inhibit pollen tube growth. Using a ‘non‐self’ recognition system, groups of SLFs recognize and detoxify several *S*‐RNases (Fujii *et al*., 2016).

It is generally acknowledged that SI, by preventing self‐fertilization, maintains genetic diversity (Goldberg *et al*., 2010). The shift from obligatory outcrossing to predominant self‐fertilization occurred repeatedly in convergent evolution (Barrett, 2002). Selfing is favoured for its inherent propagative and reproductive advantages in the absence of mates, pollinators or both. When the advantages of selfing outweigh the costs of inbreeding depression (e.g. reduced effective recombination rates, expansion of population subdivision, genetic bottlenecks, reductions in effective population size and selective interference between linkage sites), selfing evolves (Charlesworth and Willis, 2009; Charlesworth and Wright, 2001; Goldberg *et al*., 2010; Igic *et al*., 2008; Slotte *et al*., 2013). The evolution of selfing is usually accompanied by the loss of SI and evidence suggests that the early origin and ancestral status of the *S‐*locus and *S*‐*RNase*‐based SI in dicotyledonous plants indicates that SI was frequently lost during speciation (Fujii *et al*., 2016; Zhao *et al*., 2022).

Citrus is a tropical and subtropical perennial fruit tree from the Aurantioideae subfamily from the Rutaceae family that is widely cultivated worldwide. Many of the main cultivars are SI (Caruso *et al*., 2012; Chai *et al*., 2010; Gambetta *et al*., 2013; Honsho *et al*., 2009; Kakade *et al*., 2017; Yamashita and Tanimoto, 1985; Ye *et al*., 2009; Zhang *et al*., 2015), which substantially restricts yield and quality improvement efforts for some cultivars. The Aurantioideae consists of three major genera (*Citrus*, *Fortunella* and *Poncirus*) with distinct reproductive mechanisms (e.g. apomixis, outcrossing and self‐fertilization) (Shimada *et al*., 2018; Wang *et al*., 2017, 2022; Wu *et al*., 2018, 2021). We previously showed that citrus uses the *S‐RNase*‐based SI system and identified a dysfunctional mutant allele, *S*_*m*_ *‐RNase*, which comprises a single nucleotide mutation in the *S‐RNase*. Its fixation and propagation in *Citrus* genus led to the loss of SI (Liang *et al*., 2020). However, several genera that are SC, such as *Fortunella* and *Poncirus* genera do not contain the *S*_*m*_ *‐RNase* allele, implicating that there are other mechanisms responsible for the loss of SI in the Aurantioideae.

In this study, we document genetic variation at the SC‐associated loci in *F. hindsii* (Hongkong kumquat, also known as ‘Mini‐Citrus’) within a hybrid population and identify a 786‐bp MITE insertion in the promoter of *FhiS*_*2*_ *‐RNase* that results in the loss of SI in *F. hindsii*. Transgenic experiments show that deletion of the 786‐bp MITE insertion in the *FhiS*_*2*_ *‐RNase* promoter recovered the expression of *FhiS*_*2*_ *‐RNase* in the *F. hindsii* style and restored SI. We observed the frequent occurrence of MITE insertions near *S‐RNase* alleles in different intergeneric and interspecific of citrus and found that particular MITE insertions affect the expression of host genes, playing an important role in the loss of SI in citrus. In addition, MITE insertions have also been observed near *S‐RNases* alleles of other common GSI families, and MITE‐mediated SI loss is general in *S*‐RNase‐based SI system. Our analysis of the role of *S‐RNase*‐related MITE insertions in the evolution of selfing significantly broadens our understanding of another important mechanism contributing to SC in citrus. As *S‐RNase*s are widely distributed in several important plant families, our findings in citrus here are potentially relevant to the evolution of other *S*‐RNase‐based SI systems.

## Results

### *S*_*2*_ *‐RNase* is associated with the SC phenotype of *F. Hindsii*

**Figure 1:**
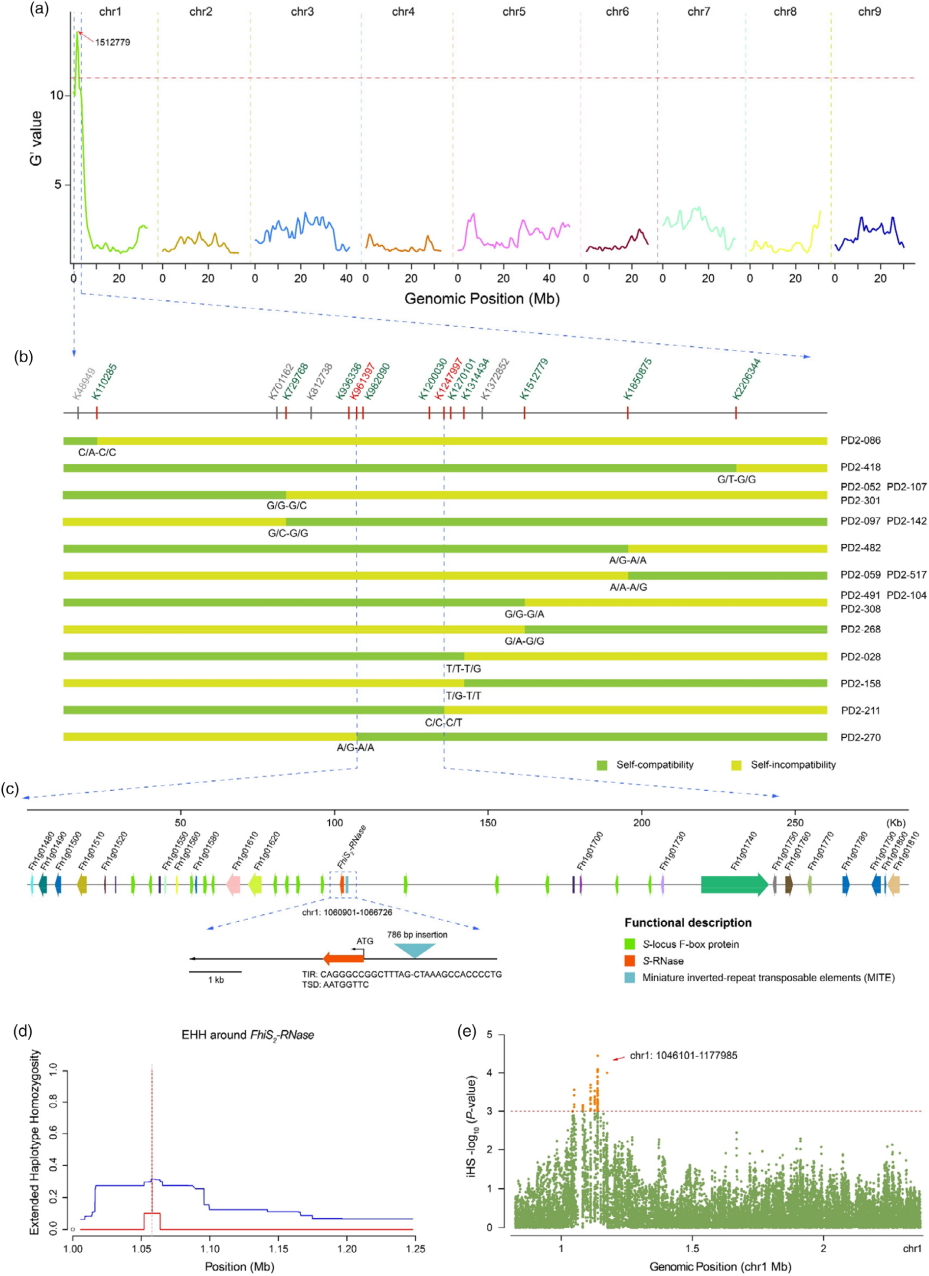
Genetic localization of key loci for self‐compatibility traits in a *F. hindsii* population derived from a PN02 × DB02 cross. (a) BSA‐Seq analysis of the F_1_ hybrid population (PN02 × DB02). G' values plotted against the single nucleotide polymorphism (SNP) positions on the *F. hindsii* (S3y‐45 v2.0) (Wang *et al*., 2022) genome. Major trait loci for self‐compatibility were identified using QTLseqr (Mansfeld and Grumet, 2018). The significance threshold (*P* ≤ 10^−3^) is indicated by the red horizontal line. The red arrow indicates a peak above the threshold value. (b) Fine mapping of the candidate gene using KASP genotyping. Eighteen recombinant offspring were classified into two groups according to the 16 pairs of KASP markers in the target region. Individuals with chromosome segment substitutions in the target region are shown. The SC haplotype is shown in pale green. The SI haplotype is shown in dark yellow. (c) Candidate genes in the target region containing two KASP markers (K961397 and K1247997). Recombinant analysis suggests the key gene of SC must be located in a 286.8‐kb interval that contains 35 genes. Coloured boxes represent different genes associated with the *S*_*2*_‐locus of *F. hindsii*. The red box represents an *S‐RNase* gene. The green boxes represent *SLF* genes. (d) Extended haplotype homozygosity (EHH) detected in the *FhiS*_*2*_ *‐RNase* genomic region. Red and blue line correspond to the long haplotype and alternative variants, respectively. (e) Signatures of selection at SC‐associated loci. Manhattan plot reflects the distribution of selection signatures of the initial interval (0.83 ~ 2.38 Mb, G' value = 11, *P* = 10^−3^, BSA‐Seq) with the integrated haplotype homozygosity score (iHS) statistic. The significance threshold (*P* ≤ 10^−3^) is indicated by the red horizontal line.

To explore the genetic basis underlying the conversion of SI to SC phenotype in *F. hindsii*, we performed a genetic analysis of F_1_ hybrids from a cross (PN02 × DB02) between the SI diploid line PN02 (*S*_*8*_ *S*_*19*_, *F. hindsii*) and the SC DB02 (*S*_*2*_ *S*_*29*_, *F. hindsii*). In 2019 and 2020, analysis of 186 F_1_ progeny established a ~ 1:1 segregation ratio of SC to SI (98 SC plants to 88 SI plants; χ^2^ = 0.54, *P* = 0.46; Figure S1; Table S1). These data indicate that SC in *F. hindsii* is caused by a single dominant gene or gametophytic factor (Table S2). Next, 32 SC and 32 SI F_1_ individuals were selected for a BSA (bulk segregation analysis) utilizing whole‐genome sequencing (WGS), which yielded a peak of SNPs linked to SC/SI on the anterior end of chromosome 1 (0.83 ~ 2.38 Mb, G' value = 11, *P* = 10^−3^, Figure 1a; Figure S2a). KASP (Kompetitive allele‐specific PCR) genotyping analysis was used to narrow the initial interval that was obtained from the BSA experiment. Using 12 pairs of markers, eighteen recombinant plants were identified from a total of 186 SC and SI F_1_ individuals. Based on two recombinant plants (PD02‐211 and PD02‐270) and their phenotypes, the key genetic loci controlling SC in *F. hindsii* were pinpointed to an interval between two markers, K961397 and K1247997 (286.6 kb, Figure 1b; Table S3). This candidate interval overlapped with the *S*‐locus that controls SI in citrus and contains 35 annotated genes, including 14 *F‐box* genes, one *S*_*2*_ *‐RNase* gene (named *FhiS*_*2*_ *‐RNase*, *F. hindsii*) and 20 conserved structural genes in the flanking region of the *S*‐locus (Figure 1c).

**Figure 2:**
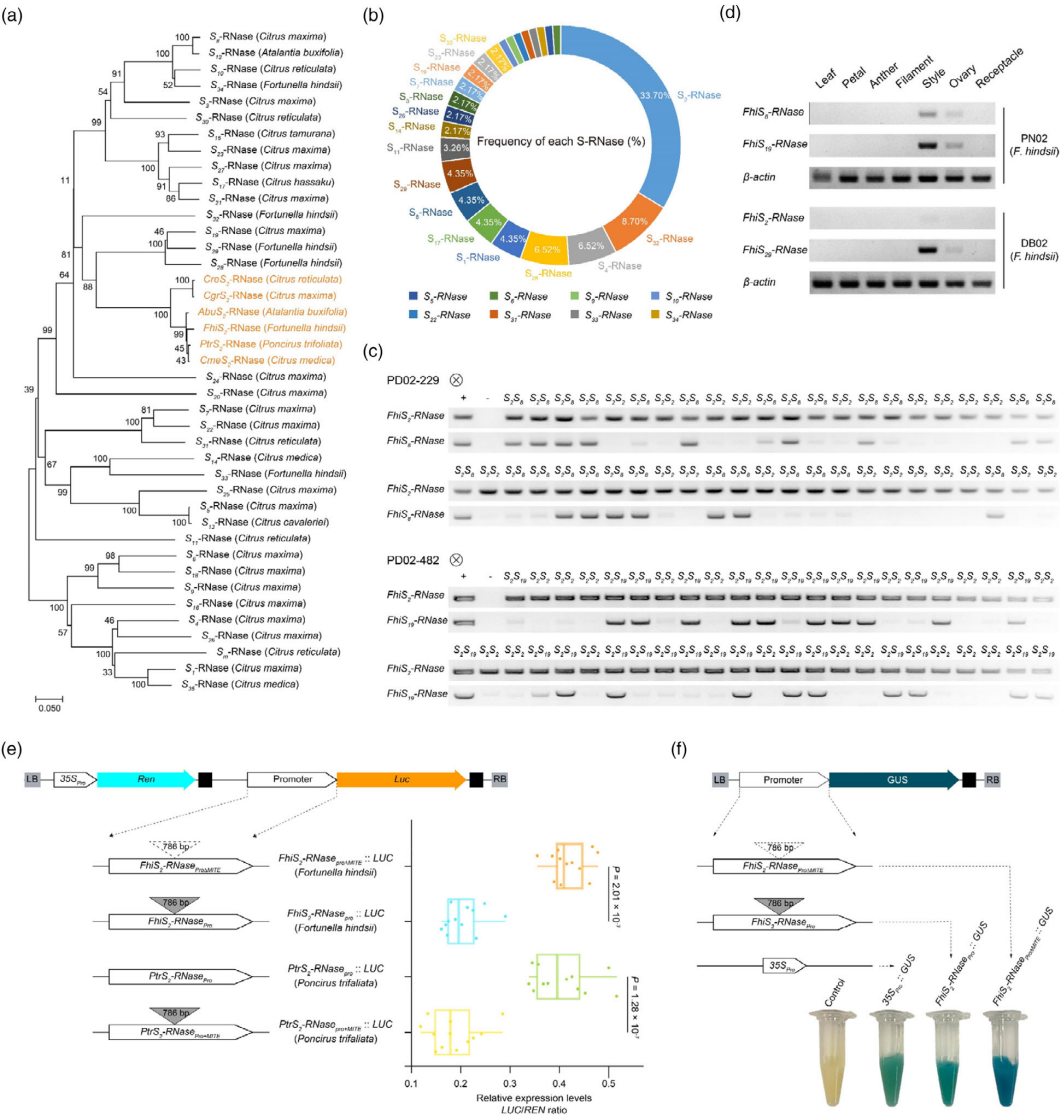
Influence of a MITE on the expression of the *FhiS*_*2*_ *‐RNase*. (a) Phylogeny of 6 *S*_*2*_ *‐RNase* alleles from different intergeneric and interspecific of citrus. The phylogenetic tree was derived from *PtrS*_*2*_ *‐RNase* (*Poncirus trifoliata*), *CmeS*_*2*_ *‐RNase* (*Citrus medica*), *FhiS*_*2*_ *‐RNase* (*Fortunella hindsii*), *AbuS*_*2*_ *‐RNase* (*Atlantia buxifolia*), *CgrS*_*2*_ *‐RNase* (*Citrus maxima*), *CreS*_*2*_ *‐RNase* (*Citrus reticulata*) (indicated in orange text) and 35 *S‐RNase* alleles from different intergeneric and interspecific of citrus. The neighbour‐joining phylogenetic tree was constructed using *S*‐RNase amino acid sequences and MEGA X (Kumar *et al*., 2018). (b) Frequency of the *S‐RNase* genotype in 46 *F. hindsii* accessions. The *S* haplotypes of these *F. hindsii* accessions were assigned based on PCR‐based genotyping of leaf DNA with 36 pairs of *S‐RNase*‐specific primers. These 46 accessions were collected from 5 provinces in China, and 24 *S‐RNase* alleles were identified (see Table S4). Most of the genotypes were present at a low frequency. The *S*_*2*_ *‐RNase* is present at the highest frequency of 33.7%. (c) Segregation of *S‐RNase*s with the *S*‐locus in the F_1_ progeny of self‐pollinated PD02‐229 and PD02‐482. Genotyping of the progeny from each pollination combination using PCR with *S*_*2*_‐, *S*_*8*_ *‐* and *S*_*19*_ *‐RNase* specific primers showed that PD02‐229 and PD02‐482 carried the same *S*_*2*_‐*RNase* allele. Pairs of amplified *S‐RNase* sequences in three different combinations (*S*_*2*_ *S*_*8*_, *S*_*2*_ *S*_*19*_ and *S*_*2*_ *S*_*2*_) were amplified from 72 progeny from each self‐pollination combination. (d) Semi‐quantitative PCR was used to detect the expression of *S‐RNase* alleles in different tissues from DB02 and PN02. The *S‐RNase* allele was expressed only in the ovary and style tissues of pistils. The expression levels of the *FhiS*_*2*_ *‐RNase* allele in the style tissues of DB02 were much lower relative to the other three alleles. The expression levels of these three alleles (*FhiS*_*8*_ *‐RNase*, *FhiS*_*19*_ *‐RNase* and *FhiS*_*29*_ *‐RNase*) were similar in the style tissue of *F. hindsii*. (e) Activities of promoter fragments from *S*_*2*_ *‐RNase* alleles in *F. hindsii* and *P. trifoliata*. The structure of the pGreenII 0800‐LUC vector (top) and constructs with different promoter fragments from the *S*_*2*_ *‐RNase* allele (left) are shown. The expression of the *firefly luciferase* (*LUC*) reporter gene was driven by different target promoter fragments. The expression of *Renilla Luciferase* (*Ren*) was used as an internal control. The expression levels of *LUC* relative to *REN* are shown with a boxplot (right). The horizontal lines indicate statistically significant differences (*n* = 12 biological replicates, two‐tailed Student's *t*‐tests). (f) Activity of *FhiS*_*2*_ *‐RNase* promoter fragments in *F. hindsii*. Promoter fragments from the *S*_*2*_ *‐RNase* allele were fused to the *GUS* (*β*‐glucuronidase) reporter gene and expressed in the callus of DB02 using an *Agrobacterium*‐based transient transformation method. A schematic diagram of the pK7WFS7 vector (above), diagram of the different promoter fragments from the *FhiS*_*2*_ *‐RNase* allele (left) and staining of callus (below) are shown. p*35S*::*GUS* was used as the control.

SC‐associated loci are usually under strong selection in the population (Guo *et al*., 2011). Focusing on this candidate region, to search for genomic regions with signs of recent positive selection, we calculated the extended haplotype homozygosity (EHH) of SC *F. hindsii* accessions (Table S4), which measures decay of haplotypes that carry a specified core allele as a function of distance and obtained the integrated haplotype score (iHS) statistic for each SNP. This identified a strong positive selection signal in the *FhiS*_*2*_ *‐RNase* genomic region, which contained 75 significant SNPs (*P* = 10^−3^) and was associated with 13 of 35 annotated genes mentioned above (Figure 1d,e; Figure S2b; Table S5). The SC phenotype was tightly linked to the *S*_*2*_ *‐RNase* allele in the F_1_ progeny of the hybrid population (Table S1). Sequence variations including several synonymous and non‐synonymous mutations in the 3′ region of the *FhiS*_*2*_ *‐RNase* allele from *F. hindsii* were predicted to result in a *FhiS*_*2*_‐RNase protein with 12 more amino acid residues relative to the other five alleles that encode *S*_*2*_‐RNases (*PtrS*_*2*_‐RNase, *CmeS*_*2*_‐RNase, *AbuS*_*2*_‐RNase, *CgrS*_*2*_‐RNase and *CreS*_*2*_‐RNase), although the conserved domain (C1‐C5) (Liang *et al*., 2020) was unaltered (Figure 2a; Figure S3).

Frequency‐dependent selection is a key feature of SI, and it determines the evolutionary dynamics of alleles at the *S*‐locus (Castric and Vekemans, 2004; Wright, 1939). Genotypes and SI phenotypes were characterized in the 46 *F. hindsii* accessions, collected from 5 provinces in China (Table S4). The *S*_*2*_ *‐RNase* genotype was most abundant (30/46) and was found in up to 33.7% of accessions (Figure 2b). Strikingly, all the accessions containing the *S*_*2*_ *‐RNase* genotype were SC (Table S4). To detect the positive selection signatures between SC and SI *F. hindsii*, Cross‐Population Extended Haplotype Homozygosity (XP‐EHH) tests were performed. Four peaks were detected and 34 putatively advantageous positively selected genes were obtained (Figure S2c; Table S6), of which 17 genes overlapped with the KASP genotyping results, indicating that these genes, including *FhiS*_*2*_ *‐RNase*, were strongly selected in the SC *F. hindsii* lines.

To further assess if the *FhiS*_*2*_ *‐RNase* was the candidate gene responsible for the change in phenotype from SI to SC in *F. hindsii*, the segregation ratios of the *S* haplotypes in the selfed progeny (S_1_) were analysed using chi‐square tests. The *S* haplotypes of the progeny from the self‐pollinated PD02‐229 (*S*_*2*_ *S*_*8*_), rather than segregating into a 1:2:1 ratio, segregated into two classes (*S*_*2*_ *S*_*2*_:*S*_*2*_ *S*_*8*_) at a ratio of 62:63. The *S*_*8*_ *S*_*8*_ genotype, which was an expected outcome of this self‐cross, was absent from the S_1_ progeny (Table 1, Figure 2c). This is consistent with an SC mutation linked to the *S*_*2*_‐locus (1:1, χ^2^ = 0.01, *P* = 0.93). Furthermore, 116 individuals from the S_1_ progeny derived from the self‐pollinated PD02‐482 (*S*_*2*_ *S*_*19*_) also segregated as 1:1 with *S*_*2*_ *S*_*2*_ (55 plants) and *S*_*2*_ *S*_*19*_ (61 plants) genotypes (1:1, χ^2^ = 0.31, *P* = 0.58), with the expected *S*_*19*_ *S*_*19*_ genotype being absent from the S_1_ progeny (Table 1; Figure 2c). These data show that only pollen carrying the *S*_*2*_ *‐RNase* allele, but not the *S*_*8*_ *‐RNase* or the *S*_*19*_ *‐RNase*, are able to transmit to the S_1_ progeny (Figure 2c), further supporting the hypothesis that the *FhiS*_*2*_ *‐RNase* is associated with the disruption of SI in *F. hindsii*.

**Table 1:** Segregation of *S‐RNases* in F_1_ progeny in a gametophytic manner

Cross	No. of progeny	Possible genotypes^a^	Observed ratio^b^	Expected ratio^c^	χ^2^ value	*P*‐value
PD02‐229 (*S*_*2*_ *S*_*8*_) × PD02‐229 (*S*_*2*_ *S*_*8*_)	125	*S* _ *2* _ *S* _ *2* _: *S* _ *2* _ *S* _ *8* _:*S*_*8*_ *S*_*8*_	62:63:0	1:1:0	0.01	0.93
				1:2:1	61.51	4.39 × 10^−14^
PD02‐482 (*S*_*2*_ *S*_*19*_) × PD02‐482 (*S*_*2*_ *S*_*19*_)	116	*S* _ *2* _ *S* _ *2* _: *S* _ *2* _ *S* _ *19* _:*S*_*19*_ *S*_*19*_	55:61:0	1:1:0	0.31	0.58
				1:2:1	52.47	4.05 × 10^−12^
PN01 (*S*_*7*_ *S*_*28*_) × PN03 (*S*_*4*_ *S*_*7*_)	43	*S* _ *4* _ *S* _ *7* _: *S* _ *4* _ *S* _ *28* _:*S*_*7*_ *S*_*7*_:*S*_*7*_ *S*_*28*_	23:20:0:0	1:1:0:0	0.21	0.65
				1:1:1:1	43.42	2.01 × 10^−9^
PN03 (*S*_*4*_ *S*_*7*_) × PN01 (*S*_*7*_ *S*_*28*_)	53	*S*_*4*_ *S*_*7*_:*S*_*7*_ *S*_*7*_: *S* _ *4* _ *S* _ *28* _: *S* _ *7* _ *S* _ *28* _	0:0:24:29	0:0:1:1	0.47	0.49
				1:1:1:1	53.94	1.15 × 10^−11^

Segregation analysis of *S* haplotypes in S_1_ progeny from *F. hindsii* accessions from self‐pollination and cross‐pollination experiments analysed using PCR (Figure 2C).

^a^ The observed genotypes are underlined.

^b^ The *S*‐genotype ratios observed in all progeny.

^c^ The upper segregation ratio is the ratio expected from a single genotype mutation model from the GSI system. The lower segregation ratio is the ratio expected from simple Mendelian inheritance.

### A MITE suppresses the transcription of the *S*_*2*_ *‐RNase*

Semi‐quantitative real‐time RT‐PCR (SqRT‐PCR) revealed that the expression of the *FhiS*_*2*_ *‐RNase* in pistil tissue was significantly lower than the *FhiS*_*29*_ *‐RNase* expression in SC DB02 (*S*_*2*_ *S*_*29*_, *F. hindsii*), as well as the *FhiS*_*8*_ *‐RNase* and *FhiS*_*19*_ *‐RNase* expression in SI PN02 (*S*_*8*_ *S*_*19*_, *F.hindsii*) (Figure 2d; Figure S4), suggesting that the down‐regulated expression of the *FhiS*_*2*_ *‐RNase* might be responsible for the SC phenotype. However, we found that the *FhiS*_*2*_ *‐RNase* transcript was intact, with no mutations in it. A 786‐bp insertion located 1071 bp upstream of the start codon of the *FhiS*_*2*_ *‐RNase*, was identified when comparisons between sequences of *S*_*2*_ *‐locus* of DB02 (SC, *S*_*2*_ *S*_*29*_, *F. hindsii*) and ZK8 (SC, *S*_*2*_ *S*_*31*_, *Poncirus trifoliata*, common wild trifoliata orange, a widely used citrus rootstock) were made (Figure S5). This comparison was made as, due to the significant differences in the non‐coding region of *S*_*2*_ *‐RNase* in different intergeneric of citrus, only ZK8 (*P. trifoliata*, *S*_*2*_ *S*_*31*_) and DB02 (*F. hindsii*, *S*_*2*_ *S*_*29*_) share high homology in this region. Using the P‐MITE database (Chen *et al*., 2014) and RepeatMasker, we determined that the 786‐bp insertion sequence was a MITE transposon that belongs to the *hAT* (*hobo, Ac and Tam3*) family. To establish if the MITE insertion in the *FhiS*_*2*_ *‐RNase* promoter region contributed to changes in the expression of this allele, we generated various constructs by inserting different combinations of promoter fragments from *F. hindsii* into the binary vectors pGreenII 0800‐*LUC* and pKGWFS7. We introduced these constructs into DB02 callus and examined the expression of the *luciferase* reporter gene (*LUC*). The MITE deletion (construct *FhiS*_*2*_ *‐RNase*_*proΔMITE*_::*LUC*) significantly increased the promoter activity of *FhiS*_*2*_ *‐RNase* (Figure 2e, *P* = 2.01 × 10^−7^), this was consistent with the results from the GUS histochemical staining (Figure 2f). In addition, when the MITE insertion (construct *PtrS*_*2*_ *‐RNase*_*pro+MITE*_::*LUC*) was introduced into the *S*_*2*_ *‐RNase* promoter of *P. trifoliata* at same position as the *FhiS*_*2*_ *‐RNase* promoter, it significantly decreased the promoter activity of the *PtrS*_*2*_ *‐RNase* (*P* = 1.28 × 10^−7^, Figure 2e). These data demonstrate that the MITE insertion can modulate expression levels of *FhiS*_*2*_ *‐RNase* in *F. hindsii*, suggesting that this 786‐bp MITE could be responsible for the SC phenotype.

### Evidence for CHH methylation clustered around the MITE transposon

**Figure 3:**
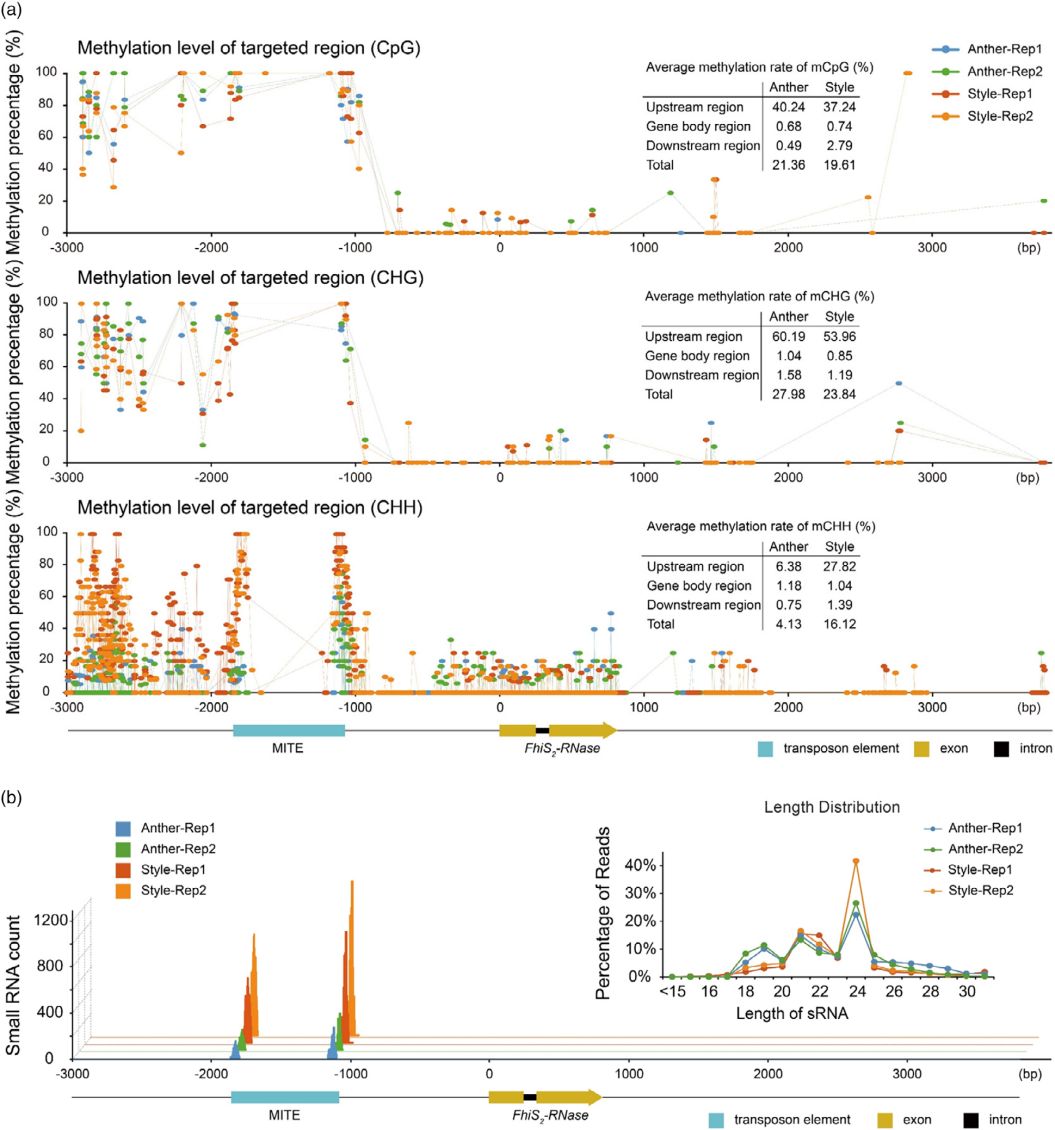
Influence of methylation may be involved in the expression of the *FhiS*_*2*_ *‐RNase*. (a) Methylation levels of the *FhiS*_*2*_ *‐RNase* allele in the leaf, anther and style of *F. hindsii*. Methylation levels in the CpG (top row), CHG (middle row) and CHH (bottom row) contexts were quantified in 3‐kb 5′‐flanking regions, exons, introns and 3‐kb 3′‐flanking regions. The percentage of total mCs is the number of mCs/total number of Cs. (b) Numbers of small RNAs from anthers and styles that map to the 3‐kb 5′‐flanking region of the *FhiS*_*2*_ *‐RNase* allele in DB02. Most small RNAs map to the MITE region. The linear graph in the upper right corner shows the length distribution of small RNAs in anther and style tissue of DB02.

The results of *in vitro* experiments suggested that the effect of 786‐bp insertion on the expression levels of reporter genes (*LUC* and *GUS*) were not as completely suppressed as expected *in vivo* (Figure 2d–f; Figure S4). As cytosine methylation in plants is an epigenetic marker critical for transposon silencing, we investigated if this might be responsible for the attenuated expression of the *FhiS*_*2*_ *‐RNase* in the *F. hindsii*. Whole‐genome bisulphite sequencing (WGBS) profiling of DNA methylation in the anther and style tissues from SC DB02 (*S*_*2*_ *S*_*29*_), revealed that the CHH methylation levels in the style tissue of the *FhiS*_*2*_ *‐RNase* region were significantly higher than in the anther tissue, with methylation rates of 16.12% and 4.13%, respectively, while the depth of coverage at cytosines and the average CpG, CHG and CHH methylation levels across the whole genome were similar in each tissue (Tables S7 and S8). Moreover, CHH methylation was mainly clustered around the MITE transposon that was inserted in the *FhiS*_*2*_ *‐RNase* promoter region (Figure 3a). In contrast, CpG, CHG and CHH methylation levels in the other *S* allele (*FhiS*_*29*_ *‐RNase*) region of the SC DB02 were not significantly different in anther and style tissues (Figure S6a). These data suggest that CHH methylation may contribute to the attenuated expression of the *FhiS*_*2*_ *‐RNase* in the style of *F. hindsii*.

Studies have shown that TE‐related methylation was usually associated with small RNAs (Borges and Martienssen, 2015; Zhang *et al*., 2018). Using small RNA sequencing, we found that a set of partially overlapping 24‐nt siRNAs (small interfering RNAs) were generated from the flanking regions of the MITE in the upstream promoter of *FhiS*_*2*_ *‐RNase* and that the partially overlapping siRNA peaks at both ends of the MITE were significantly higher in styles relative to levels in anther tissue (Figure 3b; Tables S9 and S10). In contrast, no siRNA peaks were detected in the *FhiS*_*29*_ *‐RNase* region (Figure S6b). This is consistent with the CHH methylation of the *FhiS*_*2*_ *‐RNase* promoter region in the style tissue of SC DB02 and with the idea that the 24‐nt siRNAs generated from the MITE regulates *FhiS*_*2*_ *‐RNase* expression by mediating the methylation of the *FhiS*_*2*_ *‐RNase* promoter region.

### Recovered expression of *S*_*2*_ *‐RNase* restores the SI phenotype of *F. Hindsii*

**Figure 4:**
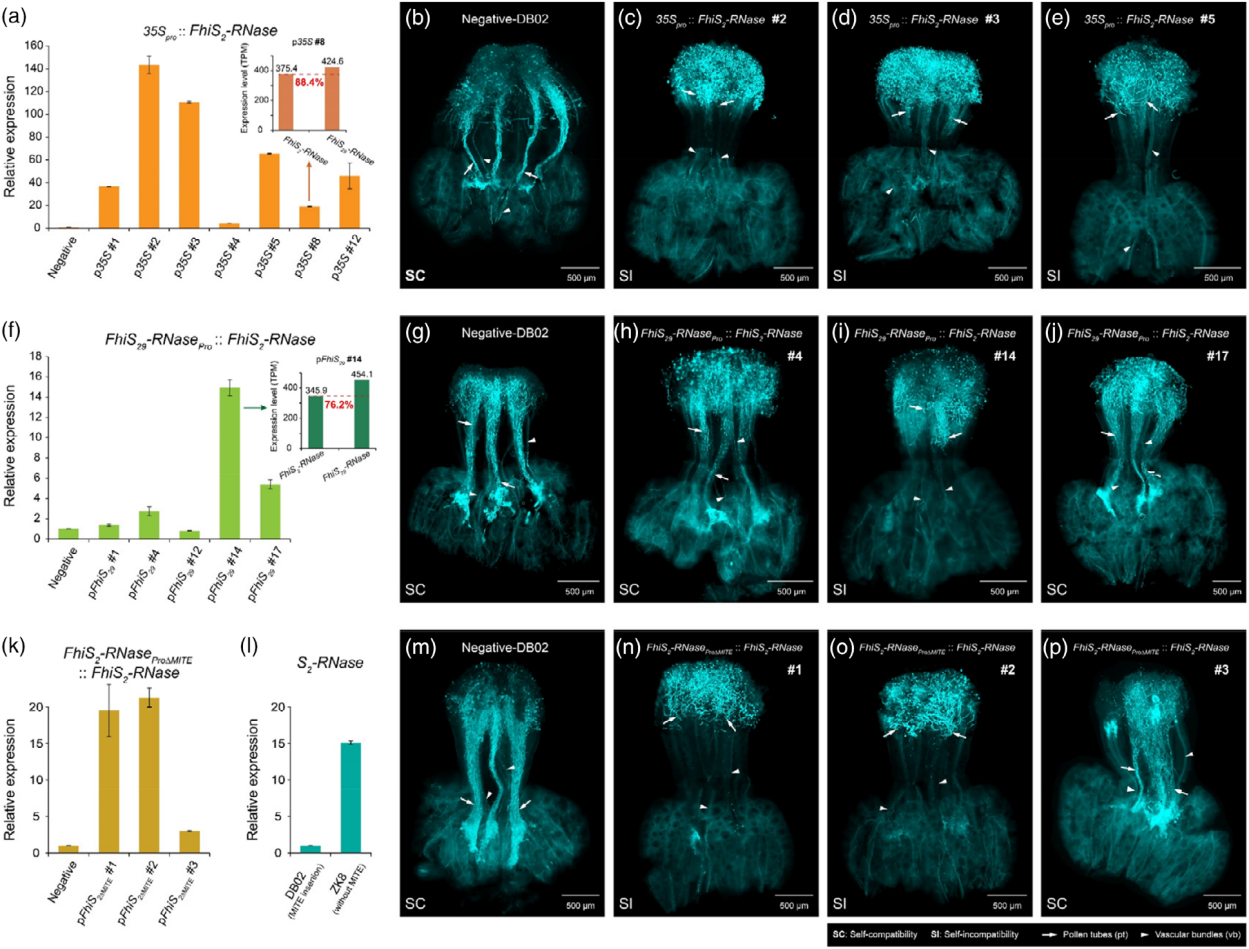
Recovered expression of *FhiS*_*2*_ *‐RNase* restores the SI phenotype of *F. hindsii*. (a) Comparison of the relative expression levels of the *FhiS*_*2*_ *‐RNase* allele in the styles of a negative transgenic line and 7 overexpression lines (*n* = 3 biological replicates). The small bar chart (upper right) shows the transcript levels of *FhiS*_*2*_ *‐RNase* and *FhiS*_*29*_ *‐RNase* alleles in transgenic line (*35S*_*pro*_::*FhiS*_*2*_ *‐RNase* #8). (b–e) Representative aniline blue staining of self‐pollinated styles from *35S*_*pro*_::*FhiS*_*2*_ *‐RNase*‐DB02 transgenic plants (*n* = 12). Negative indicates plants from the transformation that lost the transgene because of segregation. (f) Relative expression levels of the *FhiS*_*2*_ *‐RNase* allele in the styles of negative controls and 5 transgenic lines (*FhiS*_*29*_ *‐RNase*_*pro*_::*FhiS*_*2*_ *‐RNase*). The small bar chart (upper left) shows the transcript levels of *FhiS*_*2*_ *‐RNase* and *FhiS*_*29*_ *‐RNase* alleles in transgenic line (*FhiS*_*29*_ *‐RNase*_*pro*_::*FhiS*_*2*_ *‐RNase* #14). TPM values were calculated with the reads mapped to the DB02 assembled reference genome. The percentage below the dotted red line indicates the ratio of the *FhiS*_*2*_ *‐RNase* transcription level in the transgenic line to the endogenous other *S* allele (*FhiS*_*29*_ *‐RNase*) expression level. (g–j) Representative aniline blue staining images of pollen tubes in pistils from *FhiS*_*29*_ *‐RNase*_*pro*_::*FhiS*_*2*_ *‐RNase*‐DB02 transgenic plants (*n* = 17). (k) Relative expression levels of the *FhiS*_*2*_ *‐RNase* allele in the styles of negative controls and 3 transgenic lines (*FhiS*_*2*_ *‐RNase*_*proΔMITE*_::*FhiS*_*2*_ *‐RNase*). Data are presented as means ± SE. (*n* = 3 biological replicates). (l) Comparison of the relative expression levels of the *S*_*2*_ *‐RNase* allele in the styles of DB02 (*F. hindsii*, *S*_*2*_ *S*_*29*_; the promoter of *FhiS*_*2*_ *‐RNase* contains a 786‐bp MITE insertion) and ZK8 (*P. trifoliata*, *S*_*2*_ *S*_*31*_; Compared with *FhiS*_*2*_ *‐RNase*, the promoter of *PtrS*_*2*_ *‐RNase* does not contain the MITE insertion, Figure S5). Data are presented as means ± SE. (*n* = 3 biological replicates). (m–p) Representative aniline blue staining images of pollen tubes in pistils from *FhiS*_*2*_ *‐RNase*_*proΔMITE*_::*FhiS*_*2*_ *‐RNase*‐DB02 transgenic plants (*n* = 3). Five images were acquired for each pollination combination. Representative fluorescence images from aniline blue stained pistils are shown that were acquired at 1 day before anthesis and at 2 days after pollination. Scale bars = 500 μm. Pollen tubes (pt) are indicated with arrows. Vascular bundles (vb) are indicated with arrowheads.

As our data suggested that lower expression levels of *FhiS*_*2*_ *‐RNase* in style tissue might be responsible for the loss of SI in *F. hindsii*, we wished to ascertain what might be responsible for this. We, therefore, generated transgenic SC DB02 (*S*_*2*_ *S*_*29*_) *F. hindsii* plants with increased expression of *FhiS*_*2*_ *‐RNase* using *35S* promoter (*35S*_*pro*_::*FhiS*_*2*_ *‐RNase*‐DB02), the native promoter of *FhiS*_*29*_ *‐RNase* (*FhiS*_*29*_ *‐RNase*_*pro*_::*FhiS*_*2*_ *‐RNase*‐DB02) and its own promoter of *FhiS*_*2*_ *‐RNase* without 786‐bp MITE insertion (*FhiS*_*2*_ *‐RNase*_*proΔMITE*_::*FhiS*_*2*_ *‐RNase*‐DB02) (Figure S7a–c). The relative expression levels of the *FhiS*_*2*_ *‐RNase* were significantly elevated in transgenic lines relative to the empty vector control plants. Twelve stably transformed and flowering lines (seven *35S*_*pro*_::*FhiS*_*2*_ *‐RNase*‐DB02 and five *FhiS*_*29*_ *‐RNase*_*pro*_::*FhiS*_*2*_ *‐RNase*‐DB02 lines) were selected for a follow‐up study (Figure 4a,f; Figure S7a).

The expression levels of *FhiS*_*2*_ *‐RNase* in these SC *F. hindsii* transgenic lines ranged from 0.8‐fold to 143.3‐fold increase (Figure 4a,f; Figure S7d). In the line *FhiS*_*29*_ *‐RNase*_*pro*_::*FhiS*_*2*_ *‐RNase*‐DB02 #14, where the expression of *FhiS*_*2*_ *‐RNase* was increased by more than 14.9‐fold (which corresponded to ~76% of the expression level of the other endogenous *S* allele, *FhiS*_*29*_ *‐RNase*; Figure 4f), the SI phenotype was restored (Figure 4b–e,i; Figure S7e). The relative expression levels of the other five lines (*35S*_*pro*_::*FhiS*_*2*_ *‐RNase*‐DB02 #4, *FhiS*_*29*_ *‐RNase*_*pro*_::*FhiS*_*2*_ *‐RNase*‐DB02 #1, #4, #12, #17) were elevated by 5.5‐fold or less (which corresponded to ~25% of the expression level of the other endogenous *S* allele, *FhiS*_*29*_ *‐RNase*; Figure 4a,f), and aniline blue staining of pollinations showed that these lines and the empty vector control plants all maintained the SC phenotype (Figure 4g,h,j; Figure S7e). These data suggests that when *FhiS*_*2*_ *‐RNase* is expressed beyond a certain threshold level (~75% of *S‐RNase* expression in SI plants) in SC *F. hindsii*, the SI phenotype can be restored. In contrast, when the expression levels of the *FhiS*_*2*_ *‐RNase* allele in the style tissue of the transgenic lines were similar or only increased by ~5‐fold to the empty vector control plants, the transgenic lines remained SC (Figure 4g,h,j; Figure S7e). These data demonstrate that the loss of SI in *F. hindsii* is due to the low expression level of the *FhiS*_*2*_ *‐RNase*. When the *FhiS*_*2*_ *‐RNase* is expressed above a threshold level, it acts as a functional female *S*‐determinant that is responsible for the specific inhibition of self‐pollen tubes. These data provide evidence that *FhiS*_*2*_ *‐RNase* performs the same function as *S*_*2*_ *‐RNases* in the other intergeneric of citrus. Moreover, they demonstrate that highly expressed *FhiS*_*2*_ *‐RNase* in stylar tissues can restore the SI phenotype. As overexpression of the *S*_*2*_ *‐RNase* was sufficient to restore the SI phenotype in self‐fertile *F. hindsii*, this suggests that all the components required for SI are retained except for the *S*_*2*_ *‐RNase* allele.

### Transgenic lines demonstrate that removal of the 786‐bp MITE insertion restores the SI phenotype of *F. Hindsii*

Although making transgenic lines in woody tree species is highly time‐consuming, to confirm whether the MITE insertion in the promoter of *FhiS*_*2*_ *‐RNase* was responsible for its low expression level, we constructed complementary transgenic lines with 786‐bp MITE insertion removed (*FhiS*_*2*_ *‐RNase*_*proΔMITE*_::*FhiS*_*2*_ *‐RNase*‐DB02). Among the obtained 3 flowering transgenic lines, the relative expression of 2 lines was increased about 20‐fold (Figure 4k; Figure S7a), and the results of aniline blue staining showed that their styles could reject the growth of self‐pollen tubes, indicating that the SI phenotype had been restored compared with the control (Figure 4m–o). While aniline blue staining of pollinations showed that the *FhiS*_*2*_ *‐RNase*_*proΔMITE*_::*FhiS*_*2*_ *‐RNase*‐DB02 #3 line still maintained the SC phenotype (Figure 4p), and its relative expression level was just elevated by ~3‐fold (Figure 4k). Furthermore, we also found that the expression of *S*_*2*_ *‐RNase* (*PtrS*_*2*_ *‐RNase*) in ZK8 (*P. trifoliata*, *S*_*2*_ *S*_*31*_, common wild trifoliata orange) style was significantly higher than that of the DB02 (*F. hindsii*, *FhiS*_*2*_ *‐RNase*), and its promoter lacked 786‐bp MITE insertion compared to *FhiS*_*2*_ *‐RNase* (Figure 4l; Figures S4a,c, and S5). These results indicated that the 786‐bp MITE insertion inhibited the expression of *FhiS*_*2*_ *‐RNase* and was responsible for the loss of SI in *F. hindsii*.

### Diverse SC‐linked structural variations do not directly affect loss of SI in *F. Hindsii*

It has been reported that SVs are involved in the loss of SI and play an important role in the evolution of *S*‐locus (Li *et al*., 2019; Okada *et al*., 2008); in *Arabidopsis* SC haplotypes are mainly generated by recombination and SVs (Goubet *et al*., 2012; Tsuchimatsu *et al*., 2017). Due to the high polymorphic nature of the *S*‐locus, the identification of SC‐linked SVs is challenging. To further assess the structural differences and to identify SC‐specific features of *S* haplotype in *F. hindsii*, we sequenced and assembled the genomes of SC DB02 and SI PN02. The genomes were based on single molecule real‐time (SMRT) DNA sequencing and *de novo* assembled with Canu (Koren *et al*., 2017), which produces two partially phased diploid genomes (a DB02gv1 contig and a PN02gv1 contig; Table S11). Four complete *S*‐locus sequences (Fhi‐*S*_*2*_‐locus, Fhi‐*S*_*8*_‐locus, Fhi‐*S*_*19*_‐locus and Fhi‐*S*_*29*_‐locus) were obtained using the 200‐bp sequences of two non‐recombinant KASP markers (K961397 and K1247997) as the boundary (Table S3).

**Figure 5:**
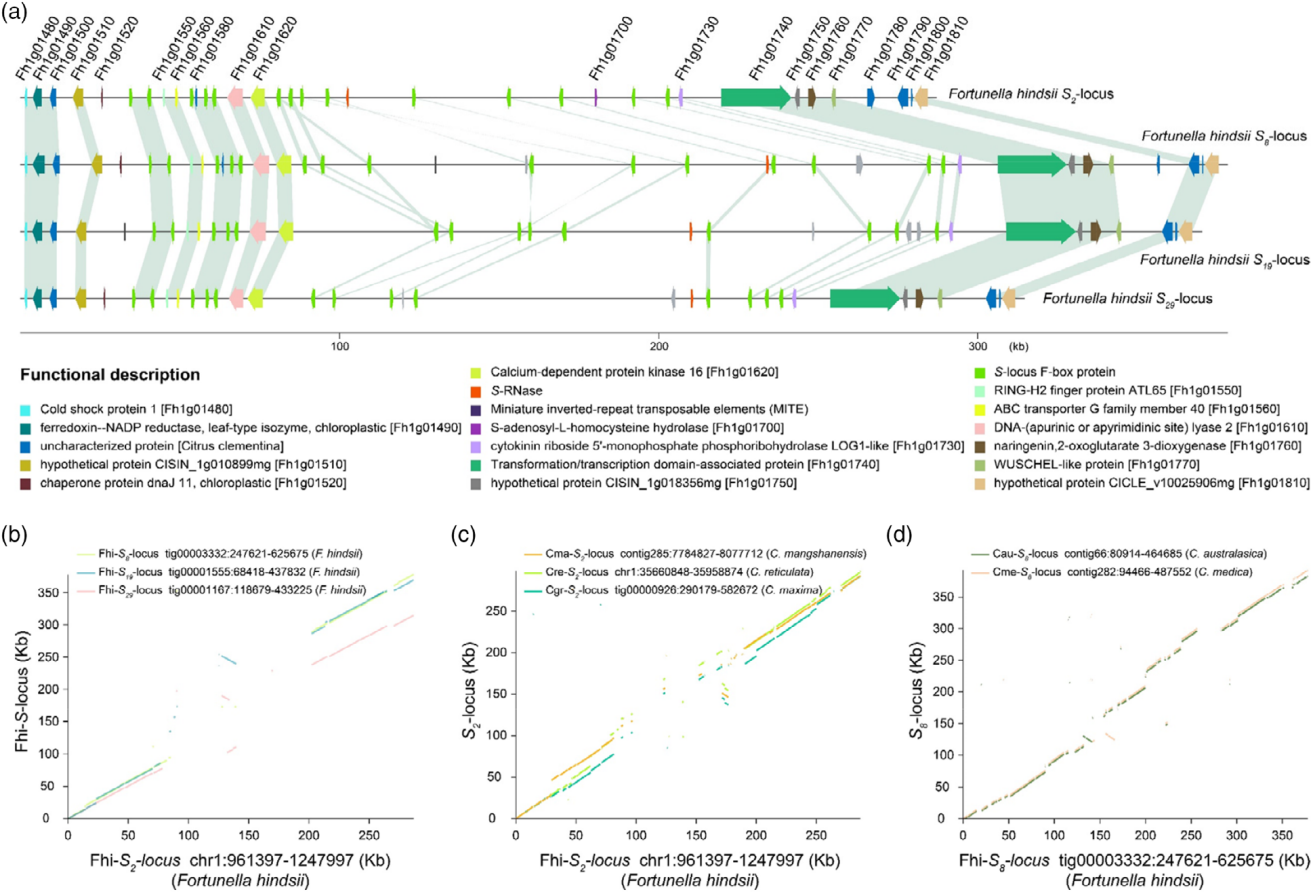
Structural variant analysis of *S*‐locus in DB02 and PN02. (a) Schematic diagram for the gene annotation and collinearity analysis of 4 *S*‐locus in the genomes of DB02 and PN02. The blue and grey lines represent the syntenic sequences among the genes at the four *S*‐locus of *F. hindsii*. The coloured boxes represent different genes associated with the Fhi‐*S*_*2*_‐locus, Fhi‐*S*_*8*_‐locus, Fhi‐*S*_*19*_‐locus and Fhi‐*S*_*29*_‐locus. The red boxes represent *S‐RNase* alleles. The green boxes represent the *SLF* alleles. (b) Whole‐sequence alignments of the *S*‐locus region in DB02 and PN02. The 200‐bp sequences of two non‐recombinant KASP markers (K961397 and K1247997) were used as the boundaries of the *S*‐locus. Sequences of the Fhi‐*S*_*2*_‐locus, Fhi‐*S*_*8*_‐locus, Fhi‐*S*_*19*_‐locus and Fhi‐*S*_*29*_‐locus were obtained from the DB02 and PN02 genomes (DB02gv1 and PN02gv1). (c) Whole‐sequence alignment analysis of the *S*_*2*_‐locus region in different intergeneric and interspecific of citrus. The sequences of the Cma‐*S*_*2*_‐locus, Cre‐*S*_*2*_‐locus and Cgr‐*S*_*2*_‐locus were derived from the genomes of ‘mangshanyeju’ (*Citrus mangshanensis*), ‘Ponkan’ (*Citrus reticulata* Blanco *cv*. Ponkan) and pummelo (*Citrus maxima*), respectively. The Fhi‐*S*_*2*_‐locus sequence was used as reference. (d) Whole‐sequence synteny analysis of the *S*_*8*_‐locus region for different interspecific of citrus. Cau‐*S*_*8*_‐locus and Cme‐*S*_*8*_‐locus were derived from the genomes of *C. australasica* and *C. medica*, respectively. The Fhi‐*S*_*8*_‐locus sequences were used as references. Detailed information on *S*‐locus in different intergeneric and interspecific of citrus is provided in Table S14.

These four *S*‐locus were annotated based on available transcriptome data (Figure 5a) and the SI haplotypes were aligned to the *F. hindsii* (S3y‐45 v2.0) (Wang *et al*., 2022) Fhi‐*S*_*2*_‐locus to assess structural differences and to identify SC‐specific features. There were differences in length among the SI and SC haplotypes in *F. hindsii* (Figure 5b). The *S*‐locus ranged in size from ~286.8 to ~378.1 kb, but the Fhi‐*S*_*2*_‐locus were significantly shorter relative to other SI haplotypes (Figure 5a). These length differences appear to be related to SC‐linked structural variations. The SI haplotypes share 15 small insertions and 6 small deletions relative to the Fhi‐*S*_*2*_‐locus, encompassing a total length of 30.2 kb. These SVs were located mainly in the flanking regions of the *S*‐locus. In addition, there were 7 translocations and 8 inversions in the Fhi‐*S*_*2*_‐locus relative to the three other SI haplotypes, including 2 large inversions located between two *SLFs* alleles (*SLF4* and *SLF5*; Figure 5b). Although the ~125‐kb middle region of different *S*‐locus in *F. hindsii* was highly polymorphic, the sequences flanking both ends of the *S*‐locus were conserved and contained a highly collinear group of genes. Most of the polymorphic regions were composed of repeat sequences and TEs, including MITEs, long terminal‐repeat (LTR) retrotransposons, gypsy elements and copia elements (Figure 5a,b; Table S12). It is noteworthy that, compared to the SC Fhi‐*S*_*2*_‐locus of *F. hindsii*, the other three SI *S*_*2*_ haplotypes (Cma‐*S*_*2*_‐locus, Cre‐*S*_*2*_‐locus and Cgr‐*S*_*2*_‐locus) share a common large inversion (Fhi‐*S*_*2*_‐locus: 171501–176731) located between *FhS*_*2*_ *‐SLF2* and *Fh1g01700*. In addition, compared to the Fhi‐*S*_*2*_‐locus, the three *S*_*2*_ haplotypes have 26 small deletions and 12 small insertions with a total length of 39.9 kb (Figure 5c).

Phylogenetic analysis revealed that all the *SLF* alleles located at *S*‐locus in citrus could be divided into 14 types (Figure S8; Table S13). Translocations, inversions and tandem repeats in *SLF* alleles surrounding the *S‐RNase* were identified; these may be caused by SVs in the *S*‐locus (Table S12). All 14 types of *SLF* alleles were found in the Fhi‐*S*_*2*_‐locus of *F. hindsii* and there was no significant difference in the expression of these alleles compared to *SLF* alleles in other *S*‐locus (Figure S9a,b). Thus, although there were many SVs in self‐compatible Fhi‐*S*_*2*_‐locus relative to other SI haplotypes, they did not appear to affect gene content or expression and they do not seem to be responsible for the SC phenotype in *F. hindsii*. The MITE insertion in the promoter of the *S*_*2*_ *‐RNase* allele was unique to *F. hindsii* and took place after the divergence of genera in citrus. Our data suggest that the fixation and propagation of the MITE insertion in the Fhi‐*S*_*2*_‐locus was responsible for the loss of SI in the *F. hindsii* population.

### MITE insertions are extensive in citrus and are associated with a SC phenotype

There are multiple possible routes for the loss of SI. However, to date, only one route has been found in citrus, which involved the inactivation of the *S‐RNase* (*S*_*m*_ *‐RNase*), which led to the loss of SI from mandarin and its hybrids (*Citrus* genus) (Liang *et al*., 2020; Zhao *et al*., 2022). Here we have identified a new, alternative route for loss of SI in *Fortunella* genus, which was caused by the insertion of a MITE in the promoter region. We examined whether this phenomenon is widespread. An alignment of *S*‐locus shows that there are many SVs in the same *S* haplotypes of different intergeneric and interspecific citrus, which is consistent with the *S* haplotypes evolving in parallel during the species differentiation of citrus (Figure 5c,d). Moreover, these SVs contain many repeated sequences and TEs, including the MITE insertion found in the *FhiS*_*2*_ *‐RNase* gene promoter of *F. hindsii*, which we identified as being responsible for the loss of SI in *Fortunella*.

**Figure 6:**
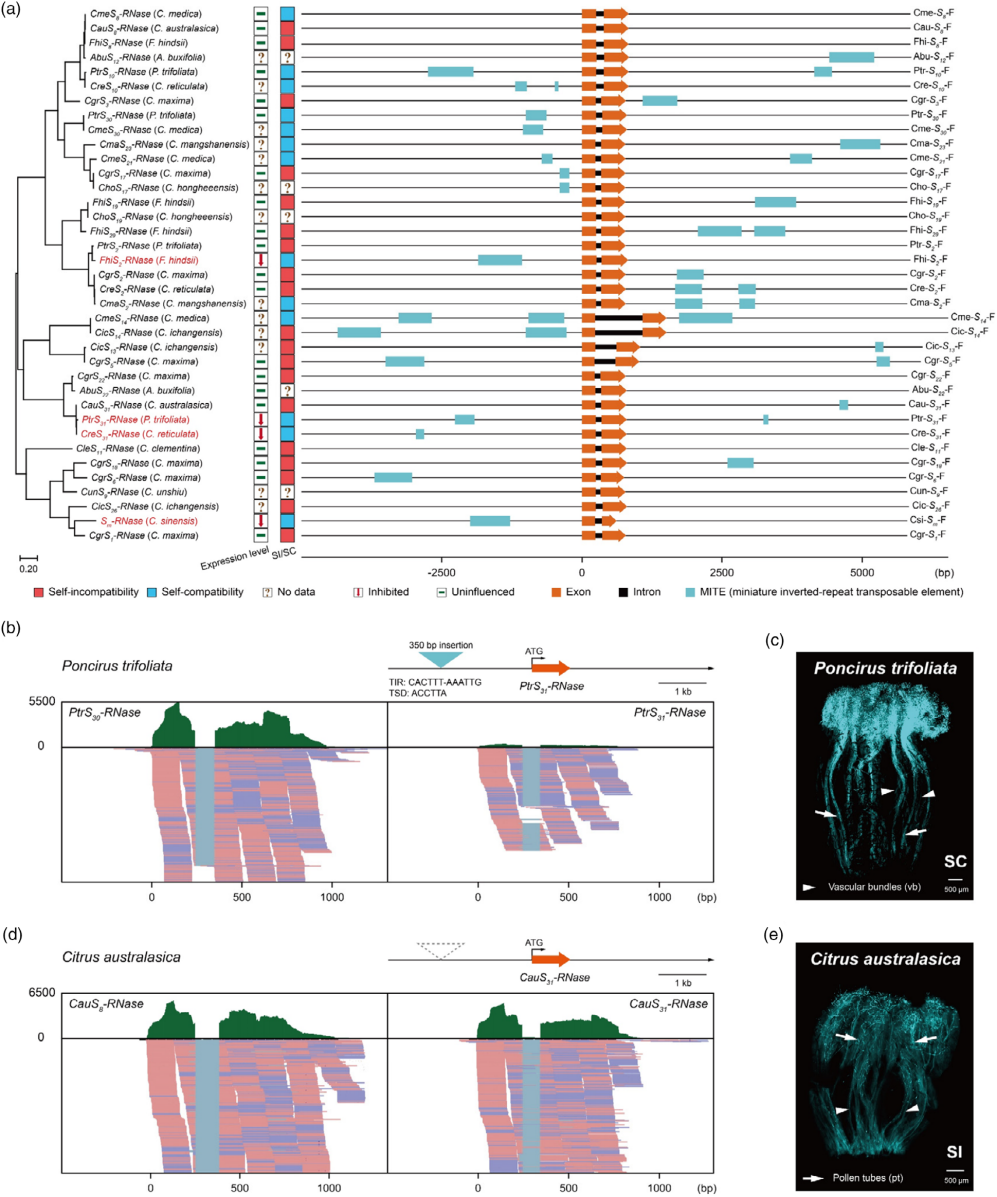
MITE insertions are extensive in citrus and are associated with a SC phenotype. (a) Analysis of MITE insertions in the 5‐kb flanking regions of *S‐RNase* alleles. Detailed information is provided for 37 genomic fragments from different *S* haplotypes of citrus that were analysed. MITE annotation in the 5‐kb flanking regions of *S‐RNase* alleles were made using RepeatMasker (4.1.2), MITE_Hunter (Han and Wessler, 2010) and the P‐MITE database (Chen *et al*., 2014) (see Table S15 for detailed information). Orange blocks represent *S‐RNase* alleles, cyan represents MITE transposons (right). A neighbour‐joining phylogenetic tree was constructed using the amino acid sequences of *S*‐RNases and MEGA X (Kumar *et al*., 2018). Red text indicates SC haplotypes whose expression of *S‐RNase* is inhibited due to the MITE insertion (left). Different colour boxes represent SC or SI haplotypes. A downward red arrow indicates significant inhibition of *S‐RNase*, a short green horizontal line indicates normal *S‐RNase* allele expression, and a question mark in the box indicates a lack of data (middle). (b) Sequence read clusters from the *PtrS*_*30*_ *‐RNase* and *PtrS*_*31*_ *‐RNase* (*P. trifoliata*) alleles. The sequence read clusters are from the RNA‐Seq data generated from the styles of *P. trifoliata* and are shown in the Integrative Genomics Viewer (Robinson *et al*., 2011). There were significantly more reads mapped to *PtrS*_*30*_ *‐RNase* than *PtrS*_*31*_ *‐RNase* in the styles of *P. trifoliata*. (c) Representative aniline blue staining images from pollen tubes in the pistils of *P. trifoliata*. Five images were acquired 5 days after pollination for aniline blue stained pistils at stage −1 DBA (1 day before anthesis) for each combination of pollination. Representative fluorescence images are shown. (d) Sequence read clusters from the *CauS*_*8*_ *‐RNase* and *CauS*_*31*_ *‐RNase* (*C. australasica*) alleles. The sequence read clusters are from the RNA‐Seq data generated from the styles of *C. australasica* and are shown in the Integrative Genomics Viewer (Robinson *et al*., 2011). The reads mapped to the *CauS*_*8*_ *‐RNase* and *CauS*_*31*_ *‐RNase* alleles were equivalent in the styles of *C. australasica*. The green bars depict the number of reads mapped to the *S‐RNase* sequences that included 500‐bp of 5′‐flanking sequence, exons, introns and 500‐bp of 3′‐flanking sequence). A partial alignment of the RNA mapping data is shown (below). Pink and blue represent the reads from the different strands. (e) Representative aniline blue staining of self‐pollinated styles from an *C. australasica* accession collected at Huazhong Agriculture University. Styles from five pollinations from each accession were examined. The growth of the pollen tubes was inhibited near the top of the styles, which is a SI phenotype. Bunches of pollen tubes extending through the pistil is a SC phenotype. Scale bars = 500 μm. Pollen tubes (pt) are indicated with arrows. Vascular bundles (vb) are indicated with arrowheads.

To investigate the MITE insertions of adjacent *S‐RNase* alleles in different citrus populations, we obtained 37 ~ 10.7‐kb genomic fragments that cover the entire *S‐RNase* allele, including 5 kb from the 5′‐flanking region and 5 kb from the 3′‐flanking region, using published citrus genomes data and SMRT sequencing (The sequence fragments can be found in Tables S11, S14 and S15). The MITE annotation was performed using the Repeatmasker Web Server (http://www.repeatmasker.org/cgi‐bin/WEBRepeatMasker), MITE‐Hunter (Han and Wessler, 2010) and the P‐MITE database (Chen *et al*., 2014). A total of 37 annotation results were obtained. These MITEs were mainly derived from three subfamilies and their lengths ranged from 91 to 953 bp (Table S15). The MITE annotation indicates that the MITE insertions near *S‐RNase* alleles occurred frequently in different intergeneric and interspecific of citrus (Figure 6a). We, therefore, examined if they might have a similar effect on SI as that found in *F. hindsii*, which led to the loss of SI in the *Fortunella* population.

Nucleotide alignments revealed the presence of a 350‐bp sequence containing a MITE in the promoter region of the *PtrS*_*31*_ *‐RNase* in SC *P. trifoliata* (Table S15; Figures S10a,b and S11). The expression levels of the *PtrS*_*31*_ *‐RNase* in the styles of *P. trifoliata* were significantly lower relative to other alleles and aniline blue staining showed that *P. trifoliata* was SC (Figure 6b,c). We also found that, in contrast, *Citrus australasia* and *Citrus ichangensis* which are SI, lacked the 350‐bp sequence containing a MITE and the expression levels of *AbuS*_*31*_ *‐RNase* in the styles of *C. australasia* were similar to another allele (*AbuS*_*8*_ *‐RNase*) (Figure 6d,e). This is consistent with our interpretation that the presence of a 350‐bp MITE‐containing sequence in the promoter region is responsible for a SC phenotype. Furthermore, in *Citrus reticulata*, the ‘Mashuiju’ and ‘Mingliutianju’ mandarins are SC, despite not having the *S*_*m*_ *‐RNase* frameshift mutation previously shown to be responsible for the loss of SI in *Citrus* genus (Liang *et al*., 2020). We identified another MITE insertion (115‐bp sequence) in the upstream region of the *CreS*_*31*_ *‐RNase*, which accompany significantly lower expression levels in the style (Figures S11 and S12A). We also identified a MITE insertion in the promoter of the *S*_*m*_ *‐RNase* allele, which was accompanied by significantly reduced expression of the *S*_*m*_ *‐RNase* in the style relative to the other *S* allele (Figure 6a; Figure S12b; Table S15). We also found a 961‐bp MITE insertion in the 3′‐flanking regions of *CicS*_*14*_ *‐RNase* allele in SC *Citrus medica* (Figures S13 and S14). Together, this suggests that MITE insertions occur frequently adjacent to *S‐RNase* alleles and only certain MITEs appear to be responsible for its attenuated expression and a SC phenotype.

The automatic advantage of selfing favour the spread of SC haplotypes in populations, and we previously showed that the *S*_*m*_ *‐RNase* is prevalent (90/153) in 153 citrus accessions (Goldberg *et al*., 2010; Liang *et al*., 2020). We, therefore, examined the frequency of different *S‐RNase* genotypes. We found that the *FhiS*_*2*_ *‐RNase* with the MITE insertion responsible for conferring SC in the *Fortunella* population occurs at a high frequency (Figure 2b; Table S4), which is consistent with the notion that SC is advantageous. We examined 113 accessions of *P. trifoliata* and found that the number of polymorphisms in the *S‐RNase* alleles was generally low (~12 *S‐RNase* genotypes, with the exception of the *PtrS*_*31*_ *‐RNase*, whose promoter contained a MITE insertion accompanied by low expression of this *S‐RNase*, which had a high frequency (89.38%, Figure S15a). In the 127 *C. medica* accessions examined, the frequency of SC *CmeS*_*14*_ *‐RNase* and *CmeS*_*21*_ *‐RNase* alleles were also relatively high (Figure S15b). As we identified MITE insertions in the 3′‐flanking and promoter regions of *CmeS*_*14*_ *‐RNase* and *CmeS*_*21*_ *‐RNase*, respectively, this high frequency could be attributed to the SC phenotype caused by the MITE insertion. However, it should be noted that not all MITEs inserted near *S*‐*RNase* alleles influence expression, as illustrated by the *CgrS*_*2*_ *‐RNase, CgrS*_*3*_ *‐RNase, CgrS*_*5*_ *‐RNase, CgrS*_*6*_ *‐RNase, CreS*_*2*_ *‐RNase* (Hu *et al*., 2021; Liang *et al*., 2020), *FhiS*_*19*_ *‐RNase, FhiS*_*29*_ *‐RNase* (Figure S4), *PtrS*_*10*_ *‐RNase* (Figure S16), *PtrS*_*30*_ *‐RNase* (Figure 6b) and *CauS*_*31*_ *‐RNase* alleles (Figure 6d). Our findings indicate that a large number of TEs, including MITEs, were inserted and enriched around the *S*‐locus, were inserted randomly near *S*‐RNase alleles and evolved in parallel throughout intergeneric and interspecific of citrus (Table S15).

### MITE‐mediated SI loss is a more general phenomenon in *S*‐RNase‐based SI systems

**Figure 7:**
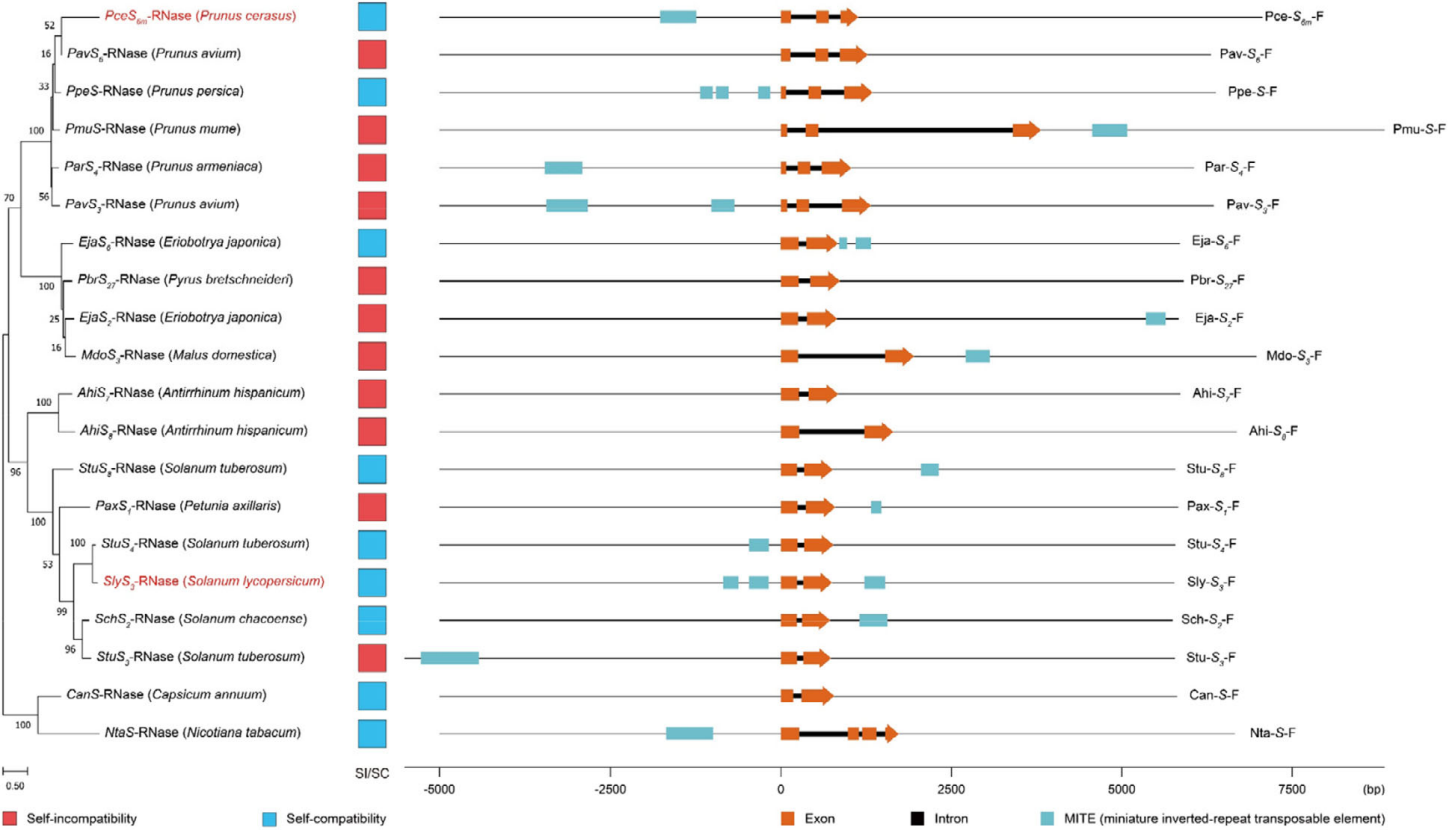
MITE insertions identified in the 5‐kb flanking regions of *S‐RNase* alleles form three common eudicot families (Solanaceae, Plantaginaceae, Rosaceae). The sequences of twenty genomic fragments from different *S* haplotypes of fifteen GSI species from three common eudicot families (Solanaceae, Plantaginaceae, Rosaceae) that utilize the *S*‐RNase SI system were analysed. A neighbour‐joining phylogenetic tree was constructed using the amino acid sequences of *S*‐RNases and MEGA X (Kumar *et al*., 2018) (left). Annotation of MITEs occurring in the 5‐kb flanking regions of *S‐RNase* alleles was made using RepeatMasker (v 4.1.2, https://www.repeatmasker.org/), MITE_Hunter (Han and Wessler, 2010) and the P‐MITE database (Chen *et al*., 2014) (see Table S16 for details). Orange blocks indicate *S‐RNase* alleles, cyan indicate MITE transposons (right). Red boxes and blue boxes (left) indicate SI and SC haplotypes, respectively. Red text indicates SC haplotypes whose expression of *S‐RNase* is known to be inhibited (Kondo *et al*., 2002b; Yamane *et al*., 2003).

To investigate the MITE insertions of adjacent *S‐RNase* alleles in other GSI families, we examined the sequences from fifteen GSI species that utilize the *S*‐RNase SI system. The data revealed the presence of MITE insertions adjacent to the *S‐RNase* alleles were also observed in GSI families. We found that in species known to be SC, MITE insertions were generally found to be located in the promoter region of the *S‐RNase* genes, while in the SI species either MITEs were absent or inserted further away from the *S‐RNase* (Figure 7; Table S16). This is consistent with our findings in citrus, suggesting that MITE‐mediated SI loss may be a more general phenomenon in *S*‐RNase‐based SI systems.

In summary, here we demonstrate for the first time that MITEs provide an important mechanism that is responsible for loss of SI. Our study, shows that MITE insertions in the promoter regions of *FhiS*_*2*_ *‐RNase* and *PtrS*_*31*_ *‐RNase* reduce *S‐RNase* expression and transgenic lines demonstrate that removal of the 786‐bp MITE insertion restores the SI phenotype of *F. hindsii*. We further show that the propagation of this 786‐bp MITE in the *FhiS*_*2*_ *‐RNase* promoter region is responsible for the SC phenotype in a population of *Fortunella*. Our study suggests that this MITE insertion led to the loss of SI in *Fortunella* and *Poncirus* genera, respectively, and that MITEs are likely to have played an important role in SI to SC transitions (Figure S17), as these transposons were inserted frequently at the *S*‐locus in several SI species that utilize the *S*‐RNase system.

## Discussion

Transposable elements (TE) are a rich source of genetic variation in plant genomes, which can be fixed and propagated by selection and can mediate phenotypic diversity (Feschotte *et al*., 2002). It is well established that the insertion of TEs can cause extensive changes in gene composition and function and this can play an important part in plant adaptation and evolution (Chuong *et al*., 2017; Lisch, 2013). The development of genomics and phenomics has made it possible to systematically identify and evaluate the role that TEs have potentially played. For example, the insertion of a terminal‐repeat retrotransposon in miniature (TRIM) element upstream of *Ms2* allele resulted in a male sterility phenotype in wheat (Xia *et al*., 2017); drought tolerance in maize was closely associated with the insertion of a MITE in the promoter of Zm*NAC111* (Mao *et al*., 2015); studies on rice have shown that the susceptibility of rice to bacterial blight was related to the TE insertion in the intron of *WRKY45‐1* (Zhang *et al*., 2016); in potato, the insertion of a 533‐bp MITE in the promoter of *Sli* (*S*‐locus inhibitor) enables its expression in pollen, resulting in the breakdown of SI (Eggers *et al*., 2021; Ma *et al*., 2021). Studies have shown that TEs have contributed to the regulation and evolution of important traits in plants, such as the red coloration of fruit peels (Zheng *et al*., 2019), the formation of bloody sarcocarp (Butelli *et al*., 2012; Lisch, 2013) and apomixis (Shimada *et al*., 2018; Wang *et al*., 2017) in citrus.

Studies on TE distribution in the genomes suggest that chromosomal regions with low recombination frequencies are associated with accumulation of TEs (Kent *et al*., 2017). The *S*‐locus, which determines SI, has long been thought to be a genomic region with suppressed recombination (Boyes *et al*., 1997; Boyes and Nasrallah, 1993; Vieira *et al*., 2003). Therefore, a feature of the *S*‐locus is the occurrence of repetitive sequences and transposable elements in this region. The *S*‐locus of *Arabidopsis lyrata*, *Brassica oleracea*, *Ipomoea trifida*, *Antirrhinum*, *Petunia inflata* and *Prunus dulcis* all exhibit a higher TE density than the genomic background (Fujimoto *et al*., 2006; Goubet *et al*., 2012; Lai *et al*., 2002; Tomita *et al*., 2004; Ushijima *et al*., 2003; Wang *et al*., 2004). In *Prunus* there are several reports of TE insertion in the coding region of the pollen *S*‐determinant *SFB* alleles resulting in loss of function. For example, in *Prunus cerasus* there is an insertion of a 615‐bp Ds‐like element into *SFB*_*1*_ *’* (Hauck *et al*., 2006); in *P. persica* there is a 155‐bp direct repeat inserted in *SFB*_*1*_ (Tao *et al*., 2007); in *P. avium* and *P. mume* there is a 6.8‐kp LTR‐like TE insertion in *SFB* ^*f*^) (Ushijima *et al*., 2004); a *FaSt* MITE insertion disrupted the open reading frame in *SFB*_*C*_ allele in *Prunus* (Halász *et al*., 2014). In contrast to the impact caused by TE insertions in the coding region of *S*‐locus resulting in the loss of SI through deletion or inactivation of *S* determinants (Broz and Bedinger, 2021; Claessen *et al*., 2019; Nasrallah, 2017), the role of TE insertion in the non‐coding region of *S*‐locus is far more ambiguous and has rarely been reported before (Kondo *et al*., 2002a; Tsukamoto *et al*., 2010). Here, we established that MITE transposons are enriched in the non‐coding region of the *S*‐locus. Our data provide the first evidence that a MITE insertion in the promoter region of the female *S* allele can disrupt SI by affecting the expression levels of adjacent *S‐RNase* alleles, thereby causing a SC phenotype.

The breakdown of SI and shift from SI to SC is one of the most common and frequent evolutionary transitions in flowering plants (Goldberg *et al*., 2010). Natural selection tends to switch from outcrossing (SI) to self‐fertilization (SC) because of the inherent reproductive assurance of SC. Indeed, it has been shown that SI was frequently lost during species differentiation, which may involve duplication or mutation of the *S‐RNase*, *SLF* or *non*‐*S*‐determinant genes (Broz and Bedinger, 2021; Claessen *et al*., 2019; Wu *et al*., 2013). However, our understanding of the mechanisms responsible for SI breakdown is still relatively incomplete. Although it is known that SI status can be influenced by genetic modifiers that are unlinked to the *S*‐locus, mutations in the genes comprising the male and female *S* determinants are usually responsible for the loss of SI (Baldwin and Schoen, 2017; Li *et al*., 2016). In this study, we established that a 786‐bp MITE insertion in the promoter of the *S*_*2*_ *‐RNase* allele is responsible for inhibited of *S‐RNase* expression, resulting in the loss of SI in *Fortunella* genera. This seems to be a common occurrence in citrus, since the expression of other *S‐RNase* alleles in other intergeneric or interspecific populations are affected in a similar manner by MITE insertions in their promoters. Our data implicate that these transposons may be responsible for the transition from SI to SC and provide an example of parallel evolution in different populations.

Due to the *S*‐locus being highly polymorphic, the major *cis*‐acting elements and *trans*‐acting factors that influence the expression of the *S*‐locus are poorly understood. In the *S*‐RNase‐based SI system, current research has focused in‐depth on the function of the *S‐RNase*/*SLF* genes and the mechanisms of SI (Broz and Bedinger, 2021; Wu *et al*., 2013); there have been few studies on the relative expression levels of the *S‐RNase*/*SLF* genes since the original studies establishing the *S*‐RNase SI system (Hu *et al*., 2021; McClure *et al*., 1989; Murfett *et al*., 1994; Tsukamoto *et al*., 2003). Previous transgenic studies in tobacco have shown an increase in *S*_*A2*_ *‐RNase* transcripts was associated with increased style RNase activity (Murfett *et al*., 1994) and in antisense *S*_*A2*_ *‐RNase*‐transformed plants, a decrease in *S*_*A2*_ *‐RNase* transcripts was accompanied by decreased style RNase activity (Murfett *et al*., 1995). RNase activity in style tissue was positively correlated with ability to reject self‐pollen (Murfett *et al*., 1994) and a high level of *S‐RNase* expression in style tissues is necessary for functional *S*‐RNase‐based SI (Lee *et al*., 1994; Murfett *et al*., 1994). In this study, we found that the expression level of *S‐RNase* in style tissues was correlated with its ability to reject self‐pollen. The insertion of the MITE in the promoter region resulted in a low expression level of *S‐RNase* in styles and a SC phenotype. Our transgenic data showed that the transcript level of *S‐RNase* reached about 75% of the endogenous *S* allele and that this was sufficient for rejection of self‐pollen.

We also found that MITEs were significantly enriched around the *S‐RNase* alleles in Aurantioideae and were more likely to be inserted in the promoter region of the SC haplotypes. Moreover, this finding has also been confirmed in other GSI families that utilize the *S*‐RNase SI system. In three common eudicot families (Solanaceae, Plantaginaceae, Rosaceae), there are marked differences in the occurrence of MITEs adjacent to the *S‐RNase* alleles in various SI and SC species; in species known to be SC, MITE insertions were generally found to be located in the promoter region of the *S‐RNase* genes, while in the SI species either MITEs were absent or inserted further away from the *S‐RNase*. In addition, previous studies have shown that the low expression level of *S‐RNase* alleles lead to the loss of SI in sour cherry (*Prunus cerasus*) and cultivated tomato (*Solanum lycopersicum*) (Kondo *et al*., 2002b; Yamane *et al*., 2003). Surprisingly, we also found MITE insertions in the promoter region of these two *S‐RNase* genes (*PceS*_*6m*_ *‐RNase* and *SlyS*_*3*_ *‐RNase*), which may affect the expression of host genes and mediate the loss of SI. In conclusion, studies on the four common GSI families confirm that MITE‐mediated SI loss is a more general phenomenon in *S*‐RNase‐based SI systems.

It is important to note that not all MITE insertions around *S*‐RNase alleles could mediate the loss of SI. We suggest that the insertion positions and types of MITEs may affect expression of *S*‐determinant *S‐RNase* genes at the *S*‐locus, as extensive TE insertions at various locations in *FLC* in *Capsella rubella* has been shown to affect *FLC* expression and led to natural variation in flowering time (Niu *et al*., 2019). It is possible that the insertion of TEs might introduce new regulatory elements that may alter the expression of host genes (Lisch, 2013; Shen *et al*., 2017; Xia *et al*., 2017; Zhang *et al*., 2020). This clearly deserves further investigation. In summary, our identification of a role for MITEs in citrus and other GSI families significantly improves how the transition from SI to SC can be achieved.

It is known that the fixation and propagation of the SC haplotypes, which have a reproductive assurance in a population increases the frequency of these genotypes and we show that this happens in SC accessions (Halasz *et al*., 2007; Tsuchimatsu *et al*., 2017). Here, we found that multiple nonfunctional SC haplotypes (such as Fhi‐*S*_*2*_, Ptr‐*S*_*31*_, *S*_*m*_ and Cre‐*S*_*31*_ haplotypes) caused by a MITE insertion were maintained in the citrus species‐wide loss of SI. All these SC haplotypes observed were associated with insertion of TEs; this is quite different from the loss of SI caused by multiple independent recombination of *S* haplotypes in *Arabidopsis* (Tsuchimatsu *et al*., 2017). Analysis of the insertion events of different TEs and the statistics of *S* haplotype frequencies demonstrate that these SC haplotypes were not derived from their ancestors, but arose independently after intergeneric differentiation and were then fixed in the population. Similar to *Capsella rubella* (Guo *et al*., 2011), the SC loci were also under strong positive selection in the population. The widespread apomixis of citrus may also contribute to the fixation and maintenance of multiple independent nonfunctional SC haplotypes in the population (Liang *et al*., 2020; Wang *et al*., 2017), which appears to be unique in adaptive evolution. However, the evolutionary relationship between apomixis and SC is still unclear.

In summary, we identified the first evidence for a pivotal role for MITE insertions in the promoter region at the *S*‐locus reducing the expression of *S‐RNase* alleles, with the consequent breakdown of SI. Our findings are of significance for our understanding of alterations in breeding strategies by SI to SC transitions and the evolution of the *S*‐RNase‐based SI system in angiosperms. Our comprehensive identification of TEs associated with *S*‐locus‐genes provide useful data for evolutionary models proposing the maintenance of high polymorphism of *S* loci and the generation mechanism of SC in different populations (Guo *et al*., 2011; Tsuchimatsu *et al*., 2017). The loss of SI plays a crucial role in the process of adaptive evolution and since an increasing number of functional TEs are being identified, further effects of TEs on species divergence and domestication are likely to be identified in the future.

## Methods

### Plant materials and sample extraction

The hybrid population of *F. hindsii* containing approximately 500 individual plants derived from a PN02 × DB02 cross was generated in 2013 and was planted in the germplasm garden at the National Citrus Breeding Center at Huazhong Agricultural University. PN02 (*S*_*8*_ *S*_*19*_) is self‐incompatible and DB02 (*S*_*2*_ *S*_*29*_) is self‐compatible. Two individual plants (PD02‐229 and PD02‐482) from this population were used for segregation ratio analysis by genotyping the *S*‐locus in the S_1_ progeny derived from self‐pollinated individuals. The 46 accessions of *F. hindsii* that were used in this study were collected from 5 provinces in China. Details on these accessions are provided (Table S4). Floral organs from PN02 and DB02 were collected and divided into six tissues (petals, anthers, filaments, styles, ovaries and receptacle) that were frozen immediately in liquid nitrogen and stored at −80 °C for further analysis. Fresh leaf tissues were used for genomic sequencing.

### Evaluation of self‐compatibility

Nearly opened flowers from each individual in the hybrid population were collected. Pollen was obtained by manually separating and drying the anthers at 28 °C for 12 h. At the flowering stage, approximately 10 flowers (1 day before anthesis) were self‐pollinated on each individual plant using a fine paint brush and covered with a paper pollination bag. The pollinated styles were collected after 48 h. Aniline blue staining was performed as described by Liang *et al*., (2017). In addition, for the visualization of pollen tube growth in hardy orange, mandarin and Australian limes, pollinated styles were collected after 96–120 h. Selected styles were imaged using Inverted Microscopes for Industry Leica DMi8 M. The images were made using the associated software package (Public Experimental Platform of Key Laboratory of Horticultural Plant Biology, HZAU, MOE).

More than 5 carpels were taken from each individual for observation. The carpels were scored as SC if more than 50% of the pollen tubes reached the ovary and are indicated with a ‘√’. If the pollen tubes stopped growing in the upper third of the stigma, the carpel was scored as incompatible and indicated with an ‘×’. Other phenotypes were scored as uncertain and indicated with a ‘‐’. If 3 or more styles were compatible, the individual was scored as self‐compatible. If all styles were scored as incompatible, the individual was scored as self‐incompatible. In other cases, the individual was scored as undetermined.

### Bulked segregant analysis sequencing (BSA‐Seq)

PN02 was crossed with DB02 to generate F_1_ individuals that segregated the SC and SI phenotypes at a 1:1 ratio. DNA was extracted from pools of tissue collected from 32 self‐incompatible F_1_ individuals and 32 self‐compatible F_1_ individuals. Two 300‐bp libraries were constructed to generate 150‐bp paired‐end short reads (90×) using the BGISEQ‐500 platform (BGI‐Shenzhen, China). Clean reads were obtained by quality evaluation using fastp (v. 0.23.1) (Chen *et al*., 2018). Subsequently, the filtered short reads were aligned against the reference genome (S3y‐45 v2.0) (Wang *et al*., 2022). The variant calling of SNPs was excavated using the GATK (v. 4.1.2.0) (McKenna *et al*., 2010) software. The heterozygous and inconsistent SNPs between the two DNA pools were selected by calculating G' values using the G prime algorithm in the QTLseqr package (Li and Xu, 2021; Mansfeld and Grumet, 2018).

### Kompetitive allele‐specific PCR (KASP) analysis

KASP genotyping reactions (KASP‐TF V4.0 2 × Master Mix 384, Low ROX) for allelic discrimination was performed on a Roche LightCycler® 480 II (Roche, Switzerland) using KASP assays designed to be specific for SNPs from the target region. KASP assays were conducted according to the protocol supplied by the manufacturer (LGC, Laboratory of the Government Chemist, UK) with FAM (Excitation: 465 nm；Emission: 510 nm) and HEX (Excitation: 533 nm; Emission: 580 nm). The genotyping results after the standard KASP thermal cycle were analysed and visualized using the lightCycler® software (Endpoint Genotyping program) to confirm correct segregation and genotype. KASP markers used in this article are listed in Table S17.

### Identification of selection signatures

For the selection scan, we used the imputed SNP data set of 46 accessions (Table S8) that was controlled using VCFtools (v. 0.1.16) (Danecek *et al*., 2011). The integrated haplotype score (iHS) test was performed to investigate selection signatures of SC‐associated loci in the SC *F. hindsii* population. The iHS statistic compares the Extended haplotype homozygosity (EHH) between two alleles by controlling for the allele frequency of each SNP. The R library rehh (Gautier and Vitalis, 2012) was used to calculate EHH and iHS statistic. Cross‐Population Extended Haplotype Homozygosity (XP‐EHH) statistics based on the extended haplotype was calculated for SC and SI *F. hindsii* populations (Table S8) using selscan (v. 2.0.0) (Szpiech and Hernandez, 2014). The SC *F. hindsii* was defined as observed population, and the SI *F. hindsii* was the reference population. Significant genomic regions were identified by *P*‐value <0.001.

### Quantitative RT‐PCR analysis

Total RNA was extracted with RNAiso Plus (TaKaRa, Japan) according to the manufacturer's instructions. cDNA was synthesized with HiScript® II Q RT SuperMix for qPCR (+gDNA wiper) (Vazyme Biotech, China) according to the supplier's protocol. RT‐PCR was conducted as described by Hu *et al*. (2021). All samples were run in triplicate, and there were two separate runs for each plate. For qRT‐PCR, expression of *β‐actin* was used as an internal control for normalization. The relative gene expression was calculated using the 2^‐ΔΔCt^ method. The pertinent primers are listed in Table S17.

### GUS assays

The cloned promoter fragments (*FhiS*_*2*_ *‐RNase*_*Pro*_, 2689 bp; *FhiS*_*2*_ *‐RNase*_*ProΔMITE*_, 1903 bp) were constructed in pKGWFS7 using the Gateway method (Invitrogen, USA). The recombinant plasmids were transformed into DB02 callus that had been subcultured for 21 days in the dark using the *Agrobacterium*‐mediated method. For GUS staining, callus was submerged in X‐gluc buffer (X‐Gluc, 50 mg; 50 mM/L, pH 7.0, PBS, 80 mL; 20% methanol, 20 mL; 0.5 M/L EDTA, 2 mL; 1% Triton‐100, 1 mL) overnight at 37 °C, de‐stained with 70% ethanol and photographed.

### Dual‐luciferase assays

The promoter fragments (*FhiS*_*2*_ *‐RNase*_*ProΔMITE*_, 1903 bp; *PtrS*_*2*_ *‐RNase*_*Pro+MITE*_, 2688 bp; *FhiS*_*2*_ *‐RNase*_*Pro*_, 2689 bp; *PtrS*_*2*_ *‐RNase*_*Pro*_, 1902 bp) were inserted into the pGreenII 0800‐*LUC* vector to drive the expression of the *firefly luciferase* (*LUC*) gene using the ClonExpress II One Step Cloning Kit (Vazyme, China). The recombinant plasmids were transformed into DB02 callus and cocultured at 21 °C for 3 days in constant darkness on MT‐Acetosyringone (AS) medium (4.46 g/L Murashige and Tucker Medium [PhytoTechnology Laboratories, LLC], 40 g/L sucrose, 8 g/L agar, 20 mg/L AS, pH 5.8). The activity of LUC and REN was measured using the Dual‐Luciferase® Reporter Assay System (Promega, USA). Promoter activity was quantified as the ratio of LUC/REN with 12 replicates. Relevant primers are listed in Table S17.

### *Agrobacterium*‐mediated genetic transformation

The binary plasmid vectors pK7WG2D.1 containing p*35S*::*FhiS*_*2*_ *‐RNase* and DsRED2 containing p*FhiS*_*29*_::*FhiS*_*2*_ *‐RNase* and p*FhiS*_*2ΔMITE*_::*FhiS*_*2*_ *‐RNase* were provided by Prof. Jin (Sun *et al*., 2018) and were transferred into *Agrobacterium tumefaciens* strain EHA105. Surface sterilized mature seeds were inoculated on MT medium and cultured at 25 °C in the dark. After 6–8 weeks, the seedings were transferred to a photoperiod containing 16 h of light and 8 h of dark for 14 days. After chlorophyll visibly accumulated, the seedings were cut into 1–1.5 cm long stem segments that were used for transformation experiments. *Agrobacterium*‐mediated genetic transformation of *F. hindsii* was performed as described by Zhu *et al*. (2019). The transgenic lines were identified using PCR with specific primers (Table S17).

### Genomic and transcriptomic sequencing

The sequencing of PN02 and DB02 was performed using the PacBio Sequel II platform (Pacific Biosciences, USA) at Beijing Biomarker Technologies Co. Ltd. (Beijing, China). A SMRTbell library with a size of 30 kb was constructed for genomic sequencing following the standard protocol provided by Pacific Biosciences. Two 300‐bp libraries were constructed to survey the genome information using the BGISEQ‐500 platform (BGI‐Shenzhen, China). Total RNA was extracted from the anther and style tissues of PN02 and DB02. Paired‐end libraries were constructed and sequenced on BGISEQ‐500 platform (BGI‐Shenzhen, China). Three biological replicates were analysed.

### RNA‐seq analysis

The anther and style tissues of PN02 and DB02 were collected 1 day before anthesis. Clean reads were mapped to the reference genome (S3y‐45 v2.0) using Hisat2 (Kim *et al*., 2019) with default parameters. The TPM (transcripts per kilobase per million mapped reads) of each gene and the DEGs (fold change ≥2, FDR <0.01) between PN02 and DB02 were calculated (Love *et al*., 2014). The reproducibility of transcriptome samples was assessed using a Spearman rank correlation.

### Whole‐genome bisulphite sequencing (WGBS) and analysis

BS‐seq libraries from leaves, anthers and style tissues of DB02 were constructed and sequenced using 150‐bp reads from the BGISEQ‐500 platform (BGI‐Shenzhen, China). The clean reads were aligned to the DB02gv1 contig genome using Bismark (v. 0.22.2) (Krueger and Andrews, 2011), allowing up to three mismatches. Only cytosines that were covered by at least four reads were considered and used for the calculation. The methylation levels of individual cytosines were calculated as the ratio of mC to the total cytosines [mC/(mC + un‐mC)].

### Small RNA transcriptome analysis

Small RNAs were extracted from anther and style tissues 1 day before anthesis using RNAiso Plus (TaKaRa) according to the manufacturer's instructions. Then the 18–30 nt small RNAs were excised and ligated to adenylated 3′ adapters annealed to unique molecular identifiers (UMI), followed by the ligation of 5′ adapters. Library preparation and single‐end sequencing were conducted using the DNBSEQ platform (BGI‐Shenzhen, China). After filtering, the clean reads were mapped to the *F. hindsii* genome (S3y‐45 v2.0) or within the *S*‐locus in DB02 using Bowtie (Langmead *et al*., 2009) (v. 1.2.3) with the following parameters: a ‐m 20 ‐v 0 ‐p 1. The remaining parameters were set at default values. Mapped reads were extracted using the SAMtools (Li *et al*., 2009) (v. 1.14) suite with default parameters. The alignment results were visualized using the Integrated Genome Viewer (Robinson *et al*., 2011) (v. 2.11.3). The RMP (reads per million) of siRNAs in the target region between anther and style were calculated.

### Genome assembly and *S* haplotype reconstruction

The CLR reads were assembled using Canu (Koren *et al*., 2017) (v2.1.1) with the following parameters: ‐‐ pacbio (minReadLength = 2000, minOverlapLength = 500, corOutCoverage = 150, corMinCoverage =2 ‘batOptions = ‐dg 3 ‐db 3 ‐dr 1 ‐ca 500 ‐cp 50’, corrected error rate = 0.035). The reads were polished iteratively using two rounds of Pilon (Walker *et al*., 2014) (v. 1.24) with ~12 Gb of WGS BGISEQ data to generate the draft genome (PN02gv1 contig, DB02gv1 contig and AZMgv1 contig). The sequences of *S* haplotypes were identified from genomes using the *S‐RNase* alleles. The Fhi‐*S*_*8*_‐locus, Fhi‐*S*_*19*_‐locus, Fhi‐*S*_*2*_‐locus, Fhi‐*S*_*29*_‐locus, Cau‐*S*_*8*_‐locus and Cau‐*S*_*31*_‐locus were obtained using the 200‐bp sequences from two non‐recombinant KASP markers (K961397 and K1247997). Gene predictions and annotations for each *S*‐locus were made using FGENESH (Solovyev *et al*., 2006) and nonredundant protein sequence (nr) (Pruitt *et al*., 2007) databases. The sequences of the other *S*‐locus were obtained from the published genome (see Table S14 for more details). The syntenic regions of the *S*‐locus were identified using the all‐vs‐all BLASTP (e‐value = 1e‐5) method with a threshold value of 0.95.

### Whole‐sequence alignments and structural variation analysis

Pairwise alignments of all *S*‐locus were performed using the NUCmer program in MUMmer (Marcais *et al*., 2018) (v.4.0.0). The Fhi‐*S*_*2*_‐locus in the S3y‐45 v2.0 genome (*F. hindsii*) were used as a reference. The filtering parameters were as follows: ‘Delta‐filter‐i 90‐L 200‐1’. The filtered data were plotted using Mummerplot. Show‐diff and show‐SNPs were used for structural variation (SVs, > 50 bp). show‐ SNPs in MUMmer was used to detect SNPs between the reference and query sequences. The parameters were as follows: ‘‐c‐H‐I‐T‐r‐L’. The online website Assemblytics (Nattestad and Schatz, 2016) (http://assemblytics.com/) was used to analyse and visualize structural variation.

### Isolation and identification of MITEs

The *S* haplotypes in the genomes were identified by BLAST alignment with the *S‐RNase* allele sequences (see Table S14 for more details), and genomic fragments covering the entire *S‐RNase* gene, the 5‐kb 5′‐flanking region and the 5‐kb 3′‐flanking region were obtained based on the position of *S* alleles. The genomic fragments sequences data can be found in the GenBank data libraries under accession numbers (Table S15). The MITE annotation was performed using MITE‐Hunter (Han and Wessler, 2010), Repeatmasker Web Server (http://www.repeatmasker.org/cgi‐bin/WEBRepeatMasker) and the P‐MITE database (Chen *et al*., 2014).

## Author contributions

JH and LC conceived the project. JH conducted KASP experiments and performed BSA‐Seq, transcriptomic, genomic, siRNAs and WGBS sequencing and data acquisition. ZD conducted the genome assembly and MITE annotation. CL, FG and DS conducted PCR, GUS and LUC assays. NW, SZ, CZ, PC and QX provided the plant materials. ZW, JJ, ZC and CS collected the plant samples and conducted pollination assays. XD and LC supervised the project and revised the manuscript. JH wrote the manuscript with the help of JY, RL, ZL, MB and VEF‐T. All authors contributed to the article and approved the manuscript before submission.

## Conflict of interest

The authors declare no conflicts of interest.

## Supporting information


**Figure S1** Fluorescence images of pollen tubes in pistils of a *F. hindsii* cross‐pollination population (PN02 × DB02).
**Figure S2.** A signature of selection at the *S* loci.
**Figure S3** Nucleotide and amino acid sequence alignment of *S*_*2*_ *‐RNase* in different intergeneric or interspecific of citrus.
**Figure S4** Analysis of *S‐RNase* alleles expression in style tissues of DB02, PN02, and ZK8.
**Figure S5** Nucleotide sequence alignment analysis of the *S*_*2*_ *‐RNase* promoter between *F. hindsii* and *P. trifoliata*.
**Figure S6** Methylation status of *FhiS*_*29*_ *‐RNase* in the anther and style of *F. hindsii*.
**Figure S7** Transgenic assays with the *FhiS*_*2*_ *‐RNase* gene from *F. hindsii*.
**Figure S8** Phylogenetic tree analysis of SLFs in citrus.
**Figure S9** TPM values for the *SLF* and *S‐RNase* alleles from the *F. hindsii S*‐locus.
**Figure S10** Nucleotide and amino acid sequence alignments of *S*_*31*_ *‐RNase* from different intergeneric and interspecific of citrus.
**Figure S11** Nucleotide sequences alignment of *S*_*31*_ *‐RNase* promoter regions from SC *P. trifoliata*, SI *C. australasia*, SI *C. ichangensis*, and SC *C. reticulata*.
**Figure S12** Expression of *S‐RNase* in the style tissues of SC mandarins.
**Figure S13** Nucleotide and amino acid sequence alignment of *S*_*14*_‐RNase in different intergeneric and interspecific of citrus.
**Figure S14** Nucleotide sequence alignment of the downstream region of the *S*_*14*_ *‐RNase* allele from SI *C. ichangensis* and SC *C. medica*.
**Figure S15** Analysis of the *S*‐haplotype of *P. trifoliata* and *C. medica* accessions.
**Figure S16** Expression of *S‐RNase* in the style tissues in different intergeneric and interspecific of citrus.
**Table S1** Identification of self‐incompatibility/compatibility in a *F. hindsii* cross‐pollination population.
**Table S2** Statistical analysis of SI/SC phenotypes for the progeny from a PN02 × DB02 hybrid population.
**Table S3** KASP genotyping analysis of a *F. hindsii* cross‐pollination population.
**Table S4** Genotype and SC phenotype identification for 46 *F. hindsii* accessions.
**Table S5** List of the significant integrated haplotype score measures (iHS) for the single‐nucleotide polymorphism (SNP) markers and their closest genes.
**Table S6** SC‐related genomic region with mean XP‐EHH value >1 and associated genes.
**Table S7** Mapping summary for WGBS reads.
**Table S8** Summary information of DNA methylation in leaf, anther, and style tissues from DB02.
**Table S9** Summary of small RNA sequencing data for the anther and style tissues from DB02.
**Table S10** List of siRNAs associated with flanking regions of *FhiS*_*2*_ *‐RNase* allele in anther and style tissues (RPM ≥ 2).
**Table S11** Summary statistics for the assembly of 3 genome sequences.
**Table S12** Summary of transposable elements in *S*‐locus of *F. hindsii*.
**Table S13** Isolation and identification of *S*‐locus *F‐box* alleles in citrus.
**Table S14** Detailed information relating to whole‐sequence alignments of *S*‐locus.
**Table S15** Detailed information for MITE annotation in the 5‐kb flanking regions of *S‐RNase* alleles in citrus.
**Table S16** Detailed information for MITE annotation near the S‐RNase genes in three common GSI families.
**Table S17** List of primers used in this study.
**Table S18** Isolation and identification of *S*‐ribonuclease alleles in citrus.
**Table S19** Detailed information relating to checking the *S* alleles for 24 mandarin accessions.

**Figure S17** Multiple routes for the loss of SI in the three major genera (*Citrus*, *Fortunella*, *Poncirus*) of the Aurantioideae.

## Data Availability

All sequence data have been deposited at the NCBI under project number PRJNA860018, PRJNA827463, PRJNA827498. The genome assembly have been deposited in the CNGB Sequence Archive (CNSA) of the China National GenBank DataBase (CNGBdb) with accession numbers CNP0003278. The RNA sequencing data from anther and style tissues are available on the NCBI short read archive under accession PRJNA827480. The BSA‐Seq data has been deposited in NCBI under project number PRJNA827489. Other data are available in the source data file or will be made available upon request. Source data are provided with this paper. *S‐RNase* and *SLF* sequences data from this article can be found in the GenBank data libraries under accession numbers (see Table S13, S15, S16 and S18).
