# Supplementary material for: Transposable elements cause the loss of self‐incompatibility in citrus
Source: Plant Biotechnol J. 2023 Dec 1;22(5):1113–31. doi: 10.1111/pbi.14250 (PMC11022811; doi:10.1111/pbi.14250)
Supplement: Supplementary file 1 — Figure S1 Fluorescence images of pollen tubes in pistils of a F. hindsii cross‐pollination population (PN02 × DB02). Figure S2. A signature of selection at the S loci. Figure S3 Nucleotide and amino acid sequence alignment of S 2 ‐RNase in different intergeneric or interspecific of citrus. Figure S4 Analysis of S‐RNase alleles expression in style tissues of DB02, PN02, and ZK8. Figure S5 Nucleotide sequence alignment analysis of the S 2 ‐RNase promoter between F. hindsii and P. trifoliata. Figure S6 Methylation status of FhiS 29 ‐RNase in the anther and style of F. hindsii. Figure S7 Transgenic assays with the FhiS 2 ‐RNase gene from F. hindsii. Figure S8 Phylogenetic tree analysis of SLFs in citrus. Figure S9 TPM values for the SLF and S‐RNase alleles from the F. hindsii S‐locus. Figure S10 Nucleotide and amino acid sequence alignments of S 31 ‐RNase from different intergeneric and interspecific of citrus. Figure S11 Nucleotide sequences alignment of S 31 ‐RNase promoter regions from SC P. trifoliata, SI C. australasia, SI C. ichangensis, and SC C. reticulata. Figure S12 Expression of S‐RNase in the style tissues of SC mandarins. Figure S13 Nucleotide and amino acid sequence alignment of S 14 ‐RNase in different intergeneric and interspecific of citrus. Figure S14 Nucleotide sequence alignment of the downstream region of the S 14 ‐RNase allele from SI C. ichangensis and SC C. medica. Figure S15 Analysis of the S‐haplotype of P. trifoliata and C. medica accessions. Figure S16 Expression of S‐RNase in the style tissues in different intergeneric and interspecific of citrus. Table S1 Identification of self‐incompatibility/compatibility in a F. hindsii cross‐pollination population. Table S2 Statistical analysis of SI/SC phenotypes for the progeny from a PN02 × DB02 hybrid population. Table S3 KASP genotyping analysis of a F. hindsii cross‐pollination population. Table S4 Genotype and SC phenotype identification for 46 F. hindsii accessions. Table S5 List of the signific [file PBI-22-1113-s002.docx]

# Supplemental Information for：Transposable elements cause the loss of self-incompatibility in citrus

Jianbing Hu ^1,2^, Chenchen Liu ^1,2^, Zezhen Du ^1,2^, Furong Guo ^1^, Dan Song ^1^, Nan Wang ^1^, Zhuangmin Wei ^3^, Jingdong Jiang ^1^, Zonghong Cao ^1^, Chunmei Shi ^1^, Siqi Zhang ^1^, Chenqiao Zhu ^1^, Peng Chen ^4^, Robert M. Larkin ^1,2^, Zongcheng Lin ^1,2^, Qiang Xu ^1,2^, Junli Ye ^1^, Xiuxin Deng ^1,2^, Maurice Bosch ^5^, Vernonica E. Franklin-Tong ^6^, Lijun Chai ^1, 2 *^

^1^ National Key Laboratory for Germplasm Innovation and Utilization of Horticultural Crops, College of Horticulture and Forestry Sciences, Huazhong Agricultural University, Wuhan 430070, P. R. China;

^2^ Hubei Hongshan Laboratory, Wuhan 430070, P. R. China;

^3^ Guangxi Subtropical Crops Research Institute, Nanning 530001, P. R. China;

^4^ Horticultural Institute, Hunan Academy of Agricultural Sciences, Changsha, 410125, China;

^5^ Institute of Biological, Environmental and Rural Sciences (IBERS), Aberystwyth University, Aberystwyth, UK

^6^ School of Biosciences, College of Life and Environmental Sciences, University of Birmingham, Edgbaston, Birmingham, UK.

*Corresponding author:

E-mail: [chailijun@mail.hzau.edu.cn](mailto:chailijun@mail.hzau.edu.cn)

**Supplementary Figures legends**

**Supplementary Figure 1.** Fluorescence images of pollen tubes in pistils of a *F. hindsii* cross-pollination population (PN02 × DB02).

**Supplementary Figure 2.** A signature of selection at the *S* loci.

**Supplementary Figure 3.** Nucleotide and amino acid sequence alignment of *S_2_-RNase* in different intergeneric or interspecific of citrus.

**Supplementary Figure 4.** Analysis of *S-RNase* alleles expression in style tissues of DB02, PN02, and ZK8.

**Supplementary Figure 5.** Nucleotide sequence alignment analysis of the *S_2_-RNase* promoter between *F. hindsii* and *P. trifoliata*.

**Supplementary Figure 6.** Methylation status of *FhiS_29_-RNase* in the anther and style of *F. hindsii*.

**Supplementary Figure 7.** Transgenic assays with the *FhiS_2_-RNase* gene from *F. hindsii*.

**Supplementary Figure 8.** Phylogenetic tree analysis of SLFs in citrus.

**Supplementary Figure 9.** TPM values for the *SLF* and *S-RNase* alleles from the *F. hindsii* *S*-locus.

**Supplementary Figure 10.** Nucleotide and amino acid sequence alignments of *S_31_-RNase* from different intergeneric and interspecific of citrus.

**Supplementary Figure 11.** Nucleotide sequences alignment of *S_31_-RNase* promoter regions from SC *P. trifoliata*, SI *C. australasia*, SI *C. ichangensis*, and SC *C. reticulata*.

**Supplementary Figure 12.** Expression of *S-RNase* in the style tissues of SC mandarins.

**Supplementary Figure 13.** Nucleotide and amino acid sequence alignment of *S_14_*-RNase in different intergeneric and interspecific of citrus.

**Supplementary Figure 14.** Nucleotide sequence alignment of the downstream region of the *S_14_-RNase* allele from SI *C. ichangensis* and SC *C. medica*.

**Supplementary Figure 15.** Analysis of the *S*-haplotype of *P. trifoliata* and *C. medica* accessions.

**Supplementary Figure 16.** Expression of *S-RNase* in the style tissues in different intergeneric and interspecific of citrus.

**Supplementary Figure 17.** Multiple routes for the loss of SI in the three major genera (*Citrus*, *Fortunella*, *Poncirus*) of the Aurantioideae.

**Supplementary Tables legends**

**Supplementary Table 1.** Identification of self-incompatibility/compatibility in a *F. hindsii* cross-pollination population

**Supplementary Table 2.** Statistical analysis of SI/SC phenotypes for the progeny from a PN02 × DB02 hybrid population

**Supplementary Table 3.** KASP genotyping analysis of a *F. hindsii* cross-pollination population

**Supplementary Table 4.** Genotype and SC phenotype identification for 46 *F. hindsii* accessions

**Supplementary Table 5.** List of the significant integrated haplotype score measures (iHS) for the single-nucleotide polymorphism (SNP) markers and their closest genes

**Supplementary Table 6.** SC-related genomic region with mean XP-EHH value > 1 and associated genes

**Supplementary Table 7.** Mapping summary for WGBS reads

**Supplementary Table 8.** Summary information of DNA methylation in leaf, anther, and style tissues from DB02

**Supplementary Table 9.** Summary of small RNA sequencing data for the anther and style tissues from DB02

**Supplementary Table 10.** List of siRNAs associated with flanking regions of *FhiS_2_-RNase* allele in anther and style tissues (RPM ≥ 2)

**Supplementary Table 11.** Summary statistics for the assembly of 3 genome sequences

**Supplementary Table 12.** Summary of transposable elements in *S*-locus of *F. hindsii*

**Supplementary Table 13.** Isolation and identification of *S*-locus *F-box* alleles in citrus

**Supplementary Table 14.** Detailed information relating to whole-sequence alignments of *S*-locus

**Supplementary Table 15.** Detailed information for MITE annotation in the 5-kb flanking regions of *S-RNase* alleles in citrus

**Supplementary Table 16.** Detailed information for MITE annotation near the S-RNase genes in three common GSI families

**Supplementary Table 17.** List of primers used in this study

**Supplementary Table 18.** Isolation and identification of *S*-ribonuclease alleles in citrus

**Supplementary Table 19.** Detailed information relating to checking the *S* alleles for 24 mandarin accessions

**Supplementary Figures**

**
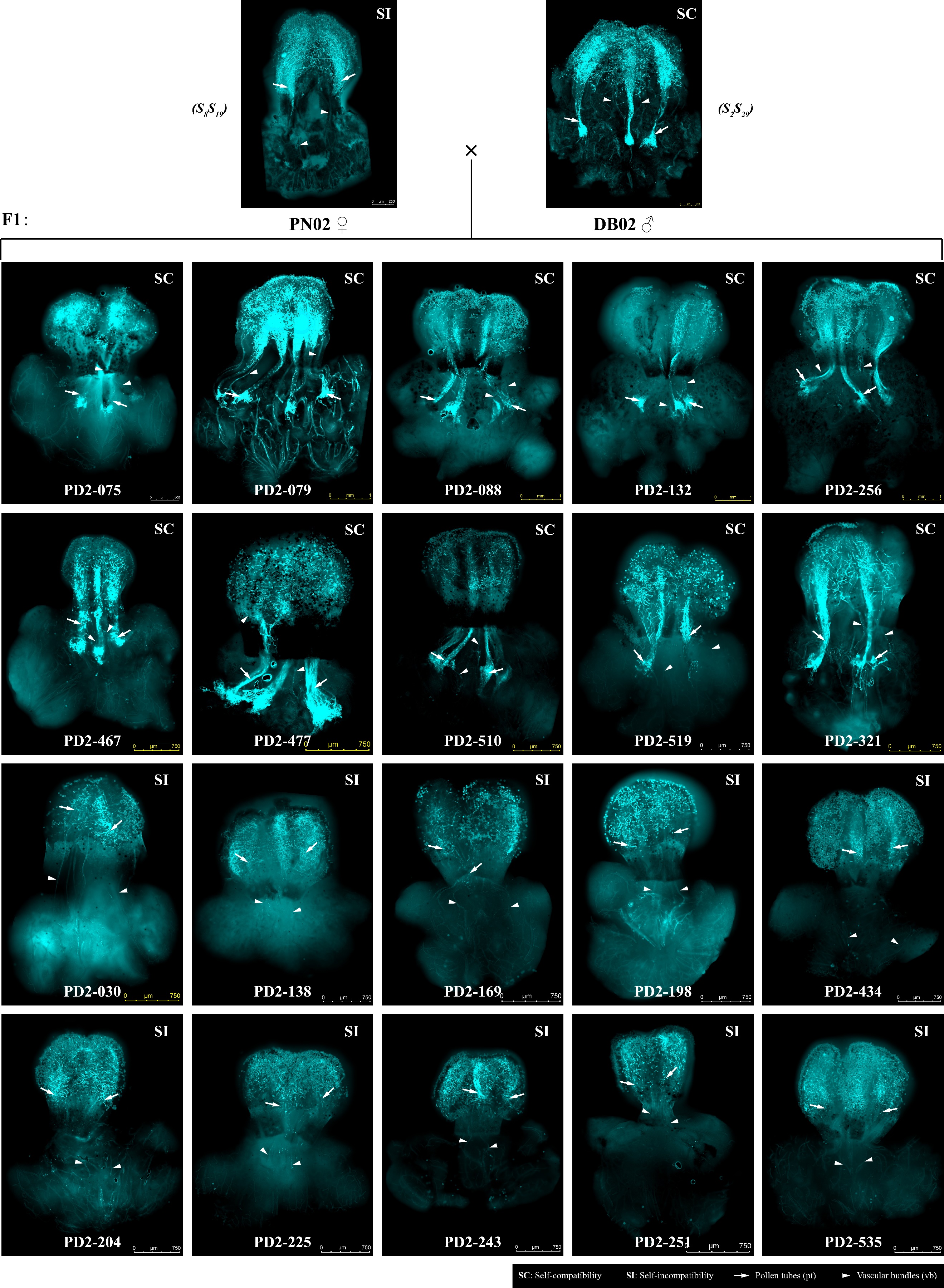
**

**Supplementary Figure 1. Fluorescence images of pollen tubes in pistils of a *F. hindsii* cross-pollination population (PN02 × DB02).**

Images of aniline blue stained pistils were acquired from 5 pistils 2 d after pollination from each pollination combination. Pollination was performed with pistils at stage -1 DBA (1 d before anthesis). Representative images are shown. The accession names are indicated to the bottom.

**
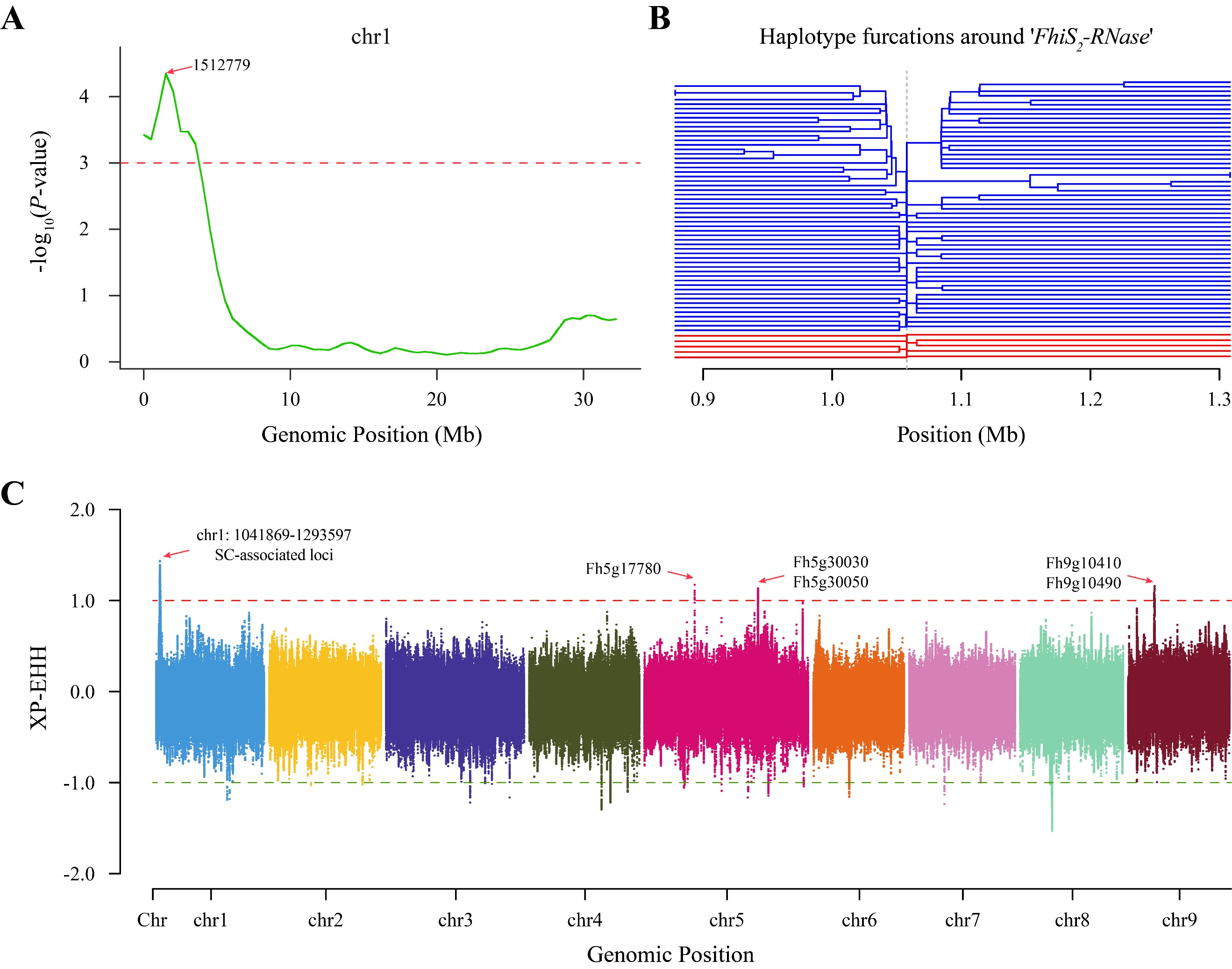
**

**Supplementary Figure 2. A signature of selection at the *S* loci.**

**A：**BSA-Seq analysis of the F_1_ hybrid population derived from a PN02 × DB02 cross. -log_10_ (*P*-value) plotted against the single nucleotide polymorphism (SNP) positions on chromosome 1 of the *F. hindsii* (S3y-45 v2.0 ^1^) genome. **B：**Haplotype bifurcation diagrams for the long haplotype and the other variants in the *FhiS_2_-RNase* region. Line width corresponds to the number of individuals with the indicated haplotype. **C：**The selection signatures detected by Cross Population Extend Haplotype Homozygosity Test (XP-EHH) test. Manhattan plots reflect the chromosome-wide distribution of selection signatures detected by the XP-EHH test in *F. hindsii* population. The SC *F. hindsii* was defined as observed population, and the SI *F. hindsii* was the reference population (see **Supplementary Table 4, 6**). The red arrow indicates a peak above the threshold value.

**
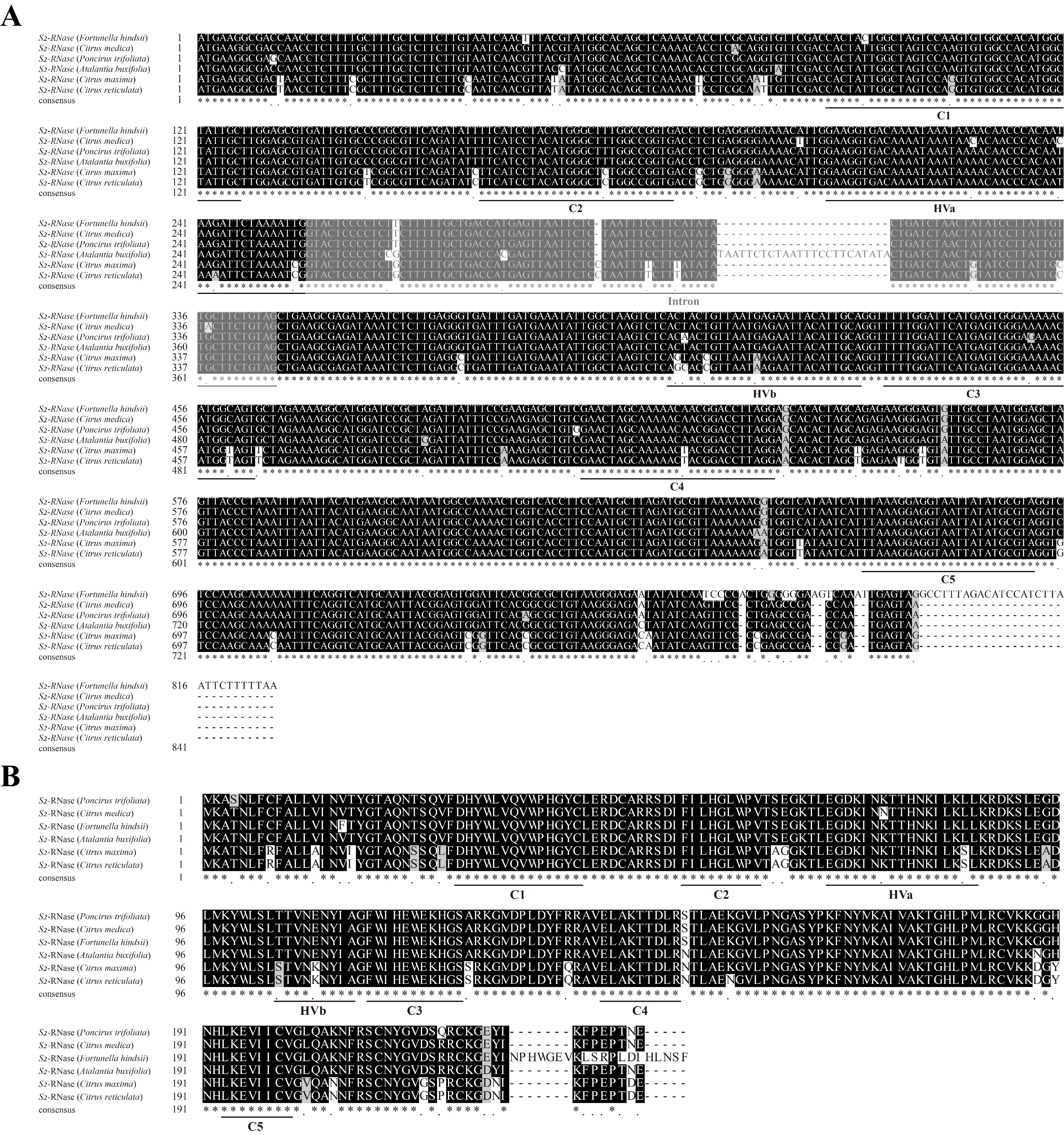
**

**Supplementary Figure 3. Nucleotide and amino acid sequence alignment of *S_2_-RNase* in different intergeneric or interspecific of citrus.**

**A：**DNA sequences of *PtrS_2_-RNase* (*Poncirus trifoliata*), *CmeS_2_-RNase* (*Citrus medica*), *FhiS_2_-RNase* (*Fortunella hindsii*), *AbuS_2_-RNase* (*Atlantia buxifolia*), *CgrS_2_-RNase* (*Citrus maxima*), *CreS_2_-RNase* (*Citrus reticulata*) genes. **B：**Amino acid sequences of *S_2_*-RNases. The nucleotide sequence identities above 80% among *S_2_-RNase* are indicated by shaded boxes. Black indicates 100% conservation. Grey indicates ≥ 80% conservation. Dashes represent gaps. Asterisks indicate 100% conservation. The citrus *S-RNases* contain five conserved domains (C1-C5) and two hypervariable regions (HV1-HV2).

**
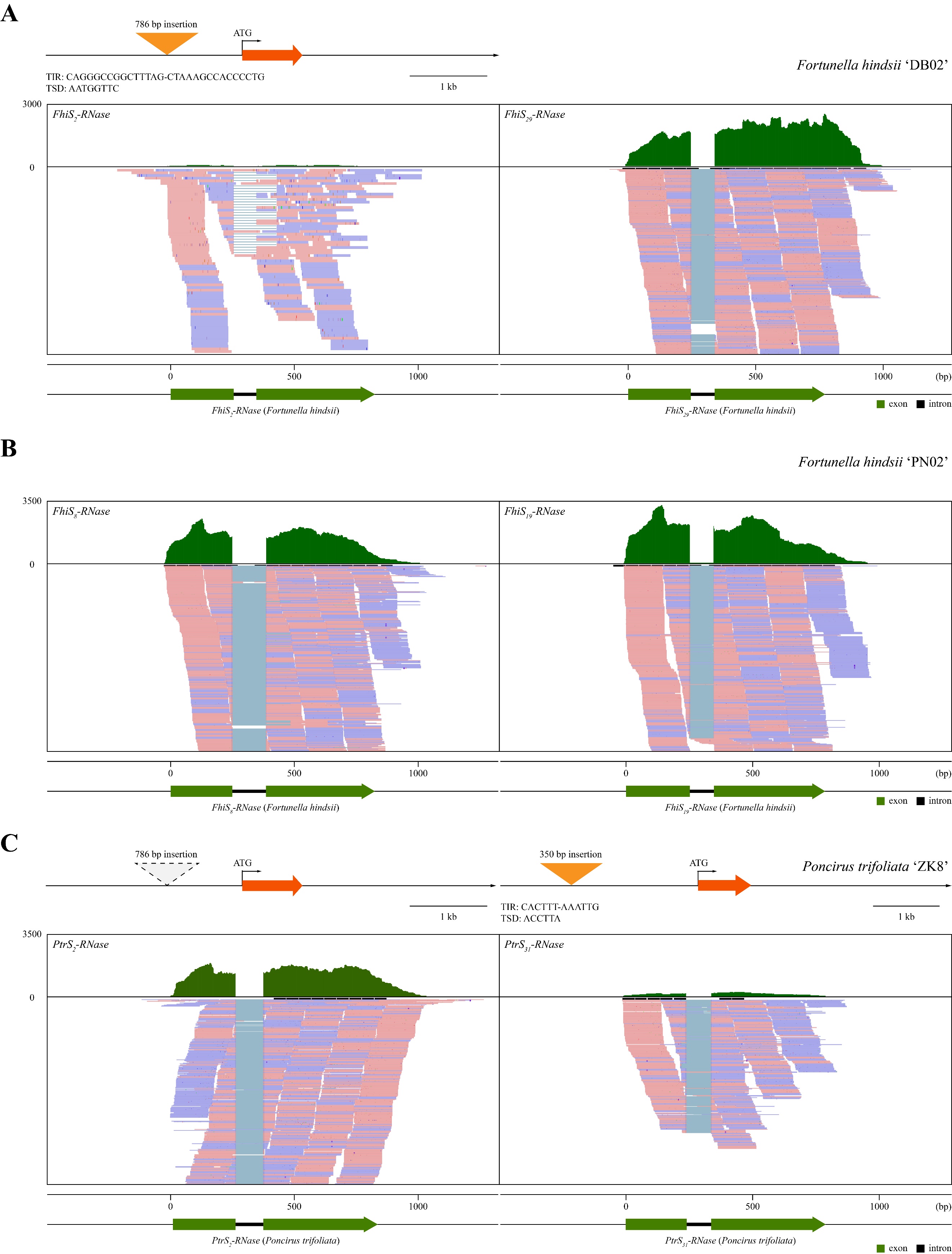
**

**Supplementary Figure 4. Analysis of *S-RNase* alleles expression in style tissues of DB02, PN02, and ZK8.**

**A:** Sequence read clusters from the *FhiS_2_-RNase* and *FhiS_29_-RNase* alleles. The sequence read clusters are from the RNA-Seq data generated from the styles of DB02 *F. hindsii* and are shown in the Integrative Genomics Viewer. The green bars depict the number of reads mapped to the assembled reference genome for the DB02gv1 contig. There were significantly more reads mapped to *FhiS_29_-RNase* than *FhiS_2_-RNase* in the styles of DB02 *F. hindsii*. **B:** Sequence read clusters from the *FhiS_8_-RNase* and *FhiS_19_-RNase* alleles. The sequence read clusters are from the RNA-Seq data generated from the styles of PN02 *F. hindsii* and are shown in the Integrative Genomics Viewer. The green bars depict the number of reads mapped to the assembled reference genome for the PN02gv1 contig. The reads mapped to the *FhiS_8_-RNase* and *FhiS_19_-RNase* alleles were equivalent in the styles of PN02 *F. hindsii*. A partial alignment of the RNA mapping data is shown below. Pink and blue represent the sequences of the different strands of DNA. **C:** Sequence read clusters from the *PtrS_2_-RNase* and *PtrS_31_-RNase* alleles. The sequence read clusters are from the RNA-Seq data generated from the styles of ZK8 (*P. trifoliata*, *S_2_S_31_*) and are shown in the Integrative Genomics Viewer. The green bars depict the number of reads mapped to the assembled reference genome for the *Poncirus trifoliata* v1.0 chromosome ^2^. There were significantly more reads mapped to *PtrS_2_-RNase* than *PtrS_31_-RNase* in the styles of ZK8 *P. trifoliata*.

**
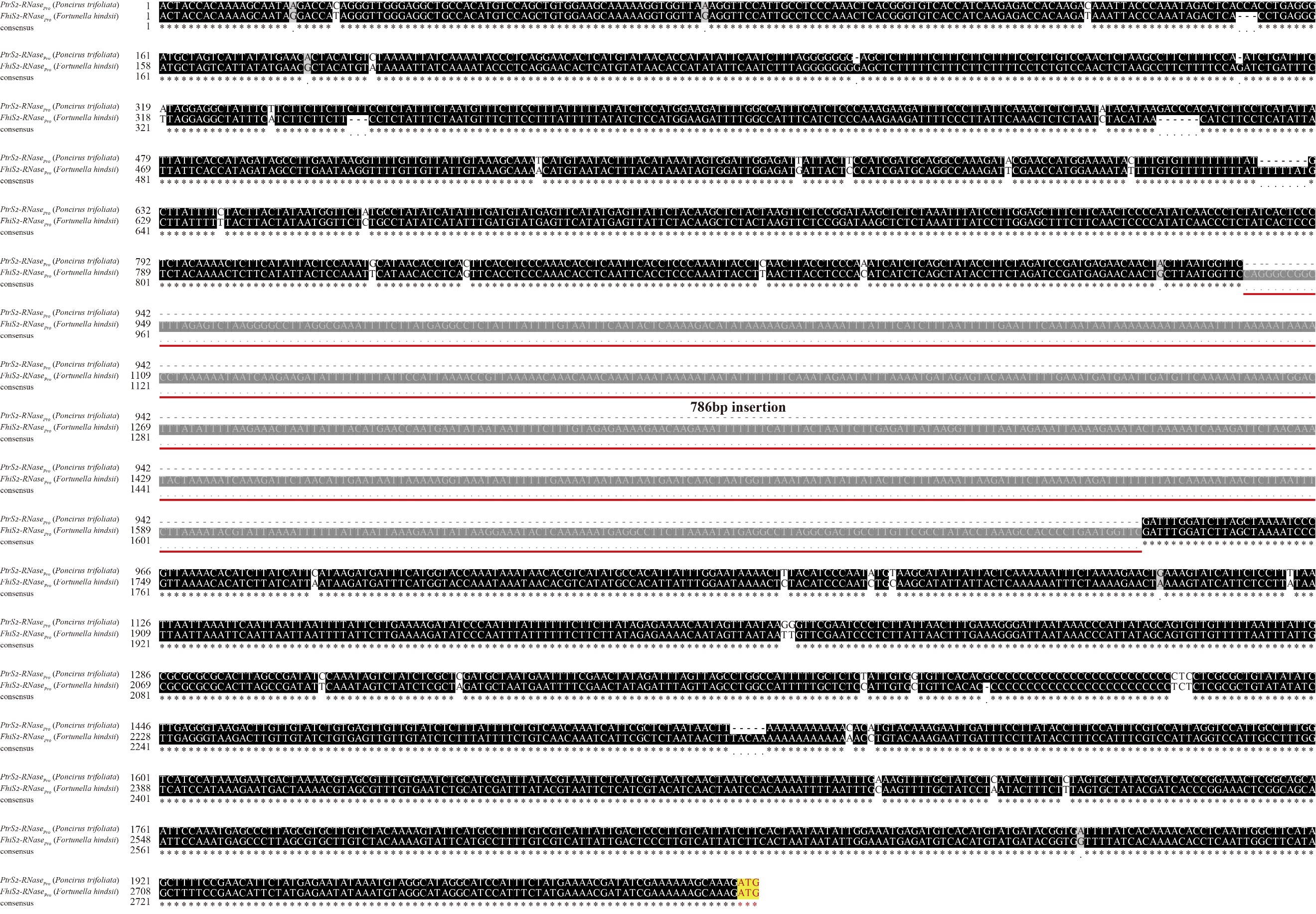
**

**Supplementary Figure 5. Nucleotide sequence alignment analysis of the *S_2_-RNase* promoter between *F. hindsii* and *P. trifoliata*.**

Compared with *F. hindsii*, the upstream promoter region of the *PtrS_2_-RNase* (*P. trifoliata*) gene lacked a 786 bp miniature inverted-repeat transposable element (MITE).

**
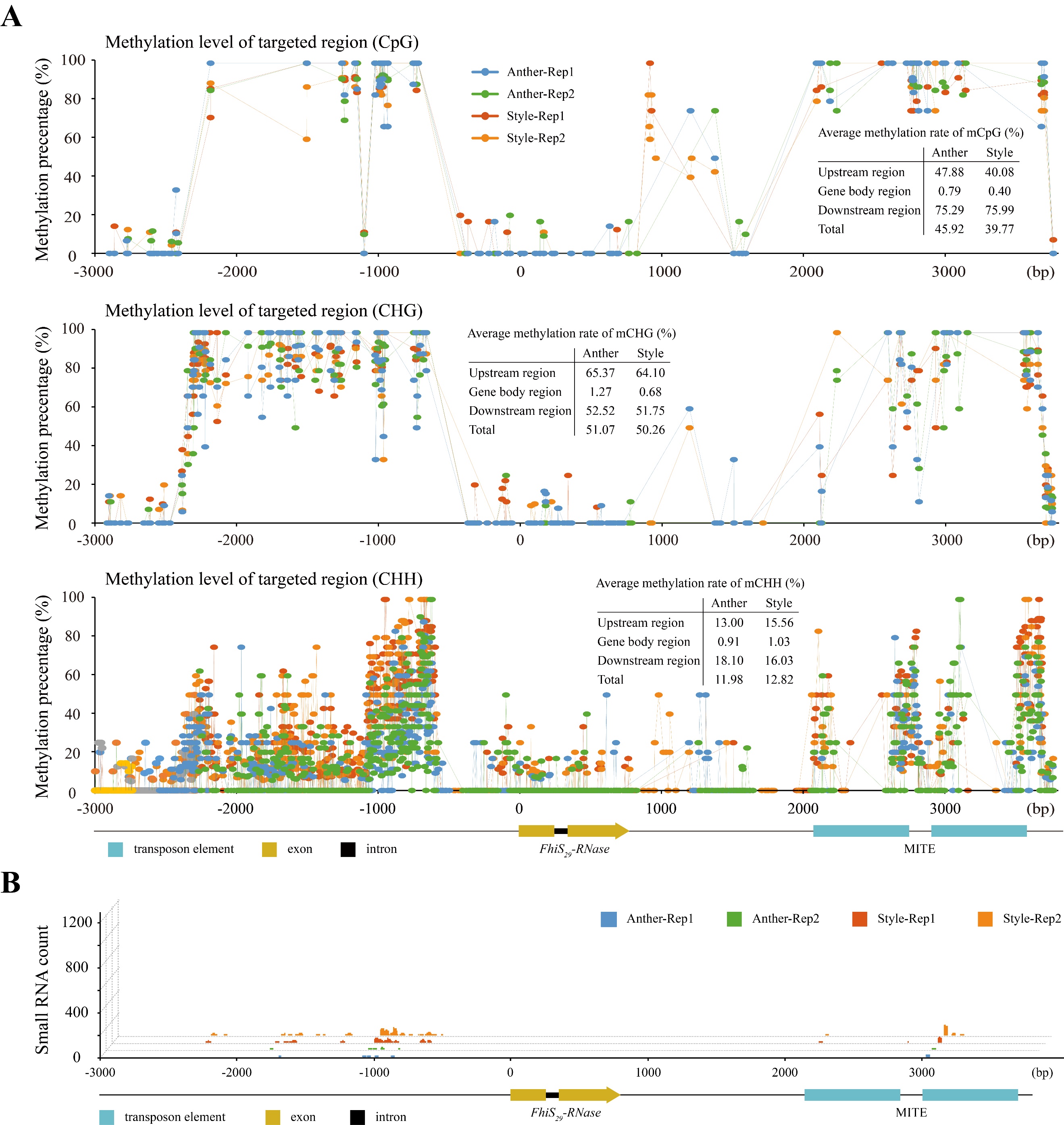
**

**Supplementary Figure 6. Methylation status of *FhiS_29_-RNase* in the anther and style of *F. hindsii*.**

**A:** Methylation levels in the *FhiS_29_-RNase* allele in the leaf, anther, and style of *F. hindsii*. Methylation levels in the CpG (top row), CHG (middle row) and CHH (bottom row) contexts were quantified in 3-kb 5’-flanking regions, exons, introns and 3-kb 3’-flanking regions. The percentage of total mCs is the number of mCs/total number of Cs. **B:** Numbers of small RNAs from anthers and styles that map to the 3-kb 5’-flanking regions of the *FhiS_29_-RNase* allele in DB02.

**
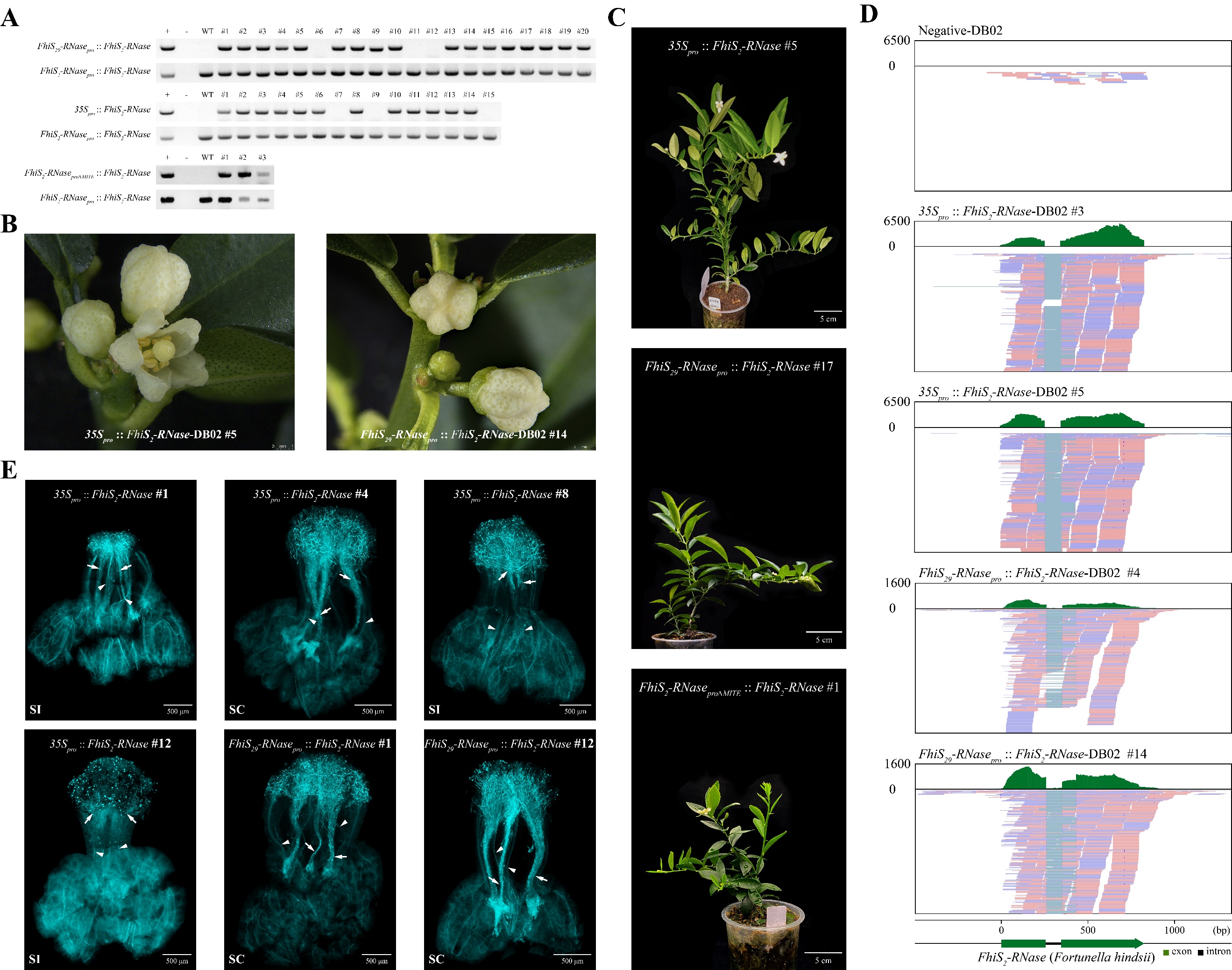
**

**Supplementary Figure 7. Transgenic assays with the *FhiS_2_-RNase* gene from *F. hindsii*.**

**A：**PCR analysis of regenerated plants using specific primers (*35S_pro_*::*FhiS_2_-RNase*, *FhiS_29_-RNase_pro_*::*FhiS_2_-RNase*, and *FhiS_2_-RNase_pro∆MITE_*::*FhiS_2_-RNase*). + and – indicate the positive and negative control. The numbers assigned to the different regenerated lines are shown above each lane. **B：**Morphologies of *F. hindsii* floral organs from transgenic lines, *35S_pro_*::*FhiS_2_-RNase*-DB02 #5 (left), *FhiS_29_-RNase_pro_*::*FhiS_2_-RNase*-DB02 #14 (right). **C：**Morphologies of transgenic plants. The accession numbers of different transgenic lines are shown (above). Scale bars = 5 cm. **D：**Sequence read clusters from the *FhiS_2_-RNase* allele. The sequence read clusters are from the RNA-Seq data generated from the styles of transgenic lines and are shown in the Integrative Genomics Viewer. The green bars depict the number of reads mapped to the assembled reference genome for DB02gv1. There were significantly more reads mapped to *FhiS_2_-RNase* in the styles of transgenic lines than in the styles of negative lines. A partial alignment of the RNA mapping data is shown below. Pink and blue represent the sequences of the different strands. **E：**Representative aniline blue staining images of pollen tubes in pistils from the *35S_pro_*::*FhiS_2_-RNase*-DB02 and *FhiS_29_-RNase_pro_*::*FhiS_2_-RNase*-DB02 transgenic plants. Representative fluorescence images of aniline blue stained pistils are shown. Five images were acquired for each pollination combination. Pollinations were performed at stage -1 DBA (1 d before anthesis). Images were acquired 2 d after pollination. Scale bars = 500 μm. Pollen tubes (pt) are indicated with arrows. Vascular bundles (vb) are indicated with arrowheads.

**
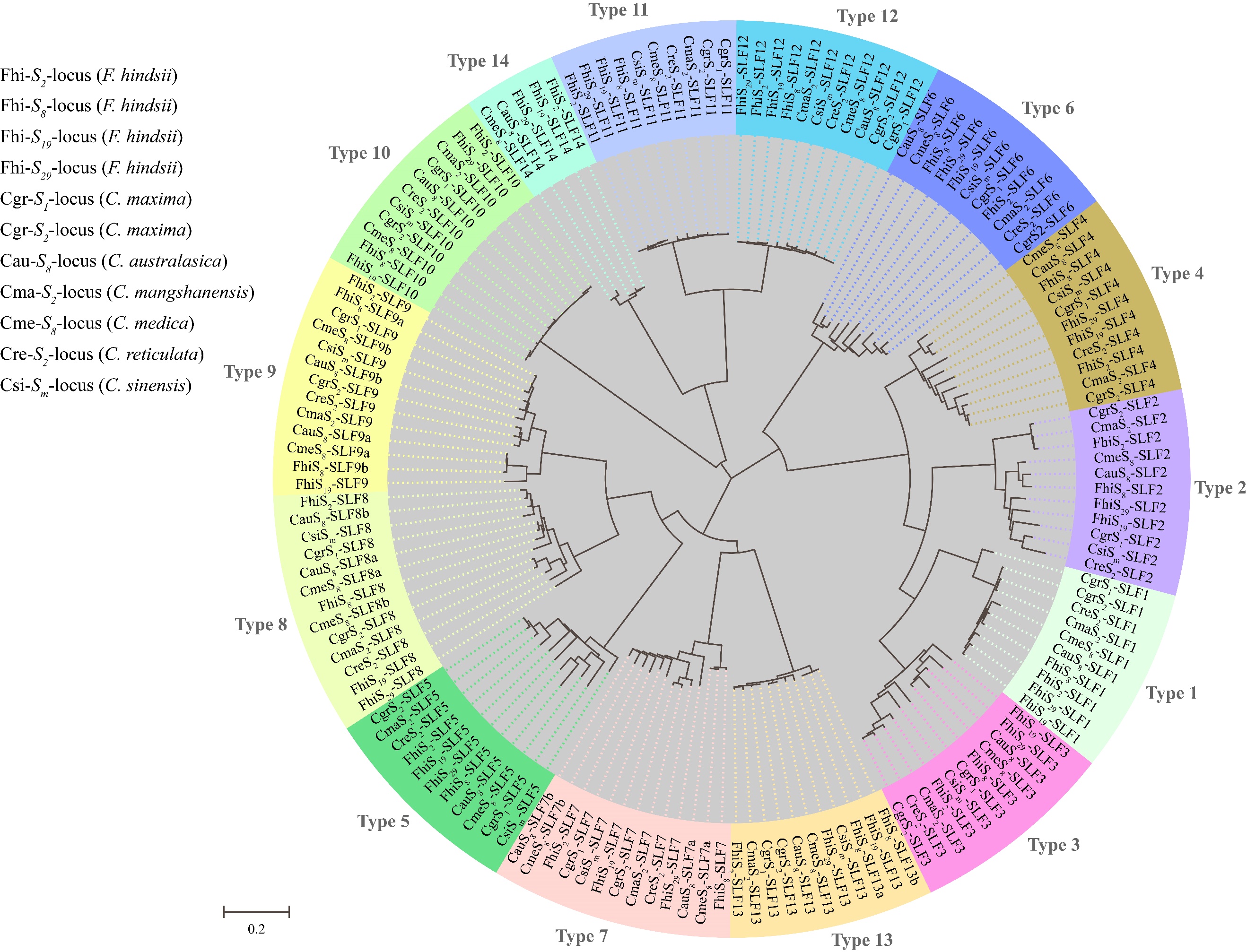
**

**Supplementary Figure 8. Phylogenetic tree analysis of SLFs in citrus.**

ML phylogenetic tree for *SLF* genes from 11 haplotypes from 14 exemplar families with bootstrap confidence values > 50%. The *SLF* alleles from different families are indicated by different branch colors. Detailed information on *S*-locus from different intergeneric and interspecific of citrus is provided (**Supplementary Table 14**).

**
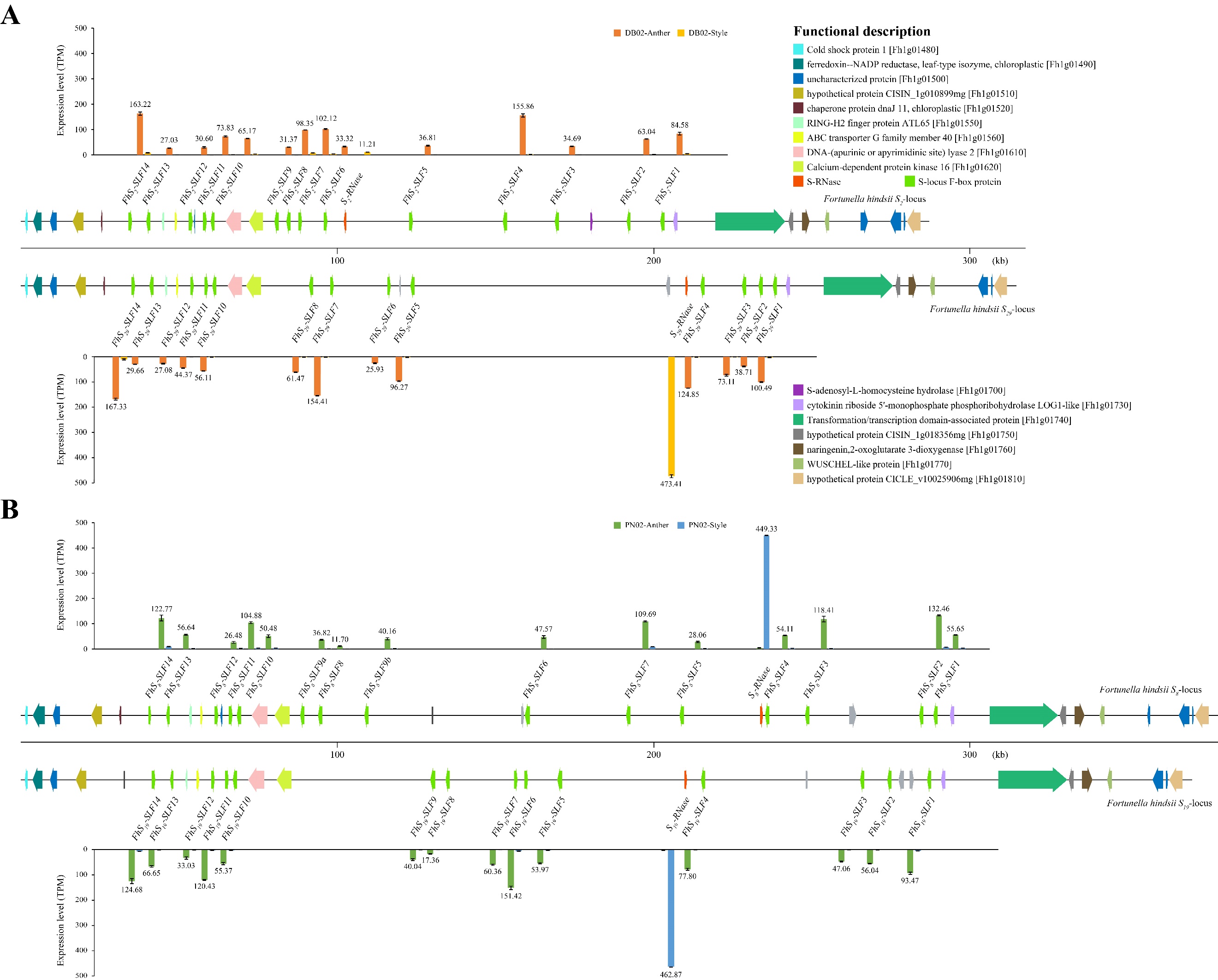
**

**Supplementary Figure 9. TPM values for the *SLF* and *S-RNase* alleles from the** ***F. hindsii* *S*-locus.**

Schematic diagram of the relative positions of annotated genes at the DB02 *S* loci (**A**) and PN02 *S* loci (**B**). The expression levels of genes at the Fhi-*S_2_*-locus, Fhi-*S_29_*-locus, Fhi-*S_8_*-locus, and Fhi-*S_19_*-locus in the style and anther tissues of DB02 and PN02 *F. hindsii* were based on the three biological replicates. TPM values for the *SLF* genes and the *S-RNase* genes in the Fhi-*S_2_*-locus, Fhi-*S_29_*-locus, Fhi-*S_8_*-locus, and Fhi-*S_19_*-locus were calculated with the reads mapped to the DB02 and PN02 assembled reference genome. Error bars indicate mean values ± SE, n = 3. Colored boxes represent different genes associated with the Fhi-*S_2_*-locus, Fhi-*S_29_*-locus, Fhi-*S_8_*-locus, and Fhi-*S_19_*-locus. Orange boxes represent *S*-RNase. Green boxes represent SLFs (see **Supplementary Tables 13, 17** for more details).

**
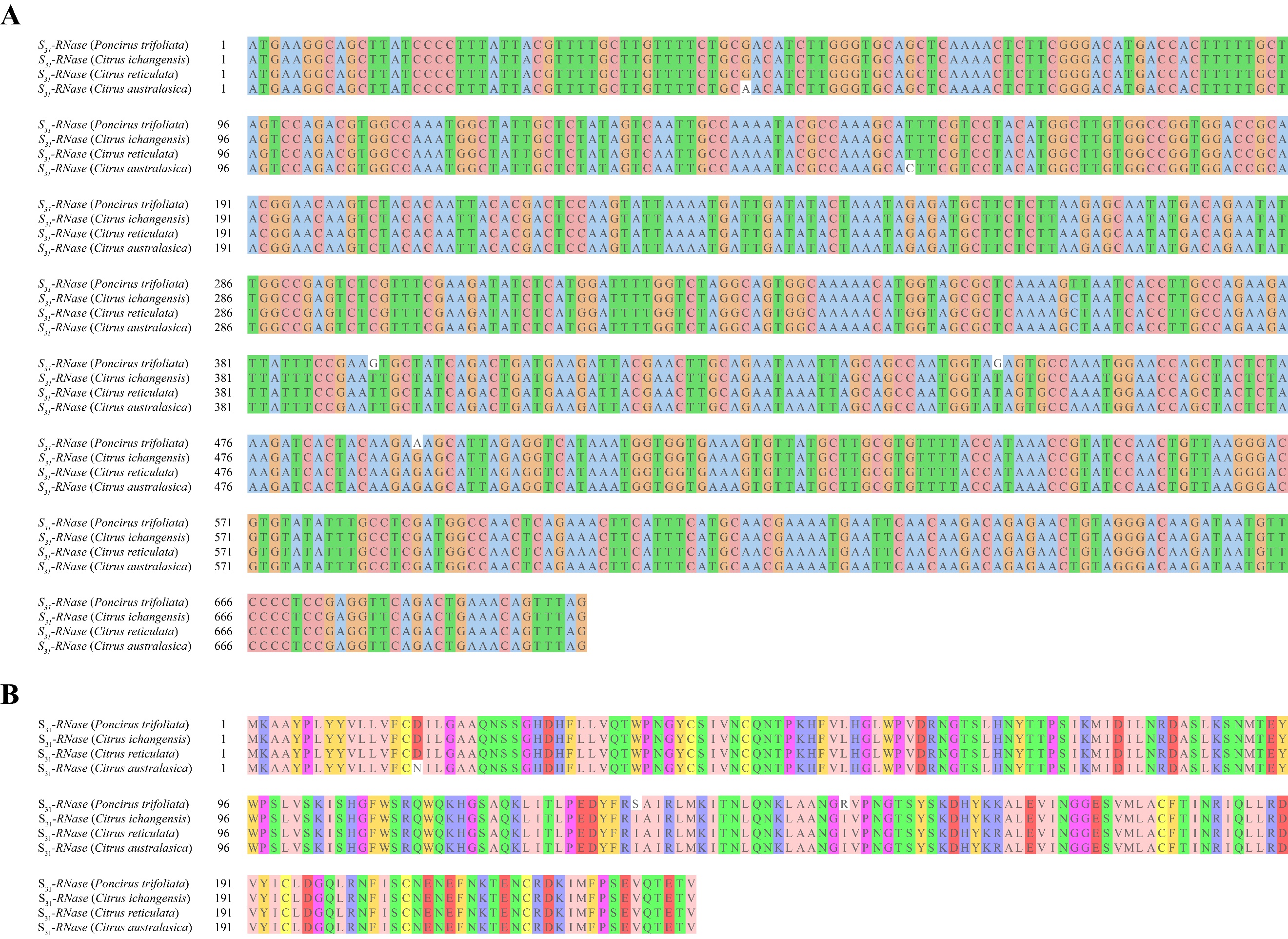
**

**Supplementary Figure 10. Nucleotide and amino acid sequence alignments of *S_31_-RNase* from different intergeneric and interspecific of citrus.**

**A：**CDS sequences of *PtrS_31_-RNase* (*P. trifoliata*, SC), *CicS_31_-RNase* (*C. ichangensis*, SI), *CreS_31_-RNase* (*C. reticulata*, SC), and *CauS_31_-RNase* (*C. australasica*, SI) genes. **B：**Amino acid sequences of *S_31_*-RNases. The nucleotide sequence identities above 80% among *S_31_-RNase* genes are indicated with shaded boxes. The same color indicates 100% conservation. White indicates SNPs with synonymous or non-synonymous mutations.

**
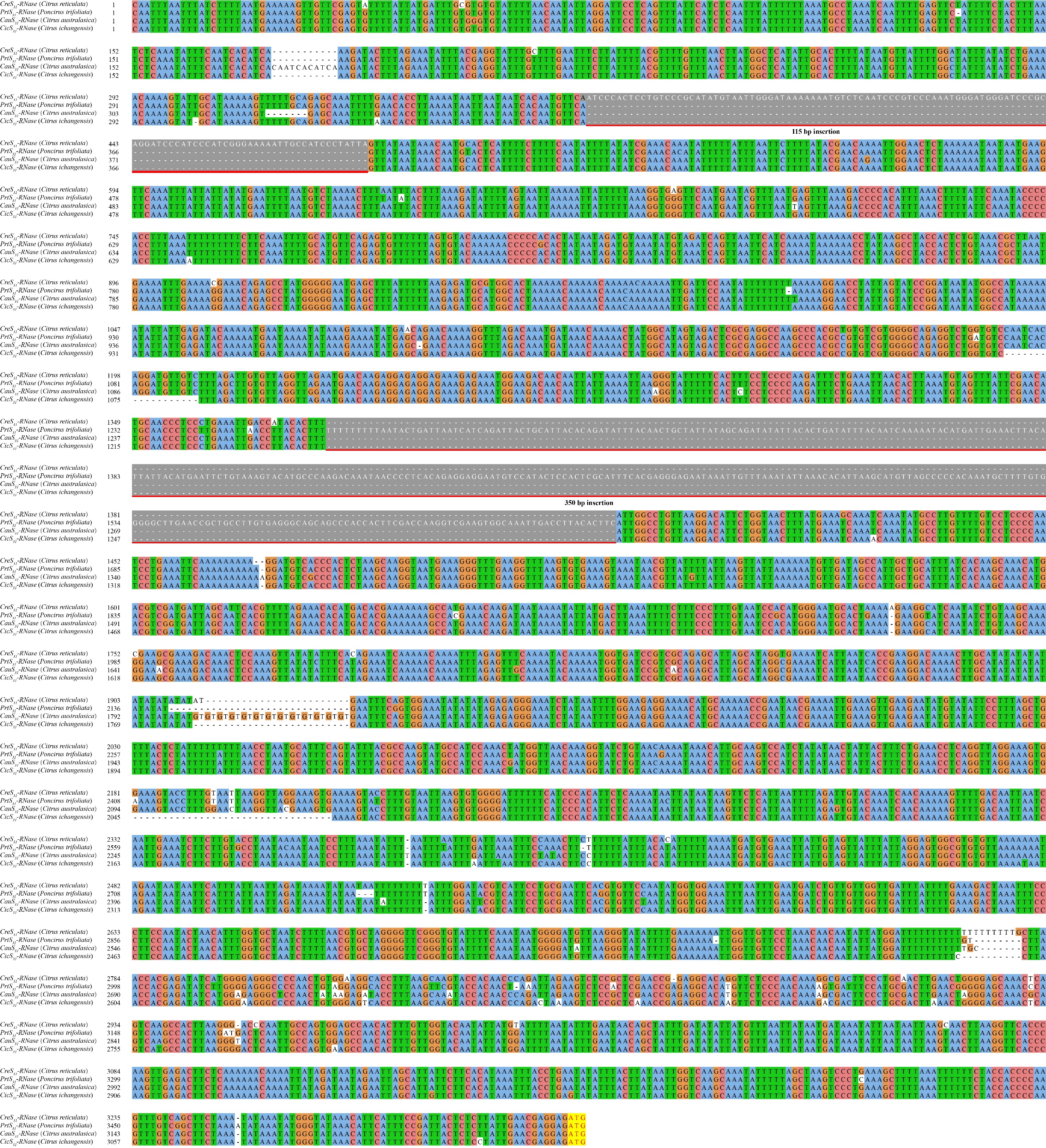
**

**Supplementary Figure 11. Nucleotide sequences alignment of *S_31_-RNase* promoter regions from SC *P. trifoliata*, SI *C. australasia*, SI** ***C. ichangensis*, and SC *C. reticulata*.**

The 115-bp and 350-bp sequences were inserted in the promoter region of the *CreS_31_-RNase* (*C. reticulata*) and *PtrS_31_-RNase* (*P. trifoliata*) allele, respectively. It is absent in *C. australasia* and *C. ichangensis*.

**
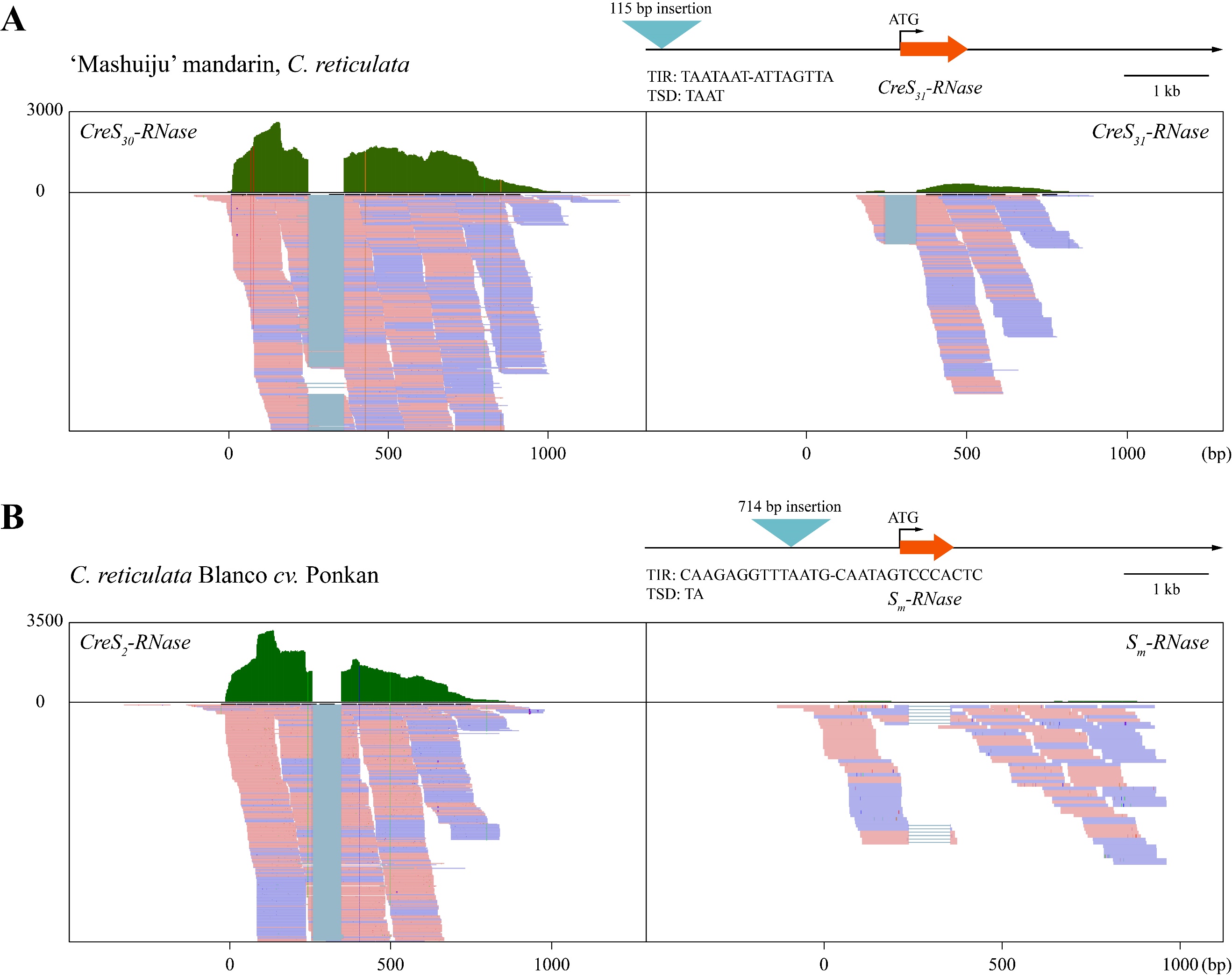
**

**Supplementary Figure 12. Expression of *S-RNase* in the style tissues of SC mandarins.**

**A:** Sequence read clusters from the *CreS_30_-RNase* and *CreS_31_-RNase* alleles. The sequence read clusters are from the RNA-Seq data generated from the styles of ‘mashuiju’ mandarin (*C. reticulata*) and are shown in the Integrative Genomics Viewer. There were significantly more reads mapped to the *CreS_30_-RNase* than to the *CreS_31_-RNase* in the styles of ‘mashuiju’ mandarin (*C. reticulata*). **B:** Sequence read clusters from the *CreS_2_-RNase* and *S_m_-RNase* alleles. The sequence read clusters are from the RNA-Seq data generated from the styles of ‘Ponkan’ (*C. reticulata* Blanco *cv.* Ponkan) and are shown in the Integrative Genomics Viewer. There were significantly more reads mapped to *CreS_2_-RNase* than to *S_m_-RNase* in the styles of ‘Ponkan’. The green bars depict the number of reads mapped to the *S-RNase* sequences, which include the 500-bp 5′-flanking regions, exons, introns and 500-bp 3′-flanking regions). A partial alignment of the RNA mapping data is shown (below). Pink and blue represent the sequences of the different strands.

**
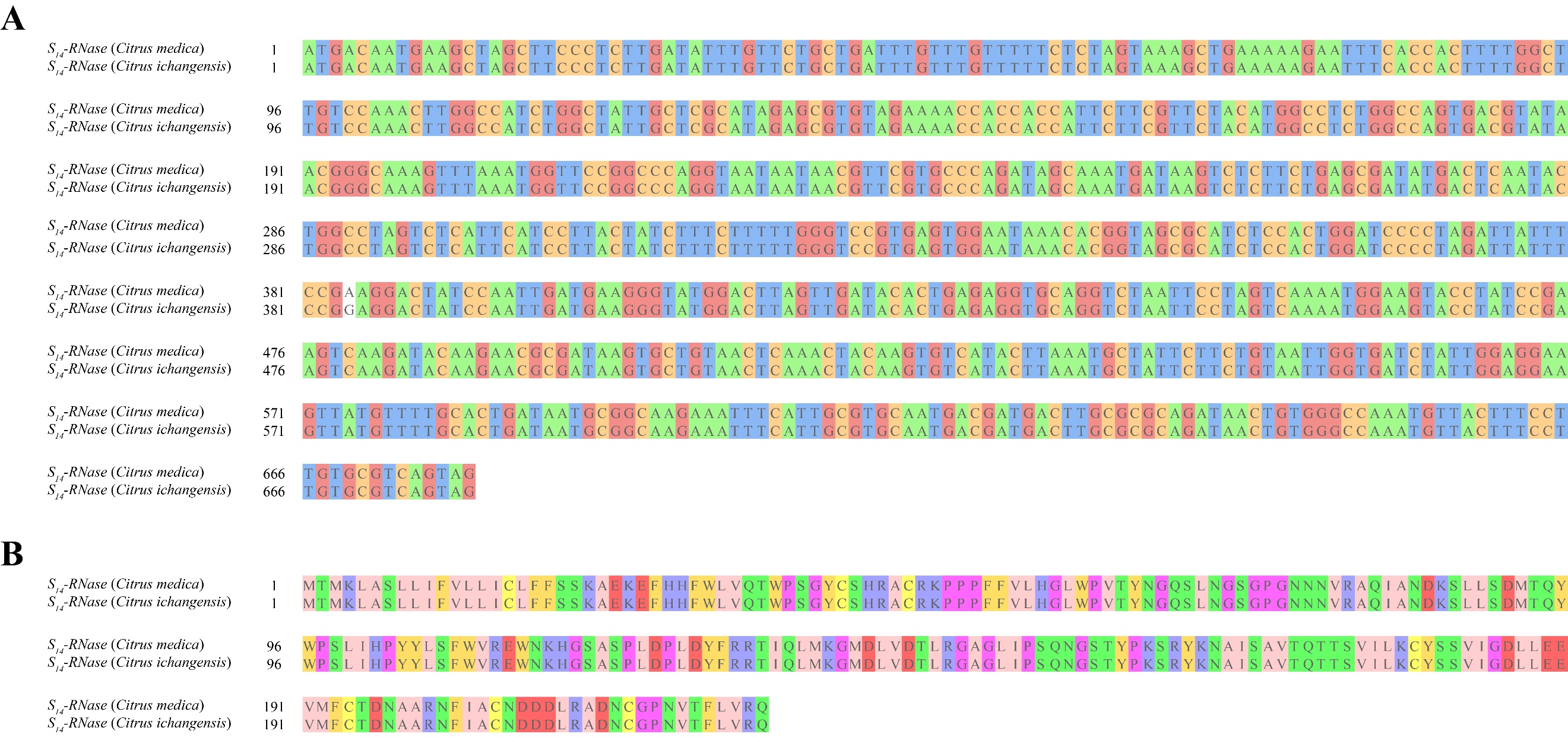
**

**Supplementary Figure 13. Nucleotide and amino acid sequence alignment of *S_14_-RNase* in different intergeneric and interspecific of citrus.**

**A：**CDS sequences of *CmeS_14_-RNase* (*C. medica*) and *CicS_14_-RNase* (*C. ichangensis*) alleles. **B：**Amino acid sequences of *S_14_*-RNases. The nucleotide sequence identities above 80% among *S_14_-RNase* are indicated with shaded boxes. The same color indicates 100% conservation. White indicates SNPs that induce non-synonymous mutations.

**
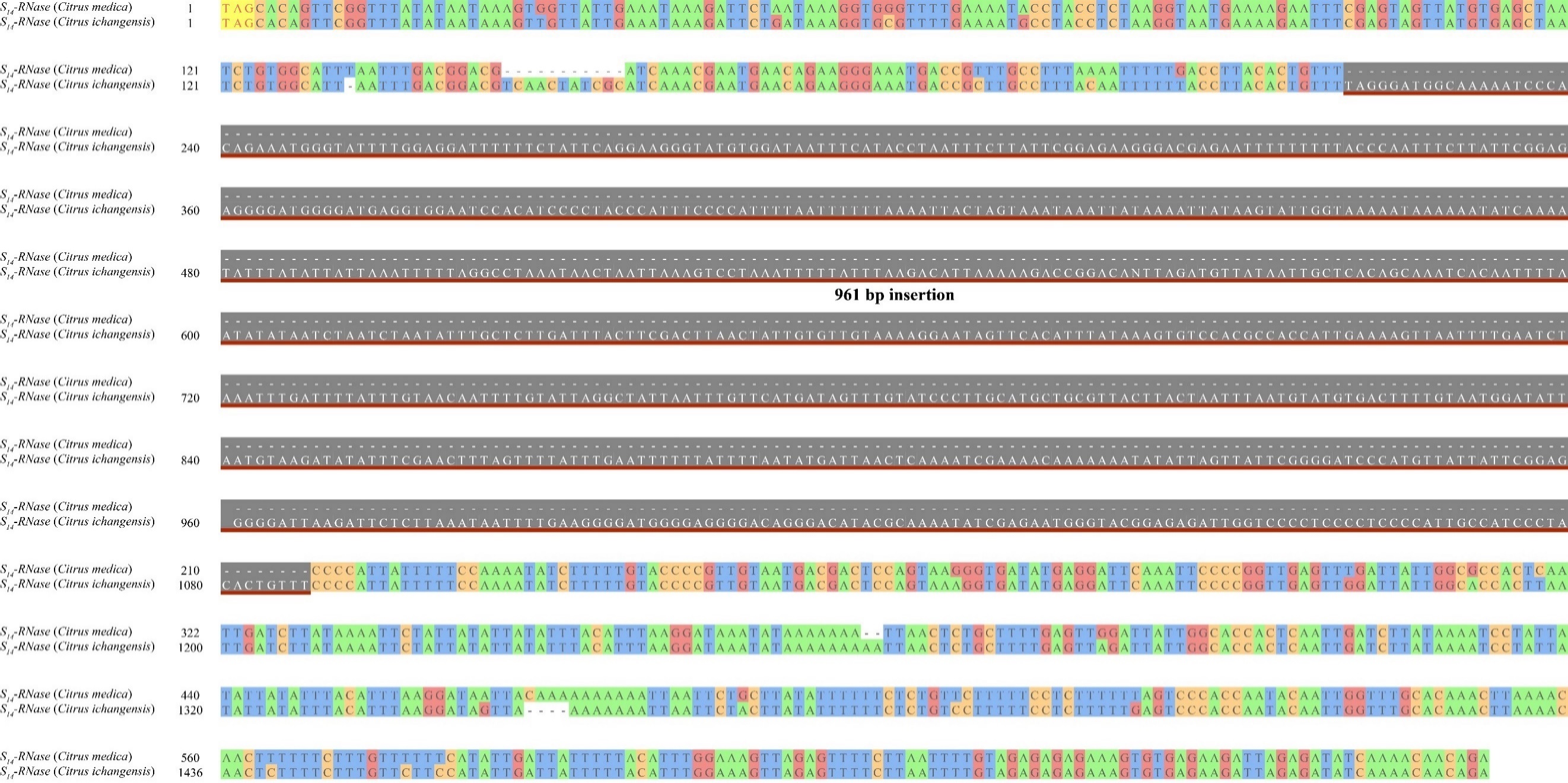
**

**Supplementary Figure 14. Nucleotide sequence alignment of the downstream region of the *S_14_-RNase* allele from SI** ***C. ichangensis* and SC *C. medica*.**

Compared with *C. ichangensis*, a 961 bp miniature inverted-repeat transposable element (MITE) was inserted in the downstream region of the *CmeS_14_-RNase* (*C. medica*) allele.


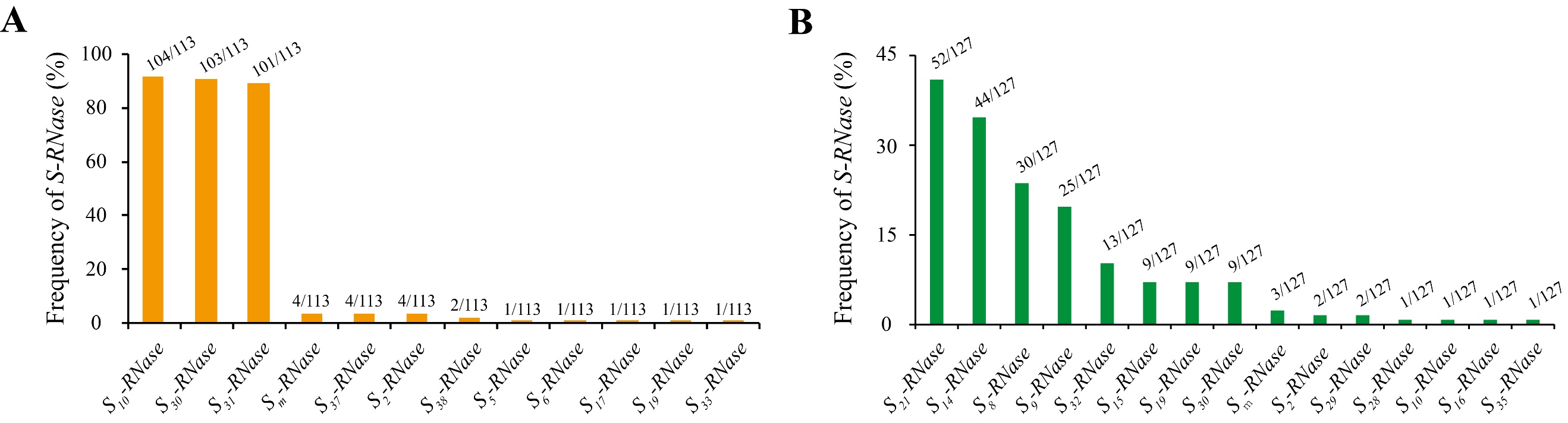


**Supplementary Figure 15. Analysis of the *S*-haplotype of *P. trifoliata* and *C. medica* accessions.**

**A:** Frequency of particular *S-RNase* genes in 113 *P. trifoliata* accessions. These accessions were collected from the preservation germplasm garden for *P. trifoliata* (Danjiangkou, Shiyan city, Hubei province), Institute of Horticultural Research of the Academy of Agricultural Science of Hunan (Changsha, Hunan province), Citrus Research Institute of Huazhong Agrucultural University (Wuhan, Hubei province), and NCBI data. Twelve *S-RNase* alleles were identified in all *P. trifoliata* accessions. The *S_31_-RNase* allele is present at the third highest frequency at 89.38% (101/113). *S_37_-RNase* and *S_38_-RNase* alleles can be found in the GenBank data libraries under accession numbers (OR359662 and OR359663). **B:** Frequency of *S-RNase* in 127 *C. medica* accessions that were collected from Tibet province. The *S*-haplotypes of these accessions were assigned using PCR and leaf DNA with *S-RNase* (*S_1_-RNase* to *S_35_-RNase*) specific primers (**Supplementary Table 17, Supplementary Table 18**).

**Note:** In fact, there are two routes for the loss of SI in *P. trifoliata*. One is the MITE insertion in the promoter of *PtrS_31_-RNase* that we reported in this study. In the other case, two haplotypes (*S_10_* and *S_30_*) recombine to form a new super *S*-locus (data not shown). Therefore, most of the *P. trifoliata* accessions contains three genotypes (*S_10_S_30_S_31_*), because *S_10_* and *S_30_* haplotypes are linked together, as we have demonstrated this using the *C. clementina* (SI) × *P. trifoliata* (SC) hybrid population, and the super haplotype containing *PtrS_10_-RNase* and *PtrS_30_-RNase* are also SC (unpublished). In addition, the expression levels of *PtrS_10_-RNase* and *PtrS_30_-RNase* alleles were not inhibited.

In the 127 *C. medica* accessions examined, the frequency of SC *CmeS_14_-RNase* and *CmeS_21_-RNase* genotypes were high. As we identified MITE insertions in the 3’-flanking and promoter regions of *CmeS_14_-RNase* and *CmeS_21_-RNase* alleles respectively, this high frequency could be attributed to the SC phenotype caused by the MITE insertion.


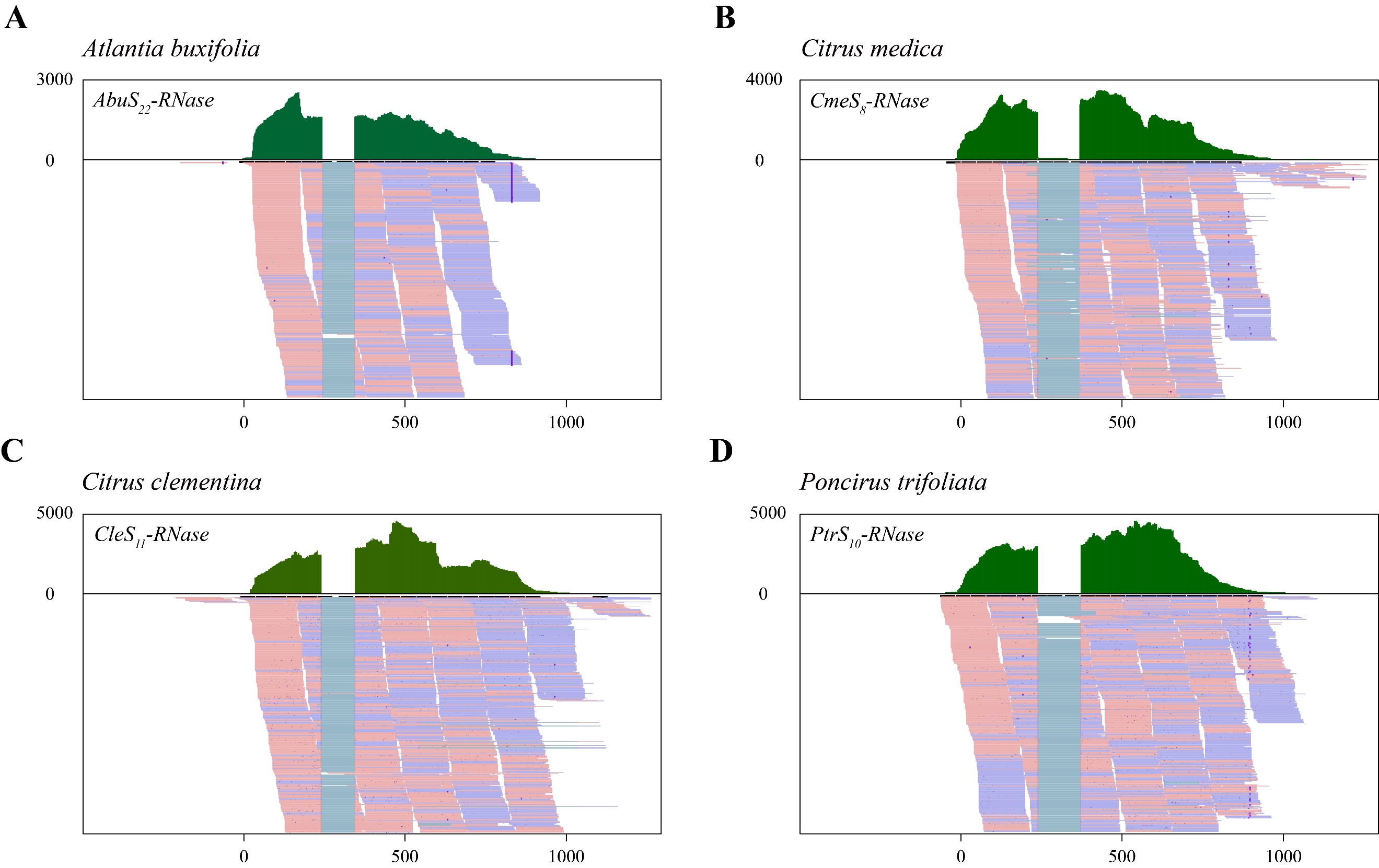


**Supplementary Figure 16. Expression of *S-RNase* in the style tissues in** **different intergeneric and interspecific of citrus.**

Sequence read clusters from the *AbuS_22_-RNase* (**A:** *A. buxifolia*), *CmeS_8_-RNase* (**B:** *C. medica*), *CleS_11_-RNase* (**C:** *C. clementina*), and *PtrS_10_-RNase* (**D:** *P. trifoliata*) alleles. The sequence read clusters are from the RNA-Seq data generated from the styles of different intergeneric and interspecific of citrus, which are shown in the Integrative Genomics Viewer. The green bars depict the number of reads mapped to the *S-RNase* sequences, which include the 500-bp 5′-flanking regions, exons, introns and 500-bp 3′-flanking regions. A partial alignment of the RNA mapping data is shown (below). Pink and blue represent the sequences of the different strands.

**
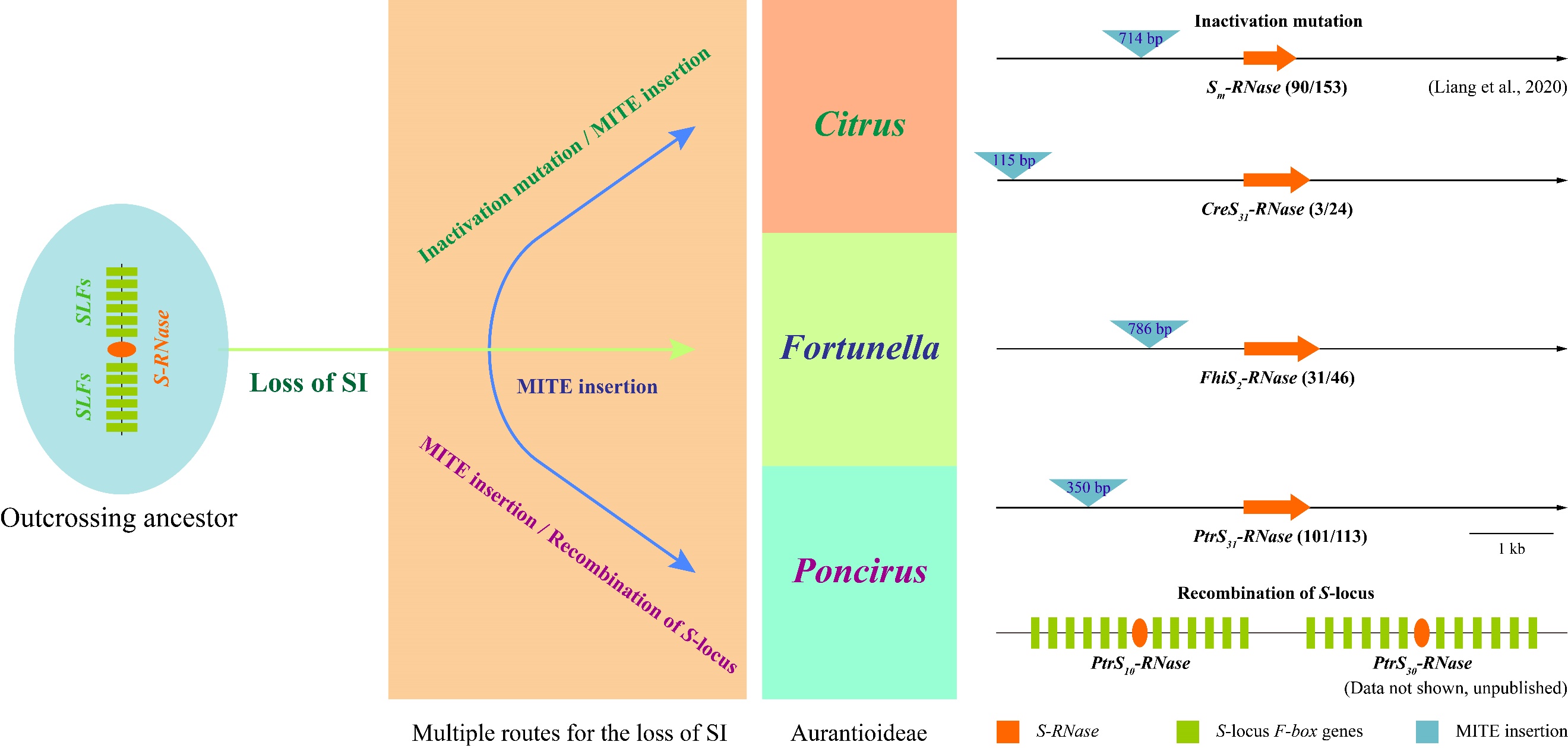
**

**Supplementary Figure 17.** **Multiple routes for the loss of SI in the three major genera (*Citrus*, *Fortunella*, *Poncirus*) of the Aurantioideae.**

We show a cartoon of the *S*-locus, with one *S-RNase* (red ellipse) and 14 *SLFs* (green rectangles) by outcrossing ancestor to indicate the status of its *S*-locus. Two routes for the loss of SI have been identified in *Citrus* (upper), one of which is the reported inactivation mutation of *S* allele, *S_m_-RNase*, which is found in 90 out of 153 of *Citrus* accessions ^3^. Another route is a new SC *S* allele, *CreS_31_-RNase*, whose promoter contains a MITE insertion, resulting in inhibition of its expression (**Supplementary Fig. 12A**). Among the 24 cultivated mandarin accessions, 3 contained this *S* allele (**Supplementary Table 19**). At present, only one route for the loss of SI has been found in *Fortunella* (middle), namely MITE-mediated SI loss reported in this study (*FhiS_2_-RNase*). Among 46 *Fortunella* accessions, 31 contained this *S* allele (**Fig. 2C; Supplementary Table 4**). There are two routes for the loss of SI in *Poncirus* (bottom). One is the MITE insertion in the promoter of *PtrS_31_-RNase* that we reported in this study (**Fig. 6B**). Among the 113 *Poncirus* accessions, 101 contained this *S* allele (**Supplementary Fig. 15**). In the other case, two haplotypes (*PtrS_10_* and *PtrS_30_*) recombine to form a new super *S*-locus (*PtrS_10_*-*S_30_*-locus, data not shown). Therefore, most of the *P. trifoliata* accessions contains three genotypes (*S_10_S_30_S_31_*), because *PtrS_10_* and *PtrS_30_* haplotypes are linked together. In addition, the expression levels of *PtrS_10_-RNase* and *PtrS_30_-RNase* alleles were not inhibited. The loss of SI caused by multiple routes among genera of citrus, resulting in the generation of nonfunctional SC haplotypes, which were associated with insertion of MITEs near *S-RNase*. We propose that these SC haplotypes were not derived from their ancestors, but arose independently after species differentiation and were then fixed in the population, which was the result of coevolution among the different citrus genera and species. Orange blocks represent *S-RNase* alleles, cyan represents MITE transposons.

**Supplementary Tables**

**Supplementary Table 1.** **Identification of self-incompatibility/compatibility in a *F. hindsii* cross-pollination population**

| **No.** | **Accession name** | **Self-incompatibility/compatibility** | | **Genotype** | | **No.** | **Accession name** | **Self-incompatibility/compatibility** | | **Genotype** | |
| --- | --- | --- | --- | --- | --- | --- | --- | --- | --- | --- | --- |
|  |  | **2019** | **2020** |  |  |  |  | **2019** | **2020** |  |  |
| **1** | PD2-006 |  | SI | *S_19_* | *S_29_* | **94** | PD2-321 | SC |  | *S_2_* | *S_19_* |
| **2** | PD2-012 |  | SC | *S_2_* | *S_19_* | **95** | PD2-325 | SC |  | *S_2_* | *S_8_* |
| **3** | PD2-013 |  | SC | *S_2_* | *S_8_* | **96** | PD2-326 | —— | SC | *S_2_* | *S_8_* |
| **4** | PD2-021 | —— | SC | *S_2_* | *S_19_* | **97** | PD2-329 | SI |  | *S_8_* | *S_29_* |
| **5** | PD2-024 |  | SC | *S_2_* | *S_8_* | **98** | PD2-331 | —— | SC | *S_2_* | *S_19_* |
| **6** | PD2-026 | SI | SI | *S_19_* | *S_29_* | **99** | PD2-335 | SI | SI | *S_8_* | *S_29_* |
| **7** | PD2-028 |  | SC | *S_2_* | *S_8_* | **100** | PD2-337 |  | SC | *S_2_* | *S_8_* |
| **8** | PD2-030 | SI | SI | *S_19_* | *S_29_* | **101** | PD2-345 |  | SI | *S_19_* | *S_29_* |
| **9** | PD2-038 | SI |  | *S_8_* | *S_29_* | **102** | PD2-354 | —— | SC | *S_2_* | *S_8_* |
| **10** | PD2-041 | SC | —— | *S_2_* | *S_19_* | **103** | PD2-355 |  | SC | *S_2_* | *S_8_* |
| **11** | PD2-045 | SI | SI | *S_19_* | *S_29_* | **104** | PD2-356 | SC | —— | *S_2_* | *S_8_* |
| **12** | PD2-046 | SI | —— | *S_19_* | *S_29_* | **105** | PD2-360 |  | SC | *S_2_* | *S_19_* |
| **13** | PD2-052 |  | SI | *S_8_* | *S_29_* | **106** | PD2-382 | —— | SI | *S_8_* | *S_29_* |
| **14** | PD2-054 | SI |  | *S_8_* | *S_29_* | **107** | PD2-384 |  | SI | *S_8_* | *S_29_* |
| **15** | PD2-059 | SI | SI | *S_8_* | *S_29_* | **108** | PD2-385 |  | SC | *S_2_* | *S_8_* |
| **16** | PD2-064 | SC |  | *S_2_* | *S_19_* | **109** | PD2-390 | SI | SI | *S_8_* | *S_29_* |
| **17** | PD2-067 |  | SI | *S_8_* | *S_29_* | **110** | PD2-395 | —— | SC | *S_2_* | *S_8_* |
| **18** | PD2-070 | —— | SC | *S_2_* | *S_19_* | **111** | PD2-396 |  | SC | *S_2_* | *S_8_* |
| **19** | PD2-075 | SC | SC | *S_2_* | *S_19_* | **112** | PD2-398 |  | SC | *S_2_* | *S_8_* |
| **20** | PD2-076 | SC | SC | *S_2_* | *S_19_* | **113** | PD2-400 |  | SI | *S_19_* | *S_29_* |
| **21** | PD2-079 | SC | SC | *S_2_* | *S_19_* | **114** | PD2-402 | —— | SC | *S_2_* | *S_8_* |
| **22** | PD2-080 | SI | —— | *S_19_* | *S_29_* | **115** | PD2-405 | SC |  | *S_2_* | *S_19_* |
| **23** | PD2-083 | SC |  | *S_2_* | *S_8_* | **116** | PD2-411 | SI | —— | *S_8_* | *S_29_* |
| **24** | PD2-086 | SI |  | *S_19_* | *S_29_* | **117** | PD2-415 | SC |  | *S_2_* | *S_19_* |
| **25** | PD2-088 | SC | SC | *S_2_* | *S_19_* | **118** | PD2-416 | —— | SC | *S_2_* | *S_19_* |
| **26** | PD2-094 |  | SI | *S_19_* | *S_29_* | **119** | PD2-418 | —— | SC | *S_2_* | *S_8_* |
| **27** | PD2-096 | SI | —— | *S_19_* | *S_29_* | **120** | PD2-424 | —— | SC | *S_2_* | *S_8_* |
| **28** | PD2-097 | SC |  | *S_2_* | *S_19_* | **121** | PD2-425 | SC |  | *S_2_* | *S_19_* |
| **29** | PD2-101 |  | SI | *S_19_* | *S_29_* | **122** | PD2-426 | SC |  | *S_2_* | *S_19_* |
| **30** | PD2-104 | SC |  | *S_2_* | *S_8_* | **123** | PD2-427 | SC |  | *S_2_* | *S_8_* |
| **31** | PD2-107 | SI | SI | *S_19_* | *S_29_* | **124** | PD2-428 | —— | SC | *S_2_* | *S_19_* |
| **32** | PD2-112 |  | SC | *S_2_* | *S_8_* | **125** | PD2-432 |  | SC | *S_2_* | *S_19_* |
| **33** | PD2-113 | SI | —— | *S_19_* | *S_29_* | **126** | PD2-434 |  | SI | *S_8_* | *S_29_* |
| **34** | PD2-114 |  | SI | *S_8_* | *S_29_* | **127** | PD2-436 | SI | SI | *S_8_* | *S_29_* |
| **35** | PD2-120 | SI | SI | *S_8_* | *S_29_* | **128** | PD2-437 | SI |  | *S_19_* | *S_29_* |
| **36** | PD2-123 |  | SI | *S_8_* | *S_29_* | **129** | PD2-438 | SI | SI | *S_8_* | *S_29_* |
| **37** | PD2-128 |  | SC | *S_2_* | *S_8_* | **130** | PD2-440 |  | SC | *S_2_* | *S_8_* |
| **38** | PD2-132 | SC | SC | *S_2_* | *S_8_* | **131** | PD2-443 | —— | SC | *S_2_* | *S_19_* |
| **39** | PD2-135 | SI | —— | *S_19_* | *S_29_* | **132** | PD2-446 | SC | —— | *S_2_* | *S_8_* |
| **40** | PD2-137 |  | SC | *S_2_* | *S_19_* | **133** | PD2-450 | SC | SC | *S_2_* | *S_19_* |
| **41** | PD2-138 |  | SI | *S_19_* | *S_29_* | **134** | PD2-452 | SC |  | *S_2_* | *S_8_* |
| **42** | PD2-142 | SC | SC | *S_2_* | *S_8_* | **135** | PD2-453 | SI |  | *S_19_* | *S_29_* |
| **43** | PD2-154 | SI | SI | *S_8_* | *S_29_* | **136** | PD2-457 | SI |  | *S_19_* | *S_29_* |
| **44** | PD2-155 |  | SC | *S_2_* | *S_8_* | **137** | PD2-458 | —— | SI | *S_8_* | *S_29_* |
| **45** | PD2-158 | SI |  | *S_19_* | *S_29_* | **138** | PD2-460 | SC |  | *S_2_* | *S_8_* |
| **46** | PD2-165 |  | SI | *S_19_* | *S_29_* | **139** | PD2-464 | SI |  | *S_8_* | *S_29_* |
| **47** | PD2-169 | SI | SI | *S_8_* | *S_29_* | **140** | PD2-467 | SC |  | *S_2_* | *S_19_* |
| **48** | PD2-176 |  | SC | *S_2_* | *S_19_* | **141** | PD2-470 | —— | SI | *S_19_* | *S_29_* |
| **49** | PD2-178 | SC | SC | *S_2_* | *S_19_* | **142** | PD2-471 | —— | SI | *S_8_* | *S_29_* |
| **50** | PD2-181 | SC | —— | *S_2_* | *S_19_* | **143** | PD2-472 | —— | SC | *S_2_* | *S_19_* |
| **51** | PD2-184 | SI | SI | *S_8_* | *S_29_* | **144** | PD2-477 | SC |  | *S_2_* | *S_19_* |
| **52** | PD2-187 | SI |  | *S_8_* | *S_29_* | **145** | PD2-480 | SI | SI | *S_19_* | *S_29_* |
| **53** | PD2-195 | SI | SI | *S_19_* | *S_29_* | **146** | PD2-482 | —— | SC | *S_2_* | *S_19_* |
| **54** | PD2-198 | SI | SI | *S_8_* | *S_29_* | **147** | PD2-483 | —— | SC | *S_2_* | *S_19_* |
| **55** | PD2-199 |  | SI | *S_8_* | *S_29_* | **148** | PD2-485 | SC |  | *S_2_* | *S_19_* |
| **56** | PD2-203 |  | SC | *S_2_* | *S_19_* | **149** | PD2-486 | —— | SI | *S_19_* | *S_29_* |
| **57** | PD2-204 | SI | SI | *S_8_* | *S_29_* | **150** | PD2-489 |  | SI | *S_8_* | *S_29_* |
| **58** | PD2-207 | SI | SI | *S_19_* | *S_29_* | **151** | PD2-490 |  | SC | *S_2_* | *S_8_* |
| **59** | PD2-211 | SC | SC | *S_2_* | *S_8_* | **152** | PD2-491 | —— | SC | *S_2_* | *S_19_* |
| **60** | PD2-213 | SC |  | *S_2_* | *S_19_* | **153** | PD2-493 | SI |  | *S_8_* | *S_19_* |
| **61** | PD2-224 |  | SC | *S_2_* | *S_8_* | **154** | PD2-498 | SI |  | *S_19_* | *S_29_* |
| **62** | PD2-225 |  | SI | *S_8_* | *S_29_* | **155** | PD2-500 | —— | SC | *S_2_* | *S_19_* |
| **63** | PD2-228 | SI |  | *S_8_* | *S_29_* | **156** | PD2-501 | SI |  | *S_8_* | *S_29_* |
| **64** | PD2-229 | SC | SC | *S_2_* | *S_8_* | **157** | PD2-506 | SC | SC | *S_2_* | *S_19_* |
| **65** | PD2-230 |  | SI | *S_8_* | *S_19_* | **158** | PD2-510 | SC |  | *S_2_* | *S_8_* |
| **66** | PD2-238 | SC |  | *S_2_* | *S_8_* | **159** | PD2-511 | SC |  | *S_2_* | *S_19_* |
| **67** | PD2-239 | SI | SI | *S_19_* | *S_29_* | **160** | PD2-514 |  | SI | *S_8_* | *S_29_* |
| **68** | PD2-243 | SI | SI | *S_8_* | *S_29_* | **161** | PD2-517 | SI | SI | *S_19_* | *S_29_* |
| **69** | PD2-244 |  | SI | *S_8_* | *S_29_* | **162** | PD2-519 | —— | SC | *S_2_* | *S_19_* |
| **70** | PD2-245 | SI | SI | *S_8_* | *S_29_* | **163** | PD2-522 |  | SC | *S_2_* | *S_19_* |
| **71** | PD2-251 | SI | SI | *S_19_* | *S_29_* | **164** | PD2-527 | SC | —— | *S_2_* | *S_19_* |
| **72** | PD2-254 | SI |  | *S_19_* | *S_29_* | **165** | PD2-533 | SC |  | *S_2_* | *S_19_* |
| **73** | PD2-256 | SC |  | *S_2_* | *S_19_* | **166** | PD2-535 | SI | SI | *S_19_* | *S_29_* |
| **74** | PD2-259 |  | SI | *S_8_* | *S_29_* | **167** | PD2-536 | SC | SC | *S_2_* | *S_8_* |
| **75** | PD2-262 |  | SI | *S_8_* | *S_29_* | **168** | PD2-540 | SI |  | *S_19_* | *S_29_* |
| **76** | PD2-263 | SI |  | *S_19_* | *S_29_* | **169** | PD2-546 | SC | SC | *S_2_* | *S_8_* |
| **77** | PD2-265 |  | SI | *S_19_* | *S_29_* | **170** | PD2-547 | SI | —— | *S_19_* | *S_29_* |
| **78** | PD2-267 |  | SI | *S_19_* | *S_29_* | **171** | PD2-548 | SI |  | *S_19_* | *S_29_* |
| **79** | PD2-268 | SI | SI | *S_8_* | *S_29_* | **172** | PD2-553 | SC |  | *S_2_* | *S_8_* |
| **80** | PD2-270 | SC | SC | *S_2_* | *S_19_* | **173** | PD2-558 | —— | SC | *S_2_* | *S_19_* |
| **81** | PD2-277 | —— | SC | *S_2_* | *S_19_* | **174** | PD2-561 | SC |  | *S_2_* | *S_8_* |
| **82** | PD2-281 |  | SC | *S_2_* | *S_19_* | **175** | PD2-563 | —— | SC | *S_2_* | *S_19_* |
| **83** | PD2-286 |  | SC | *S_2_* | *S_19_* | **176** | PD2-564 | SC |  | *S_2_* | *S_8_* |
| **84** | PD2-290 | SC |  | *S_2_* | *S_19_* | **177** | PD2-565 | SI |  | *S_19_* | *S_29_* |
| **85** | PD2-301 | SI | SI | *S_19_* | *S_29_* | **178** | PD2-566 | SI |  | *S_8_* | *S_29_* |
| **86** | PD2-302 |  | SI | *S_8_* | *S_29_* | **179** | PD2-567 | SI |  | *S_19_* | *S_29_* |
| **87** | PD2-305 | SC | —— | *S_2_* | *S_19_* | **180** | PD2-568 | SC |  | *S_2_* | *S_8_* |
| **88** | PD2-308 |  | SC | *S_2_* | *S_19_* | **181** | PD2-600 | SI |  | *S_19_* | *S_29_* |
| **89** | PD2-309 | SI |  | *S_19_* | *S_29_* | **182** | PD2-602 | SC |  | *S_2_* | *S_19_* |
| **90** | PD2-310 |  | SC | *S_2_* | *S_19_* | **183** | PD2-608 | SC |  | *S_2_* | *S_19_* |
| **91** | PD2-312 | SI |  | *S_19_* | *S_29_* | **184** | PD2-611 | SI |  | *S_19_* | *S_29_* |
| **92** | PD2-313 | SC | SC | *S_2_* | *S_19_* | **185** | PD2-617 | SC |  | *S_2_* | *S_8_* |
| **93** | PD2-314 | SC | —— | *S_2_* | *S_19_* | **186** | PD2-624 | SI |  | *S_8_* | *S_29_* |

**Supplementary Table 2. Statistical analysis of SI/SC phenotypes for the progeny from a PN02 × DB02 hybrid population**

| **Time of assay** | **No. of identified progeny** | **No. of progeny that confirmed SI/SC phenotypes** | **No. of SI plants** | **No. of SC plants** | **Observed ratio** | **Expected ratio** | **χ^2^ value** | ***P* value** |
| --- | --- | --- | --- | --- | --- | --- | --- | --- |
| **2019**  **June-July** | 137 | 111 | 59 | 52 | 59 : 52 | 1 : 1 | 0.44 | 0.51 |
| **2020**  **May-July** | 130 | 116 | 55 | 61 | 55 : 61 | 1 : 1 | 0.31 | 0.58 |

**Supplementary Table 3.** **KASP genotyping analysis of a *F. hindsii* cross-pollination population**

| **No.** | **Accession name** | **mark name** | | | | | | | | | | | | **Phenotype** |
| --- | --- | --- | --- | --- | --- | --- | --- | --- | --- | --- | --- | --- | --- | --- |
|  |  | **K110285** | **K729768** | **K936336** | **K961397** | **K982090** | **K1200030** | **K1247997** | **K1270101** | **K1314434** | **K1512779** | **K1850875** | **K2206344** |  |
| **1** | PD2-006 | CC | GC | AA | AG | GA | CT | CT | CC | TG | GA | AA | GG | SI |
| **2** | PD2-012 | CA | GG | AT | AA | GG | CC | CC | CA | TT | GG | AG | GT | SC |
| **3** | PD2-013 | CA | GG | AT | AA | GG | CC | CC | CA | TT | GG | AG | GT | SC |
| **4** | PD2-021 | CA | GG | AT | AA | GG | CC | CC | CA | TT | GG | AG | GT | SC |
| **5** | PD2-024 | CA | GG | AT | AA | GG | CC | CC | CA | TT | GG | AG | GT | SC |
| **6** | PD2-026 | CC | GC | AA | AG | GA | CT | CT | CC | TG | GA | AA | GG | SI |
| **7** | PD2-028 | CA | GG | AT | AA | GG | CC | CC | CA | TG | GA | AA | GG | SC |
| **8** | PD2-030 | CC | GC | AA | AG | GA | CT | CT | CC | TG | GA | AA | GG | SI |
| **9** | PD2-038 | CC | GC | AA | AG | GA | CT | CT | CC | TG | GA | AA | GG | SI |
| **10** | PD2-041 | CA | GG | AT | AA | GG | CC | CC | CA | TT | GG | AG | GT | SC |
| **11** | PD2-045 | CC | GC | AA | AG | GA | CT | CT | CC | TG | GA | AA | GG | SI |
| **12** | PD2-046 | CC | GC | AA | AG | GA | CT | CT | CC | TG | GA | AA | GG | SI |
| **13** | PD2-052 | CA | GG | AA | AG | GA | CT | CT | CC | TG | GA | AA | GG | SI |
| **14** | PD2-054 | CC | GC | AA | AG | GA | CT | CT | CC | TG | GA | AA | GG | SI |
| **15** | PD2-059 | CC | GC | AA | AG | GA | CT | CT | CC | TG | GA | AG | GT | SI |
| **16** | PD2-064 | CA | GG | AT | AA | GG | CC | CC | CA | TT | GG | AG | GT | SC |
| **17** | PD2-067 | CC | GC | AA | AG | GA | CT | CT | CC | TG | GA | AA | GG | SI |
| **18** | PD2-070 | CA | GG | AT | AA | GG | CC | CC | CA | TT | GG | AG | GT | SC |
| **19** | PD2-075 | CA | GG | AT | AA | GG | CC | CC | CA | TT | GG | AG | GT | SC |
| **20** | PD2-076 | CA | GG | AT | AA | GG | CC | CC | CA | TT | GG | AG | GT | SC |
| **21** | PD2-079 | CA | GG | AT | AA | GG | CC | CC | CA | TT | GG | AG | GT | SC |
| **22** | PD2-080 | CC | GC | AA | AG | GA | CT | CT | CC | TG | GA | AA | GG | SI |
| **23** | PD2-083 | CA | GG | AT | AA | GG | CC | CC | CA | TT | GG | AG | GT | SC |
| **24** | PD2-086 | CA | GC | AA | AG | GA | CT | CT | CC | TG | GA | AA | GG | SI |
| **25** | PD2-088 | CA | GG | AT | AA | GG | CC | CC | CA | TT | GG | AG | GT | SC |
| **26** | PD2-094 | CC | GC | AA | AG | GA | CT | CT | CC | TG | GA | AA | GG | SI |
| **27** | PD2-096 | CC | GC | AA | AG | GA | CT | CT | CC | TG | GA | AA | GG | SI |
| **28** | PD2-097 | CC | GC | AT | AA | GG | CC | CC | CA | TT | GG | AG | GT | SC |
| **29** | PD2-101 | CC | GC | AA | AG | GA | CT | CT | CC | TG | GA | AA | GG | SI |
| **30** | PD2-104 | CA | GG | AT | AA | GG | CC | CC | CA | TT | GA | AA | GG | SC |
| **31** | PD2-107 | CA | GG | AA | AG | GA | CT | CT | CC | TG | GA | AA | GG | SI |
| **32** | PD2-112 | CA | GG | AT | AA | GG | CC | CC | CA | TT | GG | AG | GT | SC |
| **33** | PD2-113 | CC | GC | AA | AG | GA | CT | CT | CC | TG | GA | AA | GG | SI |
| **34** | PD2-114 | CC | GC | AA | AG | GA | CT | CT | CC | TG | GA | AA | GG | SI |
| **35** | PD2-120 | CC | GC | AA | AG | GA | CT | CT | CC | TG | GA | AA | GG | SI |
| **36** | PD2-123 | CC | GC | AA | AG | GA | CT | CT | CC | TG | GA | AA | GG | SI |
| **37** | PD2-128 | CA | GG | AT | AA | GG | CC | CC | CA | TT | GG | AG | GT | SC |
| **38** | PD2-132 | CA | GG | AT | AA | GG | CC | CC | CA | TT | GG | AG | GT | SC |
| **39** | PD2-135 | CC | GC | AA | AG | GA | CT | CT | CC | TG | GA | AA | GG | SI |
| **40** | PD2-137 | CA | GG | AT | AA | GG | CC | CC | CA | TT | GG | AG | GT | SC |
| **41** | PD2-138 | CC | GC | AA | AG | GA | CT | CT | CC | TG | GA | AA | GG | SI |
| **42** | PD2-142 | CC | GC | AT | AA | GG | CC | CC | CA | TT | GG | AG | GT | SC |
| **43** | PD2-154 | CC | GC | AA | AG | GA | CT | CT | CC | TG | GA | AA | GG | SI |
| **44** | PD2-155 | CA | GG | AT | AA | GG | CC | CC | CA | TT | GG | AG | GT | SC |
| **45** | PD2-158 | CC | GC | AA | AG | GA | CT | CT | CC | TT | GG | AG | GT | SI |
| **46** | PD2-165 | CC | GC | AA | AG | GA | CT | CT | CC | TG | GA | AA | GG | SI |
| **47** | PD2-169 | CC | GC | AA | AG | GA | CT | CT | CC | TG | GA | AA | GG | SI |
| **48** | PD2-176 | CA | GG | AT | AA | GG | CC | CC | CA | TT | GG | AG | GT | SC |
| **49** | PD2-178 | CA | GG | AT | AA | GG | CC | CC | CA | TT | GG | AG | GT | SC |
| **50** | PD2-181 | CA | GG | AT | AA | GG | CC | CC | CA | TT | GG | AG | GT | SC |
| **51** | PD2-184 | CC | GC | AA | AG | GA | CT | CT | CC | TG | GA | AA | GG | SI |
| **52** | PD2-187 | CC | GC | AA | AG | GA | CT | CT | CC | TG | GA | AA | GG | SI |
| **53** | PD2-195 | CC | GC | AA | AG | GA | CT | CT | CC | TG | GA | AA | GG | SI |
| **54** | PD2-198 | CC | GC | AA | AG | GA | CT | CT | CC | TG | GA | AA | GG | SI |
| **55** | PD2-199 | CC | GC | AA | AG | GA | CT | CT | CC | TG | GA | AA | GG | SI |
| **56** | PD2-203 | CA | GG | AT | AA | GG | CC | CC | CA | TT | GG | AG | GT | SC |
| **57** | PD2-204 | CC | GC | AA | AG | GA | CT | CT | CC | TG | GA | AA | GG | SI |
| **58** | PD2-207 | CC | GC | AA | AG | GA | CT | CT | CC | TG | GA | AA | GG | SI |
| **59** | PD2-211 | CA | GG | AT | AA | GG | CC | CT | CC | TG | GA | AA | GG | SC |
| **60** | PD2-213 | CA | GG | AT | AA | GG | CC | CC | CA | TT | GG | AG | GT | SC |
| **61** | PD2-224 | CA | GG | AT | AA | GG | CC | CC | CA | TT | GG | AG | GT | SC |
| **62** | PD2-225 | CC | GC | AA | AG | GA | CT | CT | CC | TG | GA | AA | GG | SI |
| **63** | PD2-228 | CC | GC | AA | AG | GA | CT | CT | CC | TG | GA | AA | GG | SI |
| **64** | PD2-229 | CA | GG | AT | AA | GG | CC | CC | CA | TT | GG | AG | GT | SC |
| **65** | PD2-230 | CC | GC | AA | AG | GA | CT | CT | CC | TG | GA | AA | GG | SI |
| **66** | PD2-238 | CA | GG | AT | AA | GG | CC | CC | CA | TT | GG | AG | GT | SC |
| **67** | PD2-239 | CC | GC | AA | AG | GA | CT | CT | CC | TG | GA | AA | GG | SI |
| **68** | PD2-243 | CC | GC | AA | AG | GA | CT | CT | CC | TG | GA | AA | GG | SI |
| **69** | PD2-244 | CC | GC | AA | AG | GA | CT | CT | CC | TG | GA | AA | GG | SI |
| **70** | PD2-245 | CC | GC | AA | AG | GA | CT | CT | CC | TG | GA | AA | GG | SI |
| **71** | PD2-251 | CC | GC | AA | AG | GA | CT | CT | CC | TG | GA | AA | GG | SI |
| **72** | PD2-254 | CC | GC | AA | AG | GA | CT | CT | CC | TG | GA | AA | GG | SI |
| **73** | PD2-256 | CA | GG | AT | AA | GG | CC | CC | CA | TT | GG | AG | GT | SC |
| **74** | PD2-259 | CC | GC | AA | AG | GA | CT | CT | CC | TG | GA | AA | GG | SI |
| **75** | PD2-262 | CC | GC | AA | AG | GA | CT | CT | CC | TG | GA | AA | GG | SI |
| **76** | PD2-263 | CC | GC | AA | AG | GA | CT | CT | CC | TG | GA | AA | GG | SI |
| **77** | PD2-265 | CC | GC | AA | AG | GA | CT | CT | CC | TG | GA | AA | GG | SI |
| **78** | PD2-267 | CC | GC | AA | AG | GA | CT | CT | CC | TG | GA | AA | GG | SI |
| **79** | PD2-268 | CC | GC | AA | AG | GA | CT | CT | CC | TG | GG | AG | GT | SI |
| **80** | PD2-270 | CC | GC | AA | AG | GG | CC | CC | CA | TT | GG | AG | GT | SC |
| **81** | PD2-277 | CA | GG | AT | AA | GG | CC | CC | CA | TT | GG | AG | GT | SC |
| **82** | PD2-281 | CA | GG | AT | AA | GG | CC | CC | CA | TT | GG | AG | GT | SC |
| **83** | PD2-286 | CA | GG | AT | AA | GG | CC | CC | CA | TT | GG | AG | GT | SC |
| **84** | PD2-290 | CA | GG | AT | AA | GG | CC | CC | CA | TT | GG | AG | GT | SC |
| **85** | PD2-301 | CA | GG | AA | AG | GA | CT | CT | CC | TG | GA | AA | GG | SI |
| **86** | PD2-302 | CC | GC | AA | AG | GA | CT | CT | CC | TG | GA | AA | GG | SI |
| **87** | PD2-305 | CA | GG | AT | AA | GG | CC | CC | CA | TT | GG | AG | GT | SC |
| **88** | PD2-308 | CA | GG | AT | AA | GG | CC | CC | CA | TT | GA | AA | GG | SC |
| **89** | PD2-309 | CC | GC | AA | AG | GA | CT | CT | CC | TG | GA | AA | GG | SI |
| **90** | PD2-310 | CA | GG | AT | AA | GG | CC | CC | CA | TT | GG | AG | GT | SC |
| **91** | PD2-312 | CC | GC | AA | AG | GA | CT | CT | CC | TG | GA | AA | GG | SI |
| **92** | PD2-313 | CC | GC | AA | AG | GA | CT | CT | CC | TG | GA | AA | GG | SI |
| **93** | PD2-314 | CA | GG | AT | AA | GG | CC | CC | CA | TT | GG | AG | GT | SC |
| **94** | PD2-321 | CA | GG | AT | AA | GG | CC | CC | CA | TT | GG | AG | GT | SC |
| **95** | PD2-325 | CA | GG | AT | AA | GG | CC | CC | CA | TT | GG | AG | GT | SC |
| **96** | PD2-326 | CA | GG | AT | AA | GG | CC | CC | CA | TT | GG | AG | GT | SC |
| **97** | PD2-329 | CC | GC | AA | AG | GA | CT | CT | CC | TG | GA | AA | GG | SI |
| **98** | PD2-331 | CA | GG | AT | AA | GG | CC | CC | CA | TT | GG | AG | GT | SC |
| **99** | PD2-335 | CC | GC | AA | AG | GA | CT | CT | CC | TG | GA | AA | GG | SI |
| **100** | PD2-337 | CA | GG | AT | AA | GG | CC | CC | CA | TT | GG | AG | GT | SC |
| **101** | PD2-345 | CC | GC | AA | AG | GA | CT | CT | CC | TG | GA | AA | GG | SI |
| **102** | PD2-354 | CA | GG | AT | AA | GG | CC | CC | CA | TT | GG | AG | GT | SC |
| **103** | PD2-355 | CA | GG | AT | AA | GG | CC | CC | CA | TT | GG | AG | GT | SC |
| **104** | PD2-356 | CA | GG | AT | AA | GG | CC | CC | CA | TT | GG | AG | GT | SC |
| **105** | PD2-360 | CA | GG | AT | AA | GG | CC | CC | CA | TT | GG | AG | GT | SC |
| **106** | PD2-382 | CC | GC | AA | AG | GA | CT | CT | CC | TG | GA | AA | GG | SI |
| **107** | PD2-384 | CC | GC | AA | AG | GA | CT | CT | CC | TG | GA | AA | GG | SI |
| **108** | PD2-385 | CA | GG | AT | AA | GG | CC | CC | CA | TT | GG | AG | GT | SC |
| **109** | PD2-390 | CC | GC | AA | AG | GA | CT | CT | CC | TG | GA | AA | GG | SI |
| **110** | PD2-395 | CA | GG | AT | AA | GG | CC | CC | CA | TT | GG | AG | GT | SC |
| **111** | PD2-396 | CA | GG | AT | AA | GG | CC | CC | CA | TT | GG | AG | GT | SC |
| **112** | PD2-398 | CA | GG | AT | AA | GG | CC | CC | CA | TT | GG | AG | GT | SC |
| **113** | PD2-400 | CC | GC | AA | AG | GA | CT | CT | CC | TG | GA | AA | GG | SI |
| **114** | PD2-402 | CA | GG | AT | AA | GG | CC | CC | CA | TT | GG | AG | GT | SC |
| **115** | PD2-405 | CA | GG | AT | AA | GG | CC | CC | CA | TT | GG | AG | GT | SC |
| **116** | PD2-411 | CC | GC | AA | AG | GA | CT | CT | CC | TG | GA | AA | GG | SI |
| **117** | PD2-415 | CA | GG | AT | AA | GG | CC | CC | CA | TT | GG | AG | GT | SC |
| **118** | PD2-416 | CA | GG | AT | AA | GG | CC | CC | CA | TT | GG | AG | GT | SC |
| **119** | PD2-418 | CA | GG | AT | AA | GG | CC | CC | CA | TT | GG | AG | GG | SC |
| **120** | PD2-424 | CA | GG | AT | AA | GG | CC | CC | CA | TT | GG | AG | GT | SC |
| **121** | PD2-425 | CA | GG | AT | AA | GG | CC | CC | CA | TT | GG | AG | GT | SC |
| **122** | PD2-426 | CA | GG | AT | AA | GG | CC | CC | CA | TT | GG | AG | GT | SC |
| **123** | PD2-427 | CA | GG | AT | AA | GG | CC | CC | CA | TT | GG | AG | GT | SC |
| **124** | PD2-428 | CA | GG | AT | AA | GG | CC | CC | CA | TT | GG | AG | GT | SC |
| **125** | PD2-432 | CA | GG | AT | AA | GG | CC | CC | CA | TT | GG | AG | GT | SC |
| **126** | PD2-434 | CC | GC | AA | AG | GA | CT | CT | CC | TG | GA | AA | GG | SI |
| **127** | PD2-436 | CC | GC | AA | AG | GA | CT | CT | CC | TG | GA | AA | GG | SI |
| **128** | PD2-437 | CC | GC | AA | AG | GA | CT | CT | CC | TG | GA | AA | GG | SI |
| **129** | PD2-438 | CC | GC | AA | AG | GA | CT | CT | CC | TG | GA | AA | GG | SI |
| **130** | PD2-440 | CA | GG | AT | AA | GG | CC | CC | CA | TT | GG | AG | GT | SC |
| **131** | PD2-443 | CA | GG | AT | AA | GG | CC | CC | CA | TT | GG | AG | GT | SC |
| **132** | PD2-446 | CA | GG | AT | AA | GG | CC | CC | CA | TT | GG | AG | GT | SC |
| **133** | PD2-450 | CA | GG | AT | AA | GG | CC | CC | CA | TT | GG | AG | GT | SC |
| **134** | PD2-452 | CA | GG | AT | AA | GG | CC | CC | CA | TT | GG | AG | GT | SC |
| **135** | PD2-453 | CC | GC | AA | AG | GA | CT | CT | CC | TG | GA | AA | GG | SI |
| **136** | PD2-457 | CC | GC | AA | AG | GA | CT | CT | CC | TG | GA | AA | GG | SI |
| **137** | PD2-458 | CC | GC | AA | AG | GA | CT | CT | CC | TG | GA | AA | GG | SI |
| **138** | PD2-460 | CA | GG | AT | AA | GG | CC | CC | CA | TT | GG | AG | GT | SC |
| **139** | PD2-464 | CC | GC | AA | AG | GA | CT | CT | CC | TG | GA | AA | GG | SI |
| **140** | PD2-467 | CA | GG | AT | AA | GG | CC | CC | CA | TT | GG | AG | GT | SC |
| **141** | PD2-470 | CC | GC | AA | AG | GA | CT | CT | CC | TG | GA | AA | GG | SI |
| **142** | PD2-471 | CC | GC | AA | AG | GA | CT | CT | CC | TG | GA | AA | GG | SI |
| **143** | PD2-472 | CA | GG | AT | AA | GG | CC | CC | CA | TT | GG | AG | GT | SC |
| **144** | PD2-477 | CA | GG | AT | AA | GG | CC | CC | CA | TT | GG | AG | GT | SC |
| **145** | PD2-480 | CC | GC | AA | AG | GA | CT | CT | CC | TG | GA | AA | GG | SI |
| **146** | PD2-482 | CA | GG | AT | AA | GG | CC | CC | CA | TT | GG | AA | GG | SC |
| **147** | PD2-483 | CA | GG | AT | AA | GG | CC | CC | CA | TT | GG | AG | GT | SC |
| **148** | PD2-485 | CA | GG | AT | AA | GG | CC | CC | CA | TT | GG | AG | GT | SC |
| **149** | PD2-486 | CC | GC | AA | AG | GA | CT | CT | CC | TG | GA | AA | GG | SI |
| **150** | PD2-489 | CC | GC | AA | AG | GA | CT | CT | CC | TG | GA | AA | GG | SI |
| **151** | PD2-490 | CA | GG | AT | AA | GG | CC | CC | CA | TT | GG | AG | GT | SC |
| **152** | PD2-491 | CA | GG | AT | AA | GG | CC | CC | CA | TT | GA | AA | GG | SC |
| **153** | PD2-493 | CC | GC | AA | AG | GA | CT | CT | CC | TG | GA | AA | GG | SI |
| **154** | PD2-498 | CC | GC | AA | AG | GA | CT | CT | CC | TG | GA | AA | GG | SI |
| **155** | PD2-500 | CA | GG | AT | AA | GG | CC | CC | CA | TT | GG | AG | GT | SC |
| **156** | PD2-501 | CC | GC | AA | AG | GA | CT | CT | CC | TG | GA | AA | GG | SI |
| **157** | PD2-506 | CA | GG | AT | AA | GG | CC | CC | CA | TT | GG | AG | GT | SC |
| **158** | PD2-510 | CA | GG | AT | AA | GG | CC | CC | CA | TT | GG | AG | GT | SC |
| **159** | PD2-511 | CA | GG | AT | AA | GG | CC | CC | CA | TT | GG | AG | GT | SC |
| **160** | PD2-514 | CC | GC | AA | AG | GA | CT | CT | CC | TG | GA | AA | GG | SI |
| **161** | PD2-517 | CC | GC | AA | AG | GA | CT | CT | CC | TG | GA | AG | GT | SI |
| **162** | PD2-519 | CA | GG | AT | AA | GG | CC | CC | CA | TT | GG | AG | GT | SC |
| **163** | PD2-522 | CA | GG | AT | AA | GG | CC | CC | CA | TT | GG | AG | GT | SC |
| **164** | PD2-527 | CA | GG | AT | AA | GG | CC | CC | CA | TT | GG | AG | GT | SC |
| **165** | PD2-533 | CA | GG | AT | AA | GG | CC | CC | CA | TT | GG | AG | GT | SC |
| **166** | PD2-535 | CC | GC | AA | AG | GA | CT | CT | CC | TG | GA | AA | GG | SI |
| **167** | PD2-536 | CA | GG | AT | AA | GG | CC | CC | CA | TT | GG | AG | GT | SC |
| **168** | PD2-540 | CC | GC | AA | AG | GA | CT | CT | CC | TG | GA | AA | GG | SI |
| **169** | PD2-546 | CA | GG | AT | AA | GG | CC | CC | CA | TT | GG | AG | GT | SC |
| **170** | PD2-547 | CC | GC | AA | AG | GA | CT | CT | CC | TG | GA | AA | GG | SI |
| **171** | PD2-548 | CC | GC | AA | AG | GA | CT | CT | CC | TG | GA | AA | GG | SI |
| **172** | PD2-553 | CA | GG | AT | AA | GG | CC | CC | CA | TT | GG | AG | GT | SC |
| **173** | PD2-558 | CA | GG | AT | AA | GG | CC | CC | CA | TT | GG | AG | GT | SC |
| **174** | PD2-561 | CA | GG | AT | AA | GG | CC | CC | CA | TT | GG | AG | GT | SC |
| **175** | PD2-563 | CA | GG | AT | AA | GG | CC | CC | CA | TT | GG | AG | GT | SC |
| **176** | PD2-564 | CA | GG | AT | AA | GG | CC | CC | CA | TT | GG | AG | GT | SC |
| **177** | PD2-565 | CC | GC | AA | AG | GA | CT | CT | CC | TG | GA | AA | GG | SI |
| **178** | PD2-566 | CC | GC | AA | AG | GA | CT | CT | CC | TG | GA | AA | GG | SI |
| **179** | PD2-567 | CC | GC | AA | AG | GA | CT | CT | CC | TG | GA | AA | GG | SI |
| **180** | PD2-568 | CA | GG | AT | AA | GG | CC | CC | CA | TT | GG | AG | GT | SC |
| **181** | PD2-600 | CC | GC | AA | AG | GA | CT | CT | CC | TG | GA | AA | GG | SI |
| **182** | PD2-602 | CA | GG | AT | AA | GG | CC | CC | CA | TT | GG | AG | GT | SC |
| **183** | PD2-608 | CA | GG | AT | AA | GG | CC | CC | CA | TT | GG | AG | GT | SC |
| **184** | PD2-611 | CC | GC | AA | AG | GA | CT | CT | CC | TG | GA | AA | GG | SI |
| **185** | PD2-617 | CA | GG | AT | AA | GG | CC | CC | CA | TT | GG | AG | GT | SC |
| **186** | PD2-624 | CC | GC | AA | AG | GA | CT | CT | CC | TG | GA | AA | GG | SI |

**Supplementary Table 4.** **Genotype and SC phenotype identification of 46 *F. hindsii* accessions**

| **NO.** | **Accession Name** | **Genotype** | | **Phenotype** | **Northern latitude** | **East longitude** | **Location** | **SRR ID in Genebank** | **Source** |
| --- | --- | --- | --- | --- | --- | --- | --- | --- | --- |
| **1** | HS03 | *S_2_* | *S_30_* | SC | 25.65 | 114.26964 | Hengshui Town, Chongyi County, Ganzhou City, Jiangxi Province | SRR14761139 | Wang et al., 2022 ^1^ |
| **2** | DYS002-5 | *S_2_* | *S_32_* | SC | 26.40136 | 117.63089 | Fukou Town, Sha County, Sanming City, Fujian Province | SRR14761106 | Wang et al., 2022 ^1^ |
| **3** | FC11 | *S_2_* | *S_29_* | SC | 24.615 | 116.79472 | Xiayang Town, Yongding County, Longyan City, Fujian Province | SRR14761103 | Wang et al., 2022 ^1^ |
| **4** | DYT01-12 | *S_1_* | *S_2_* | SC | 25.08878 | 116.74297 | Dachi Town, Silla District, Longyan City, Fujian Province | SRR14761105 | Wang et al., 2022 ^1^ |
| **5** | QK03 | *S_2_* | *S_32_* | SC | 25.92036 | 113.17767 | Qiaokou Town, Suxian District, Chenzhou City, Hunan Province | SRR14761124 | Wang et al., 2022 ^1^ |
| **6** | HK02 | *S_2_* | *S_3_* | SC | 25.4509 | 114.10228 | Niedu Town, Chongyi County, Ganzhou City, Jiangxi Province | SRR14761140 | Wang et al., 2022 ^1^ |
| **7** | GHS01 | *S_2_* | *S_28_* | SC | 26.80319 | 120.02025 | Guxian Village, Xiapu County, Ningde City, Fujian Province | SRR14761143 | Wang et al., 2022 ^1^ |
| **8** | SK03 | *S_2_* | *S_33_* | SC | # | # | Shuikou Town, Dehua County, Quanzhou City, Fujian Province | SRR14761120 | Wang et al., 2022 ^1^ |
| **9** | GZ01 | *S_1_* | *S_2_* | SC | 25.28208 | 116.94017 | Jiangshan Township, Silla District, Longyan City, Fujian Province | SRR14761141 | Wang et al., 2022 ^1^ |
| **10** | FC01 | *S_2_* | *S_17_* | SC | 24.615 | 116.79472 | Xiayang Town, Yongding County, Longyan City, Fujian Province | SRR14761104 | Wang et al., 2022 ^1^ |
| **11** | SD01 | *S_2_* | *S_2_* | SC | 26.65633 | 119.69428 | Sandu Town, Jiaocheng District, Ningde City, Fujian Province | SRR14761122 | Wang et al., 2022 ^1^ |
| **12** | MP12 | *S_1_* | *S_2_* | SC | 26.13789 | 116.87872 | Songkou Town, Qingliu County, Sanming City, Fujian Province | SRR14761130 | Wang et al., 2022 ^1^ |
| **13** | TX01 | *S_2_* | *S_4_* | SC | # | # | Tingxi Town, Tong’an District, Xiamen City, Fujian Province | SRR14761116 | Wang et al., 2022 ^1^ |
| **14** | LH10 | *S_2_* | *S_4_* | SC | 24.84 | 117.91 | Lianhua Town, Tong’an District, Xiamen City, Fujian Province | SRR14761137 | Wang et al., 2022 ^1^ |
| **15** | WP | *S_2_* | *S_6_* | SC | # | # | Wuping County, Longyan City, Fujian Province | SRR14761112 | Wang et al., 2022 ^1^ |
| **16** | LT01-1 | *S_2_* | *S_8_* | SC | 24.83222 | 116.63278 | Ningtian Town, Shanghang County, Longyan City, Fujian Province | SRR14761133 | Wang et al., 2022 ^1^ |
| **17** | LY04 | *S_2_* | *S_28_* | SC | 29.326366 | 121.65908 | Liyang Town, Ninghai County, Ningbo City, Zhejiang Province | SRR14761132 | Wang et al., 2022 ^1^ |
| **18** | LS | *S_8_* | *S_29_* | SI | # | # | Yongding County, Longyan City, Fujian Province | SRR14761135 | Wang et al., 2022 ^1^ |
| **19** | ZX11 | *S_2_* | *S_28_* | SC | 27.432423 | 120.46543 | Cangnan County, Wenzhou City, Zhejiang Province | SRR14761108 | Wang et al., 2022 ^1^ |
| **20** | CZ12 | *S_2_* | *S_32_* | SC | # | # | Suxian District, Chenzhou City, Hunan Province | SRR14761125 | Wang et al., 2022 ^1^ |
| **21** | SY02-1 | *S_2_* | *S_4_* | SC | 26.80961 | 119.72603 | Wanwu Township, Fu’an City, Fujian Province | SRR14761117 | Wang et al., 2022 ^1^ |
| **22** | MJS02 | *S_2_* | *S_28_* | SC | 28.766756 | 121.27971 | Meijian Mountain, Linhai City, Zhejiang Province | SRR14761131 | Wang et al., 2022 ^1^ |
| **23** | CH01-4 | *S_2_* | *S_32_* | SC | 26.45308 | 118.04528 | Gaosha Town, Sha County, Sanming City, Fujian Province | SRR14761136 | Wang et al., 2022 ^1^ |
| **24** | SF11 | *S_2_* | *S_28_* | SC | 27.319315 | 120.51564 | Cangnan County, Wenzhou City, Zhejiang Province | SRR14761121 | Wang et al., 2022 ^1^ |
| **25** | DR01-1 | *S_3_* | *S_8_* | SI | 24.76708 | 116.83967 | Hulei Town, Yongding County, Longyan City, Fujian Province | SRR14761107 | Wang et al., 2022 ^1^ |
| **26** | GT01 | *S_2_* | *S_32_* | SC | 26.02639 | 113.18708 | Gaoma Township, Zixing City, Hunan Province | SRR14761142 | Wang et al., 2022 ^1^ |
| **27** | JK09-1 | *S_29_* | *S_32_* | SI | 26.64144 | 118.00289 | Jukou Township, Yanping District, Nanping City, Fujian Province | SRR14761138 | Wang et al., 2022 ^1^ |
| **28** | BLS07-4 | *S_4_* | *S_30_* | SI | 26.03469 | 117.41853 | Gongchuan Town, Yong’an City, Sanming City, Fujian Province | SRR14761148 | Wang et al., 2022 ^1^ |
| **29** | TZ | *S_9_* | *S_23_* | SI | 24.75639 | 116.52472 | Hongshan Township, Yongding County, Longyan City, Fujian Province | SRR14761115 | Wang et al., 2022 ^1^ |
| **30** | YSY02 | *S_11_* | *S_17_* | SI | 24.7425 | 116.82111 | Hulei Town, Yongding County, Longyan City, Fujian Province | SRR14761110 | Wang et al., 2022 ^1^ |
| **31** | GH01-5 | *S_2_* | *S_32_* | SC | 27.6 | 118.73333 | Huaqiao Township, Songxi County, Nanping City, Fujian Province | SRR14761144 | Wang et al., 2022 ^1^ |
| **32** | BZ01-2 | *S_11_* | *S_23_* | SI | # | # | Shunchang County, Nanping City, Fujian Province | SRR14761147 | Wang et al., 2022 ^1^ |
| **33** | XHS | *S_2_* | *S_32_* | SC | 25.21536 | 117.11242 | Yanshi Town, Silla District, Longyan City, Fujian Province | SRR14761111 | Wang et al., 2022 ^1^ |
| **34** | NQ03-5 | *S_1_* | *S_2_* | SC | 26.19019 | 116.77139 | Longjin Town, Qingliu County, Sanming City, Fujian Province | SRR14761129 | Wang et al., 2022 ^1^ |
| **35** | ZL01-2 | *S_17_* | *S_22_* | SI | 24.77153 | 116.84806 | Hulei Town, Yongding County, Longyan City, Fujian Province | SRR14761109 | Wang et al., 2022 ^1^ |
| **36** | SL01-5 | *S_26_* | *S_31_* | SI | 24.81136 | 116.71378 | Hongshan Township, Yongding County, Longyan City, Fujian Province | SRR14761119 | Wang et al., 2022 ^1^ |
| **37** | RYS | *S_2_* | *S_5_* | SC | 26.34018 | 117.64986 | ChenDa Town, Meilie District, Sanming City, Fujian Province | SRR14761123 | Wang et al., 2022 ^1^ |
| **38** | WH | *S_2_* | *S_19_* | SC | 25.59122 | 116.75356 | Wenheng Town, Liancheng County, Longyan City, Fujian Province | SRR14761113 | Wang et al., 2022 ^1^ |
| **39** | PN03 | *S_4_* | *S_7_* | SI | # | # | Puning City, Jieyang City, Guangdong Province | SRR14761127 | Wang et al., 2022 ^1^ |
| **40** | PN01 | *S_7_* | *S_28_* | SI | # | # | Puning City, Jieyang City, Guangdong Province | SRR14761128 | Wang et al., 2022 ^1^ |
| **41** | FC14 | *S_14_* | *S_34_* | SI | 24.615 | 116.79472 | Xiayang Town, Yongding County, Longyan City, Fujian Province | SRR14761146 | Wang et al., 2022 ^1^ |
| **42** | FC-7 | *S_10_* | *S_14_* | SI | 24.615 | 116.79472 | Xiayang Town, Yongding County, Longyan City, Fujian Province | SRR14761145 | Wang et al., 2022 ^1^ |
| **43** | SY01-1 | *S_17_* | *S_26_* | SI | 26.80961 | 119.72603 | Wanwu Township, Fu’an City, Fujian Province | SRR14761118 | Wang et al., 2022 ^1^ |
| **44** | LS4 | *S_4_* | *S_11_* | SI | # | # | Yongding County, Longyan City, Fujian Province, Inotian Town | SRR14761134 | Wang et al., 2022 ^1^ |
| **45** | PN02 | *S_8_* | *S_19_* | SI | # | # | Puning City, Jieyang City, Guangdong Province | SRR14761126 | Wang et al., 2022 ^1^ |
| **46** | DB02 | *S_2_* | *S_29_* | SC | # | # | Dingnan County, Ganzhou City, Jiangxi Province | SRR14761114 | Wang et al., 2022 ^1^ |

**Supplementary Table 5. List of the significant integrated haplotype score measures (iHS) for the single-nucleotide polymorphism (SNP) markers and their closest genes**

| **CHR** | **Start** | **End** | **No. of SNP** | **Mean iHS value** | **Candidate Gene** |
| --- | --- | --- | --- | --- | --- |
| chr1 | 1040001 | 1070000 | 5 | 3.448 | *FhiS_2_*-SLF9, *FhiS_2_*-SLF8, *FhiS_2_*-SLF7, *FhiS_2_*-SLF6, *FhiS_2_*-RNase |
| chr1 | 1070001 | 1100000 | 6 | 3.347 | *FhiS_2_*-SLF5 |
| chr1 | 1100001 | 1130000 | 13 | 3.486 | *FhiS_2_*-SLF4 |
| chr1 | 1130001 | 1160000 | 50 | 3.567 | *FhiS_2_*-SLF3, Fh1g01700, *FhiS_2_*-SLF2 |
| chr1 | 1160001 | 1190000 | 1 | 4.002 | *FhiS_2_*-SLF1, Fh1g01730, Fh1g01740 |

**Supplementary Table 6. SC-related genomic region with mean XP-EHH value > 1 and associated genes**

| **CHR** | **Start** | **End** | **No. of SNP** | **Mean XP-EHH value** | **Candidate Gene** |
| --- | --- | --- | --- | --- | --- |
| chr1 | 1040001 | 1070000 | 54 | 1.093 | *FhiS_2_*-SLF9, *FhiS_2_*-SLF8, *FhiS_2_*-SLF7, *FhiS_2_*-SLF6, *FhiS_2_*-RNase |
| chr1 | 1070001 | 1100000 | 91 | 1.229 | *FhiS_2_*-SLF5 |
| chr1 | 1100001 | 1130000 | 77 | 1.15 | *FhiS_2_*-SLF4 |
| chr1 | 1130001 | 1160000 | 17 | 1.096 | *FhiS_2_*-SLF3, Fh1g01700, *FhiS_2_*-SLF2 |
| chr1 | 1160001 | 1190000 | 17 | 1.067 | *FhiS_2_*-SLF1, Fh1g01730, Fh1g01740 |
| chr1 | 1190001 | 1220000 | 14 | 1.063 | Fh1g01740, Fh1g01750, Fh1g01760, Fh1g01770 |
| chr1 | 1220001 | 1250000 | 0 | — | — |
| chr1 | 1250001 | 1280000 | 0 | — | — |
| chr1 | 1280001 | 1310000 | 5 | 1.038 | Fh1g01870, Fh1g01880, Fh1g01890, Fh1g01900, Fh1g01910, Fh1g01920 |
| chr5 | 15010001 | 15040000 | 8 | 1.053 | Fh5g17770, Fh5g17780 |
| chr5 | 33920001 | 33950000 | 9 | 1.033 | — |
| chr5 | 33950001 | 33980000 | 30 | 1.057 | Fh5g30040, Fh5g30050 |
| chr9 | 7730001 | 7760000 | 22 | 1.041 | Fh9g10380, Fh9g10390, Fh9g10400, Fh9g10410, Fh9g10420 |
| chr9 | 7840001 | 7870000 | 19 | 1.052 | Fh9g10480, Fh9g10490 |

**Supplementary Table 7. Mapping summary for WGBS reads**

| **Type** | **Leaf-Rep1** | **Leaf-Rep2** | **Anther-Rep1** | **Anther-Rep2** | **Style-Rep1** | **Style-Rep2** |
| --- | --- | --- | --- | --- | --- | --- |
| Total Reads | 75377325 | 77867232 | 75323767 | 75189797 | 56778096 | 74193037 |
| Aligned Reads | 40998829 | 43306387 | 36520373 | 37377966 | 30922241 | 38100049 |
| Unaligned Reads | 17471420 | 18044877 | 17794436 | 16704355 | 13849452 | 16896171 |
| Ambiguously Aligned Reads | 16907076 | 16515968 | 21008958 | 21107476 | 12006403 | 19196817 |
| No Genomic Sequence | 75 | 86 | 69 | 84 | 68 | 95 |
| Mapping rate | 76.82% | 76.83% | 76.38% | 77.78% | 75.61% | 77.23% |
| mean reads coverage | 24.74 | 26.17 | 22.03 | 22.55 | 18.67 | 22.98 |
| SRR id in NCBI | SRR27610094 | SRR27610095 | SRR27610090 | SRR27610091 | SRR27610092 | SRR27610093 |

**Supplementary Table 8. Summary information of DNA methylation in leaf, anther, and style tissues from DB02**

| **Type** | **Leaf-Rep1** | **Leaf-Rep2** | **Anther-Rep1** | **Anther-Rep2** | **Style-Rep1** | **Style-Rep2** |
| --- | --- | --- | --- | --- | --- | --- |
| total analysed C’s | 1532348478 | 1590190523 | 1407447973 | 1437001594 | 1093357907 | 1432399666 |
| methylated CpG | 145756162 | 137862236 | 153552858 | 157775026 | 88070771 | 144251518 |
| unmethylated CpG | 81406444 | 85186415 | 73327275 | 73124180 | 61514291 | 70783154 |
| methylated CHG | 73614041 | 74597572 | 69096353 | 71668356 | 41858578 | 72104649 |
| unmethylated CHG | 172019162 | 177112285 | 161219052 | 162706276 | 125311198 | 159396452 |
| methylated CHH | 100600665 | 101766155 | 62328120 | 65484502 | 44079986 | 70878399 |
| unmethylated CHH | 958952004 | 1013665860 | 887924315 | 906243254 | 732523083 | 914985494 |
| methylated CN | 48265 | 45897 | 42175 | 43721 | 26566 | 42210 |
| unmethylated CN | 445584 | 474198 | 433811 | 434567 | 409559 | 431480 |
| total methylated C’s | 320019133 | 314271860 | 285019506 | 294971605 | 174035901 | 287276776 |
| total C’s on genome | 55660049 | 55660049 | 55660049 | 55660049 | 55660049 | 55660049 |
| detected C’s | 1532842327 | 1590710618 | 1407923959 | 1437479882 | 1093794032 | 1432873356 |
| detected rate | 27.53936359 | 28.57903733 | 25.29505425 | 25.8260621 | 19.65133074 | 25.7433003 |
| **#methylation rates in different contexts against total methylated contexts** | | | | | | |
| mCpG | 0.641638008 | 0.618081461 | 0.676801692 | 0.683306923 | 0.588767152 | 0.670829112 |
| mCHG | 0.299690922 | 0.296363332 | 0.300007518 | 0.305785466 | 0.25039561 | 0.311465685 |
| mCHH | 0.094946356 | 0.091234745 | 0.065591118 | 0.067389762 | 0.056759995 | 0.07189471 |
| mCN | 0.097732303 | 0.088247339 | 0.088605547 | 0.091411451 | 0.060913729 | 0.089108911 |
| mC | 0.208842269 | 0.197631577 | 0.202508023 | 0.205268808 | 0.1591756 | 0.200556299 |
| **#methylation rates in different contexts against total mCs** | | | | | | |
| CpG | 0.455460774 | 0.438671907 | 0.538745085 | 0.534882081 | 0.506049444 | 0.502134283 |
| CHG | 0.230030124 | 0.237366374 | 0.242426752 | 0.242966966 | 0.240516915 | 0.250993658 |
| CHH | 0.314358282 | 0.323815677 | 0.218680191 | 0.222002731 | 0.253280994 | 0.246725127 |
| Mapping efficiency | 54.40% | 55.60% | 48.50% | 49.70% | 54.50% | 51.40% |

**Supplementary Table 9. Summary of small RNA sequencing data for the anther and style tissues from DB02**

| **Sample** | **Clean reads** | **Unique reads** | **Mapping rate (%)** | **SRA accession** |
| --- | --- | --- | --- | --- |
| DB02-Anther_rep1 | 22,887,429 | 5,405,812 | 62.94% | SRR18778190 |
| DB02-Anther_rep2 | 20,776,482 | 5,494,103 | 64.28% | SRR18778190 |
| DB02-Style_rep1 | 28,446,304 | 8,609,107 | 68.56% | SRR18778189 |
| DB02-Style_rep2 | 22,938,825 | 7,622,198 | 66.90% | SRR18778189 |

**Supplementary Table 10.** **List of siRNAs associated with flanking regions of *FhiS_2_-RNase* allele in anther and style tissues (RPM ≥ 2)**

| **Sequence of siRNAs** | **Strand** | **Length (nt)** | **Normalized expression (RPM, reads per million)** | | | |
| --- | --- | --- | --- | --- | --- | --- |
|  |  |  | **DB02-Anther-Rep1** | **DB02-Anther-Rep2** | **DB02-Style-Rep1** | **DB02-Style-Rep2** |
| ACAAGGCAGTCGCCTAAGGCCTCA | + | 24 | 10.41107433 | 8.564413282 | 35.80137152 | 18.85631722 |
| GAACAAGGCAGTCGCCTAAGGCCT | - | 24 | 12.4932892 | 12.51721941 | 32.09778136 | 27.34165997 |
| CGAACAAGGCAGTCGCCTAAGGCC | + | 24 | 5.899608787 | 9.882015325 | 28.1884362 | 18.62061325 |
| TTTAGGTATAGGCGAACAAGGCAG | - | 24 | 9.022931085 | 3.95280613 | 21.60427592 | 17.67779739 |
| AACAAGGCAGTCGCCTAAGGCCTC | - | 24 | 2.776286488 | 5.270408173 | 16.25464569 | 16.49927757 |
| AACAAGGCAGTCGCCTAAGGCCTC | + | 24 | 4.858501354 | 5.270408173 | 16.04889068 | 16.2635736 |
| TCATATTTTAAGAAGGCCTCATTT | - | 24 | 5.205537165 | 2.635204087 | 13.37407557 | 8.013934818 |
| GCGAACAAGGCAGTCGCCTAAGGC | - | 24 | 5.205537165 | 9.223214303 | 11.7280355 | 10.60667844 |
| GAACAAGGCAGTCGCCTAAGGCCT | + | 24 | / | 4.282206641 | 9.258975394 | 12.96371809 |
| CAAGGCAGTCGCCTAAGGCCTCA | + | 23 | 4.511465543 | 4.282206641 | 7.818690332 | 6.128303096 |
| CTCATATTTTAAGAAGGCCTCATT | - | 24 | 3.123322299 | 2.964604598 | 7.407180315 | 8.956750679 |
| TTAGGTATAGGCGAACAAGGCAGT | - | 24 | / | 2.635204087 | 6.995670297 | 6.835414992 |
| TTTTAAGAAGGCCTCATTTTTTGA | - | 24 | 2.429250677 | / | 6.995670297 | 5.185487235 |
| ACAAGGCAGTCGCCTAAGGCCTC | - | 23 | / | / | 6.58416028 | 6.835414992 |
| CGAACAAGGCAGTCGCCTAAGGCC | - | 24 | 2.429250677 | 5.270408173 | 6.378405271 | 4.714079305 |
| TAGGTATAGGCGAACAAGGCAGTC | - | 24 | / | / | 6.172650262 | 5.421191201 |
| CCTAAGGCCTCATATTTTAAGAAG | + | 24 | 2.776286488 | 4.611607152 | 6.172650262 | 11.07808637 |
| AGGTATAGGCGAACAAGGCAGTCG | + | 24 | / | / | 5.966895254 | 3.535559479 |
| TTAGGTATAGGCGAACAAGGCAGT | + | 24 | 3.47035811 | / | 5.761140245 | 5.421191201 |
| CAAGGCAGTCGCCTAAGGCCTCAT | + | 24 | 2.776286488 | 2.635204087 | 5.143875219 | 3.299855513 |
| ATTTTAAGAAGGCCTCATTTTTTG | + | 24 | 2.429250677 | / | 5.143875219 | 3.064151548 |
| CAAGGCAGTCGCCTAAGGCCT | + | 21 | / | / | 4.526610192 | 4.94978327 |
| CTTTAGGTATAGGCGAACAAGGCA | + | 24 | / | / | 3.497835149 | 2.121335687 |
| CATATTTTAAGAAGGCCTCATTTT | - | 24 | / | / | 3.086325131 | 2.828447583 |
| GGTATAGGCGAACAAGGCAGTCGC | - | 24 | 2.082214866 | / | 2.057550087 | 2.357039652 |
| CAAGGCAGTCGCCTAAGGCCTCAT | - | 24 | / | / | 2.057550087 | / |
| AACAAGGCAGTCGCCTAAGGCCT | + | 23 | / | / | 2.057550087 | / |
| AGGTATAGGCGAACAAGGCAGTCG | - | 24 | / | / | 2.057550087 | 2.357039652 |
| CTTTAGGTATAGGCGAACAAGGCA | - | 24 | / | / | / | 2.592743618 |
| CATAAGAAAATTTCGCCTAAGGCC | + | 24 | 8.328859463 | 7.246811238 | 22.63305096 | 19.56342912 |
| ATAAGAAAATTTCGCCTAAGGCCC | + | 24 | 6.593680408 | 9.223214303 | 22.42729595 | 12.72801412 |
| TAAGAAAATTTCGCCTAAGGCCCC | + | 24 | 2.776286488 | 7.246811238 | 16.66615571 | 11.78519826 |
| ATAAATAGAGGCCTCATAAGAAAA | + | 24 | 2.429250677 | 2.305803576 | 13.16832056 | 8.721046714 |
| AATAAATAGAGGCCTCATAAGAAA | + | 24 | / | 2.305803576 | 11.7280355 | 5.185487235 |
| AATTACAAAATAAATAGAGGCCTC | + | 24 | / | 3.623405619 | 10.08199543 | 6.835414992 |
| ATTACAAAATAAATAGAGGCCTCA | + | 24 | 2.082214866 | 2.305803576 | 8.847465376 | 7.306822923 |
| AAGAAAATTTCGCCTAAGGCCCCT | + | 24 | 3.47035811 | / | 7.818690332 | 7.071118957 |
| ACAAAATAAATAGAGGCCTCATAA | - | 24 | / | 2.635204087 | 7.612935324 | 2.121335687 |
| TACAAAATAAATAGAGGCCTCATA | - | 24 | / | 3.294005108 | 6.58416028 | 4.47837534 |
| CCCTTAGACTCTAAAGCCGGCCCT | - | 24 | / | / | 6.58416028 | 4.242671374 |
| AAATAAATAGAGGCCTCATAAGAA | + | 24 | / | / | 6.378405271 | 6.128303096 |
| AAATAGAGGCCTCATAAGAAAATT | + | 24 | / | / | 5.143875219 | 2.592743618 |
| AGAGGCCTCATAAGAAAATTTCGC | + | 24 | / | / | 5.143875219 | 2.357039652 |
| AATAGAGGCCTCATAAGAAAATTT | + | 24 | / | / | 4.93812021 | 3.064151548 |
| AGAAAATTTCGCCTAAGGCCCCTT | + | 24 | / | / | 4.93812021 | 8.249638784 |
| AAAATAAATAGAGGCCTCATAAGA | + | 24 | / | / | 4.732365201 | / |
| AAATTACAAAATAAATAGAGGCCT | + | 24 | 2.776286488 | 2.635204087 | 4.320855184 | 4.242671374 |
| GAAATTACAAAATAAATAGAGGCC | + | 24 | / | / | 4.320855184 | 4.242671374 |
| TAGAGGCCTCATAAGAAAATTTC | - | 23 | / | / | 3.909345166 | 3.064151548 |
| TAGAGGCCTCATAAGAAAATTTCG | + | 24 | / | 2.305803576 | 3.497835149 | 2.357039652 |
| TAAATAGAGGCCTCATAAGAAAAT | + | 24 | / | / | 3.497835149 | 2.121335687 |
| GAAAATTTCGCCTAAGGCCCCTTA | - | 24 | / | / | 3.29208014 | / |
| AGGCCCCTTAGACTCTAAAGCCGG | - | 24 | / | 2.305803576 | 2.469060105 | 2.357039652 |
| CAAAATAAATAGAGGCCTCATAAG | + | 24 | / | / | 2.057550087 | 2.828447583 |
| ATAAATAGAGGCCTCATAAGAAAA | - | 24 | / | / | 2.057550087 | / |
| TTACAAAATAAATAGAGGCCTCAT | - | 24 | / | / | 2.057550087 | / |
| AAATTTCGCCTAAGGCCCCTTAGA | + | 24 | / | / | / | 2.357039652 |
| CCTCATAAGAAAATTTCGCCTAAG | + | 24 | / | / | / | 2.121335687 |

**Supplementary Table 11. Summary statistics for the assembly of 3 genome sequences**

| **Sequencing** |  | **DB02gv1 contig** | **PN02gv1 contig** | **AZMgv1 contig** |
| --- | --- | --- | --- | --- |
|  | Platform | PacBio Sequel Ⅱ | PacBio Sequel Ⅱ | PacBio Sequel Ⅱ |
|  | Clean data (Gb) | 200.16 | 191.98 | 129.79 |
|  | Library size | 30 kb | 30 kb | 30 kb |
| **Genome assembly** | **Assembly** | **Primary assembly** | | |
|  | Assembly length (bp) | 663,941,631 | 615,032,083 | 839,375,747 |
|  | Number of sequences | 2,083 | 3,171 | 6,687 |
|  | Average length (bp) | 318,754 | 193,955 | 125,523 |
|  | Maximum length (bp) | 9,103,030 | 10,458,091 | 36,006,770 |
|  | Minimum length (bp) | 1,060 | 7,370 | 2,049 |
|  | N50 length (bp) | 691,012 | 448,058 | 8,373,697 |
|  | GC content (%) | 36.0% | 34.8% | 35.9% |
| **BUSCO** | **Types** | **Percentage (%)** | | |
|  | Complete BUSCOs (C) | 98.9 | 98.9 | 98.9 |
|  | Complete Single-Copy BUSCOs (S) | 7.9 | 18.0 | 4.4 |
|  | Complete and duplicated BUSCOs (D) | 91.0 | 80.9 | 94.5 |
|  | Fragmented BUSCOs (F) | 0.4 | 0.4 | 0.3 |
|  | Missing BUSCOs (M) | 0.7 | 0.7 | 0.8 |
| **Assembly accession** |  | CNA0069197 | CNA0069198 | CNA0069199 |

**Supplementary Table 12. Summary of transposable elements in *S*-locus of *F. hindsii***

|  | **Fhi-*S_2_*-locus** | | | **Fhi-*S_8_*-locus** | | | **Fhi-*S_19_*-locus** | | | **Fhi-*S_29_*-locus** | | |
| --- | --- | --- | --- | --- | --- | --- | --- | --- | --- | --- | --- | --- |
|  | **number of elements** | **length occupied** | **percentage of sequence** | **number of elements** | **length occupied** | **percentage of sequence** | **number of elements** | **length occupied** | **percentage of sequence** | **number of elements** | **length occupied** | **percentage of sequence** |
| **Retroelements** | 34 | 112509 bp | 39.23% | 56 | 142408 bp | 37.67% | 67 | 147849 bp | 40.02% | 65 | 124305 bp | 39.52% |
| SINEs: | 0 | 0 bp | 0.00% | 0 | 0 bp | 0.00% | 0 | 0 bp | 0.00% | 0 | 0 bp | 0.00% |
| Penelope | 0 | 0 bp | 0.00% | 0 | 0 bp | 0.00% | 0 | 0 bp | 0.00% | 0 | 0 bp | 0.00% |
| LINEs: | 2 | 14322 bp | 4.99% | 2 | 9249 bp | 2.45% | 2 | 10530 bp | 2.85% | 2 | 12470 bp | 3.96% |
| CRE/SLACS | 0 | 0 bp | 0.00% | 0 | 0 bp | 0.00% | 0 | 0 bp | 0.00% | 0 | 0 bp | 0.00% |
| L2/CR1/Rex | 0 | 0 bp | 0.00% | 0 | 0 bp | 0.00% | 0 | 0 bp | 0.00% | 0 | 0 bp | 0.00% |
| R1/LOA/Jockey | 0 | 0 bp | 0.00% | 0 | 0 bp | 0.00% | 0 | 0 bp | 0.00% | 0 | 0 bp | 0.00% |
| R2/R4/NeSL | 0 | 0 bp | 0.00% | 0 | 0 bp | 0.00% | 0 | 0 bp | 0.00% | 0 | 0 bp | 0.00% |
| RTE/Bov-B | 0 | 0 bp | 0.00% | 0 | 0 bp | 0.00% | 0 | 0 bp | 0.00% | 0 | 0 bp | 0.00% |
| L1/CIN4 | 2 | 14322 bp | 4.99% | 2 | 9249 bp | 2.45% | 2 | 10530 bp | 2.85% | 2 | 12470 bp | 3.96% |
| LTR elements: | 32 | 98187 bp | 34.24% | 54 | 133159 bp | 35.22% | 65 | 137319 bp | 37.17% | 63 | 111835 bp | 35.55% |
| BEL/Pao | 0 | 0 bp | 0.00% | 0 | 0 bp | 0.00% | 0 | 0 bp | 0.00% | 0 | 0 bp | 0.00% |
| Ty1/Copia | 18 | 61275 bp | 21.36% | 24 | 72054 bp | 19.06% | 34 | 81750 bp | 22.13% | 31 | 61316 | 19.49% |
| Gypsy/DIRS1 | 11 | 25633 bp | 8.94% | 25 | 47614 bp | 12.59% | 25 | 37905 bp | 10.26% | 28 | 38833 bp | 12.35% |
| Retroviral | 2 | 10849 bp | 3.78% | 3 | 12897 bp | 3.41% | 6 | 17664 bp | 4.78% | 4 | 11686 bp | 3.72% |
| **DNA transposons** | 29 | 31181 bp | 10.87% | 44 | 45581 bp | 12.06% | 36 | 42751 bp | 11.57% | 27 | 35441 bp | 11.27% |
| hobo-Activator | 7 | 7042 bp | 2.46% | 6 | 3839 bp | 1.02% | 7 | 5468 bp | 1.48% | 5 | 5427 bp | 1.73% |
| Tc1-IS630-Pogo | 0 | 0 bp | 0.00% | 0 | 0 bp | 0.00% | 0 | 0 bp | 0.00% | 0 | 0 bp | 0.00% |
| En-Spm | 0 | 0 bp | 0.00% | 0 | 0 bp | 0.00% | 0 | 0 bp | 0.00% | 0 | 0 bp | 0.00% |
| MuDR-IS905 | 0 | 0 bp | 0.00% | 0 | 0 bp | 0.00% | 0 | 0 bp | 0.00% | 0 | 0 bp | 0.00% |
| PiggyBac | 0 | 0 bp | 0.00% | 0 | 0 bp | 0.00% | 0 | 0 bp | 0.00% | 0 | 0 bp | 0.00% |
| Tourist/Harbinger | 0 | 0 bp | 0.00% | 0 | 0 bp | 0.00% | 1 | 26 bp | 0.01% | 0 | 0 bp | 0.00% |
| **Unclassified:** | 100 | 106486 bp | 37.13% | 148 | 137349 bp | 36.33% | 135 | 110871 bp | 30.01% | 119 | 108362 bp | 34.45% |
| **Total interspersed repeats:** | | 250176 bp | 87.23% |  | 325338 bp | 86.06% |  | 301471 bp | 81.61% |  | 268108 bp | 85.24% |
| **Simple repeats:** | 36 | 1931 bp | 0.67% | 54 | 2571 bp | 0.68% | 58 | 2834 bp | 0.77% | 35 | 1933 bp | 0.61% |
| **Low complexity:** | 9 | 486 bp | 0.17% | 9 | 478 bp | 0.13% | 10 | 755 bp | 0.20% | 9 | 419 bp | 0.13% |
| **Total length:** | **286802 bp** | | | **378055 bp** | | | **369415 bp** | | | **314547 bp** | | |

**Supplementary Table 13. Isolation and identification of *S*-locus *F-box* alleles in citrus**

| **No.** | **Gene** | **Organism** | **Full length (bp)** | **Amino acid (aa)** | **Molecular mass (kDa)** | **Isoelectric point** | **Source** | **Accession number** |
| --- | --- | --- | --- | --- | --- | --- | --- | --- |
| 1 | *FhiS_2_-SLF1* | *Fortunella hindsii* | 1101 | 366 | 42.97 | 5.01 | In this study | ON529712 |
| 2 | *FhiS_2_-SLF2* | *Fortunella hindsii* | 1116 | 371 | 43.39 | 4.73 | In this study | ON529713 |
| 3 | *FhiS_2_-SLF3* | *Fortunella hindsii* | 1101 | 366 | 43.15 | 4.93 | In this study | ON529714 |
| 4 | *FhiS_2_-SLF4* | *Fortunella hindsii* | 1104 | 367 | 43.51 | 6.21 | In this study | ON529715 |
| 5 | *FhiS_2_-SLF5* | *Fortunella hindsii* | 1125 | 374 | 43.47 | 5.46 | In this study | ON529716 |
| 6 | *FhiS_2_-SLF6* | *Fortunella hindsii* | 1143 | 380 | 44.32 | 5.62 | In this study | ON529717 |
| 7 | *FhiS_2_-SLF7* | *Fortunella hindsii* | 1131 | 376 | 43.64 | 5.09 | In this study | ON529718 |
| 8 | *FhiS_2_-SLF8* | *Fortunella hindsii* | 1143 | 380 | 44.44 | 5.83 | In this study | ON529719 |
| 9 | *FhiS_2_-SLF9* | *Fortunella hindsii* | 1140 | 379 | 44.01 | 5.37 | In this study | ON529720 |
| 10 | *FhiS_2_-SLF10* | *Fortunella hindsii* | 1146 | 381 | 44.86 | 5.08 | In this study | ON529721 |
| 11 | *FhiS_2_-SLF11* | *Fortunella hindsii* | 1176 | 391 | 45.52 | 5.06 | In this study | ON529722 |
| 12 | *FhiS_2_-SLF12* | *Fortunella hindsii* | 1122 | 373 | 43.04 | 5.45 | In this study | ON529723 |
| 13 | *FhiS_2_-SLF13* | *Fortunella hindsii* | 1077 | 358 | 41.75 | 5.28 | In this study | ON529724 |
| 14 | *FhiS_2_-SLF14* | *Fortunella hindsii* | 1113 | 370 | 42.73 | 5.05 | In this study | ON529725 |
| 15 | *FhiS_8_-SLF1* | *Fortunella hindsii* | 1101 | 366 | 43.02 | 5.09 | In this study | ON529726 |
| 16 | *FhiS_8_-SLF2* | *Fortunella hindsii* | 1125 | 374 | 43.62 | 5.1 | In this study | ON529727 |
| 17 | *FhiS_8_-SLF3* | *Fortunella hindsii* | 1122 | 373 | 44.21 | 4.91 | In this study | ON529728 |
| 18 | *FhiS_8_-SLF4* | *Fortunella hindsii* | 1116 | 371 | 43.6 | 5.37 | In this study | ON529729 |
| 19 | *FhiS_8_-SLF5* | *Fortunella hindsii* | 1125 | 374 | 43.65 | 5.03 | In this study | ON529730 |
| 20 | *FhiS_8_-SLF6* | *Fortunella hindsii* | 1140 | 379 | 44.19 | 5.8 | In this study | ON529731 |
| 21 | *FhiS_8_-SLF7* | *Fortunella hindsii* | 1143 | 380 | 44.5 | 5.65 | In this study | ON529732 |
| 22 | *FhiS_8_-SLF8* | *Fortunella hindsii* | 1113 | 370 | 43.28 | 5.65 | In this study | ON529733 |
| 23 | *FhiS_8_-SLF9a* | *Fortunella hindsii* | 1140 | 379 | 44 | 5.45 | In this study | ON529734 |
| 24 | *FhiS_8_-SLF9b* | *Fortunella hindsii* | 1146 | 381 | 44.43 | 5.81 | In this study | ON529735 |
| 25 | *FhiS_8_-SLF10* | *Fortunella hindsii* | 1146 | 381 | 44.82 | 5.03 | In this study | ON529736 |
| 26 | *FhiS_8_-SLF11* | *Fortunella hindsii* | 1170 | 389 | 45.27 | 5.06 | In this study | ON529749 |
| 27 | *FhiS_8_-SLF12* | *Fortunella hindsii* | 1116 | 371 | 42.83 | 5.38 | In this study | ON529737 |
| 28 | *FhiS_8_-SLF13a* | *Fortunella hindsii* | 1077 | 358 | 41.79 | 5.7 | In this study | ON529751 |
| 29 | *FhiS_8_-SLF13b* | *Fortunella hindsii* | 1113 | 370 | 42.63 | 4.94 | In this study | ON529738 |
| 30 | *FhiS_19_-SLF1* | *Fortunella hindsii* | 1104 | 367 | 43.06 | 4.96 | In this study | ON529739 |
| 31 | *FhiS_19_-SLF2* | *Fortunella hindsii* | 1125 | 374 | 43.74 | 4.78 | In this study | ON529740 |
| 32 | *FhiS_19_-SLF3* | *Fortunella hindsii* | 1122 | 373 | 43.91 | 4.79 | In this study | ON529741 |
| 33 | *FhiS_19_-SLF4* | *Fortunella hindsii* | 1113 | 370 | 43.98 | 6.08 | In this study | ON529742 |
| 34 | *FhiS_19_-SLF5* | *Fortunella hindsii* | 1125 | 374 | 43.62 | 4.89 | In this study | ON529743 |
| 35 | *FhiS_19_-SLF6* | *Fortunella hindsii* | 1140 | 379 | 44.12 | 5.55 | In this study | ON529744 |
| 36 | *FhiS_19_-SLF7* | *Fortunella hindsii* | 1134 | 377 | 43.66 | 5.09 | In this study | ON529745 |
| 37 | *FhiS_19_-SLF8* | *Fortunella hindsii* | 1137 | 378 | 44.18 | 5.54 | In this study | ON529746 |
| 38 | *FhiS_19_-SLF9* | *Fortunella hindsii* | 1146 | 381 | 44.63 | 5.34 | In this study | ON529747 |
| 39 | *FhiS_19_-SLF10* | *Fortunella hindsii* | 1146 | 381 | 44.81 | 5.03 | In this study | ON529748 |
| 40 | *FhiS_19_-SLF11* | *Fortunella hindsii* | 1170 | 389 | 45.27 | 5.06 | In this study | ON529749 |
| 41 | *FhiS_19_-SLF12* | *Fortunella hindsii* | 1116 | 371 | 42.8 | 5.38 | In this study | ON529750 |
| 42 | *FhiS_19_-SLF13* | *Fortunella hindsii* | 1077 | 358 | 41.79 | 5.7 | In this study | ON529751 |
| 43 | *FhiS_19_-SLF14* | *Fortunella hindsii* | 1113 | 370 | 42.7 | 5.11 | In this study | ON529752 |
| 44 | *FhiS_29_-SLF1* | *Fortunella hindsii* | 1101 | 366 | 42.89 | 4.97 | In this study | ON529753 |
| 45 | *FhiS_29_-SLF2* | *Fortunella hindsii* | 1125 | 374 | 43.65 | 4.62 | In this study | ON529754 |
| 46 | *FhiS_29_-SLF3* | *Fortunella hindsii* | 1122 | 373 | 44.17 | 5.06 | In this study | ON529755 |
| 47 | *FhiS_29_-SLF4* | *Fortunella hindsii* | 1116 | 371 | 44.12 | 5.64 | In this study | ON529756 |
| 48 | *FhiS_29_-SLF5* | *Fortunella hindsii* | 1122 | 373 | 43.33 | 5.24 | In this study | ON529757 |
| 49 | *FhiS_29_-SLF6* | *Fortunella hindsii* | 1140 | 379 | 44.34 | 6.02 | In this study | ON529758 |
| 50 | *FhiS_29_-SLF7* | *Fortunella hindsii* | 1131 | 376 | 43.93 | 5.02 | In this study | ON529759 |
| 51 | *FhiS_29_-SLF8* | *Fortunella hindsii* | 1125 | 374 | 43.8 | 5.68 | In this study | ON529760 |
| 52 | *FhiS_29_-SLF10* | *Fortunella hindsii* | 1146 | 381 | 44.95 | 5.14 | In this study | ON529761 |
| 53 | *FhiS_29_-SLF11* | *Fortunella hindsii* | 1176 | 391 | 45.5 | 5.12 | In this study | ON529762 |
| 54 | *FhiS_29_-SLF12* | *Fortunella hindsii* | 1122 | 373 | 43.01 | 5.38 | In this study | ON529763 |
| 55 | *FhiS_29_-SLF13* | *Fortunella hindsii* | 1077 | 358 | 41.68 | 5.63 | In this study | ON529764 |
| 56 | *FhiS_29_-SLF14* | *Fortunella hindsii* | 1113 | 370 | 42.66 | 5.11 | In this study | ON529765 |
| 57 | *CgrS_1_-SLF1* | *Citrus maxima* | 1101 | 366 | 43.23 | 4.96 | In this study | ON529766 |
| 58 | *CgrS_1_-SLF2* | *Citrus maxima* | 1098 | 365 | 42.41 | 4.94 | In this study | ON529767 |
| 59 | *CgrS_1_-SLF3* | *Citrus maxima* | 1122 | 373 | 44.1 | 4.85 | In this study | ON529768 |
| 60 | *CgrS_1_-SLF4* | *Citrus maxima* | 1116 | 371 | 43.75 | 5.68 | In this study | ON529769 |
| 61 | *CgrS_1_-SLF5* | *Citrus maxima* | 1131 | 376 | 43.74 | 4.83 | In this study | ON529770 |
| 62 | *CgrS_1_-SLF6* | *Citrus maxima* | 1119 | 372 | 43.2 | 5.42 | In this study | ON529771 |
| 63 | *CgrS_1_-SLF7* | *Citrus maxima* | 1137 | 378 | 44.06 | 5.25 | In this study | ON529772 |
| 64 | *CgrS_1_-SLF8* | *Citrus maxima* | 1140 | 379 | 44.12 | 5.68 | In this study | ON529773 |
| 65 | *CgrS_1_-SLF9* | *Citrus maxima* | 1140 | 379 | 43.97 | 5.7 | In this study | ON529774 |
| 66 | *CgrS_1_-SLF10* | *Citrus maxima* | 1146 | 381 | 44.95 | 5.08 | In this study | ON529775 |
| 67 | *CgrS_1_-SLF11* | *Citrus maxima* | 1176 | 391 | 45.55 | 5.06 | In this study | ON529776 |
| 68 | *CgrS_1_-SLF12* | *Citrus maxima* | 1122 | 373 | 43.07 | 5.38 | In this study | ON529777 |
| 69 | *CgrS_1_-SLF13* | *Citrus maxima* | 1077 | 358 | 41.6 | 5.36 | In this study | ON529778 |
| 70 | *CgrS_2_-SLF1* | *Citrus maxima* | 1101 | 366 | 43.09 | 5.15 | In this study | ON529779 |
| 71 | *CgrS_2_-SLF2* | *Citrus maxima* | 1116 | 371 | 43.45 | 4.71 | In this study | ON529780 |
| 72 | *CgrS_2_-SLF3* | *Citrus maxima* | 1122 | 373 | 43.88 | 4.93 | In this study | ON529781 |
| 73 | *CgrS_2_-SLF4* | *Citrus maxima* | 1104 | 367 | 43.63 | 6.56 | In this study | ON529782 |
| 74 | *CgrS_2_-SLF5* | *Citrus maxima* | 1125 | 374 | 43.39 | 5.14 | In this study | ON529783 |
| 75 | *CgrS_2_-SLF6* | *Citrus maxima* | 1134 | 377 | 43.71 | 5.91 | In this study | ON529784 |
| 76 | *CgrS_2_-SLF7* | *Citrus maxima* | 1131 | 376 | 43.84 | 4.79 | In this study | ON529785 |
| 77 | *CgrS_2_-SLF8* | *Citrus maxima* | 1146 | 381 | 44.31 | 5.4 | In this study | ON529786 |
| 78 | *CgrS_2_-SLF9* | *Citrus maxima* | 1140 | 379 | 43.8 | 6.35 | In this study | ON529787 |
| 79 | *CgrS_2_-SLF10* | *Citrus maxima* | 1146 | 381 | 44.92 | 5.03 | In this study | ON529788 |
| 80 | *CgrS_2_-SLF11* | *Citrus maxima* | 1176 | 391 | 45.55 | 5.06 | In this study | ON529789 |
| 81 | *CgrS_2_-SLF12* | *Citrus maxima* | 1122 | 373 | 43.09 | 5.38 | In this study | ON529790 |
| 82 | *CgrS_2_-SLF13* | *Citrus maxima* | 1077 | 358 | 41.65 | 5.36 | In this study | ON529791 |

**Supplementary Table 14. Detailed information relating to whole-sequence alignments of *S*-locus**

| **No.** | **Accession name** | **Catalog** | **Scientific name** | **Genome** | ***S-RNase* Position** | **Source** | **Genome accession** |
| --- | --- | --- | --- | --- | --- | --- | --- |
| **1** | Cgr-*S_1_*-locus | Pummelo | *Citrus maxima* | Gygv1 contig | tig00000920:336034-336821 | Hu et al., 2021 ^4^ | CNP0001706 |
| **2** | Cgr-*S_2_*-locus | Pummelo | *Citrus maxima* | Gygv1 contig | tig00000926:418899-419689 | Hu et al., 2021 ^4^ | CNP0001706 |
| **3** | Cau-*S_8_*-locus | Australian lime | *Citrus australasica* | Citrus australasica v1.0 contig | contig66:200851-201677 | Liu et al., 2022 ^5^ | http://citrus.hzau.edu.cn/ |
| **4** | Cle-*S_11_*-locus | Clementine | *Citrus clementina* | Citrus clementina v1.0 scaffold | scaffold_7:1103445-1104241 | Wu et al., 2014 ^6^ | PRJNA223006 |
| **5** | Fhi-*S_2_*-locus | Hongkong kumquat | *Fortunella hindsii* | Fortunella hindsii v1.0 contig | tig00040105_arrow_pilon:5863264-5864089 | Zhu et al., 2019 ^7^ | PRJNA487160 |
| **6** | Cho-*S_17_*-locus | Honghe papeda | *Citrus hongheensis* | Citrus hongheensis v1.0 contig | contig70:303066-303842 | Liu et al., 2022 ^5^ | http://citrus.hzau.edu.cn/ |
| **7** | Cho-*S_19_*-locus | Honghe papeda | *Citrus hongheensis* | Citrus hongheensis v1.0 contig | contig630:111757-112538 | Liu et al., 2022 ^5^ | http://citrus.hzau.edu.cn/ |
| **8** | Abu-*S_2_*-locus | Atalantia | *Atalantia buxifolia* | Atalantia buxfoliata v1.0 scaffold | scaffold25399:150188-151000 | Wang et al., 2017 ^8^ | PRJNA327148 |
| **9** | Abu-*S_12_*-locus | Atalantia | *Atalantia buxifolia* | Atalantia buxfoliata v1.0 scaffold | scaffold20559:1882591-1883419 | Wang et al., 2017 ^8^ | PRJNA327148 |
| **10** | Abu-*S_22_*-locus | Atalantia | *Atalantia buxifolia* | Atalantia buxfoliata v2.0 chrmosome | chr1:436023-436937 | Liu et al., 2022 ^5^ | http://citrus.hzau.edu.cn/ |
| **11** | Cgr-*S_6_*-locus | Pummelo | *Citrus maxima* | Citrus grandis (L.) Osbeck.cv.’Wanbaiyou’ v1.0 chromsome | chr1:1175531-1176277 | Wang et al., 2017 ^8^ | PRJNA318855 |
| **13** | Cre-*S_10_*-locus | Mandarin | *Citrus reticulata* | Citrus reticulata v1.0 scaffold | scaffold85925_cov91:2003156-2003983 | Wang et al., 2018 ^9^ | PRJNA388397 |
| **14** | Cre-*S_m_*-locus | Mandarin | *Citrus reticulata* | Citrus reticulata v1.0 scaffold | scaffold85973_cov87:70583-71361 | Wang et al., 2018 ^9^ | PRJNA388397 |
| **15** | Cre-*S_m_*-locus | Mandarin | *Citrus reticulata* | Citrus reticulata v2.0 chromosome | chr1:28868401-28869183 | Liu et al., 2022 ^5^ | http://citrus.hzau.edu.cn/ |
| **17** | Cma-*S_2_*-locus | Wild mandarin | *Citrus mangshanensis* | Citrus mangshanensis v1.0 contig | contig285:7938886-7939676 | Liu et al., 2022 ^5^ | http://citrus.hzau.edu.cn/ |
| **18** | Cma-*S_23_*-locus | Wild mandarin | *Citrus mangshanensis* | Citrus mangshanensis v1.0 contig | contig135:131165-131945 | Liu et al., 2022 ^5^ | http://citrus.hzau.edu.cn/ |
| **19** | Cre-*S_2_*-locus | Cultivated mandarin | *Citrus reticulata* | Citrus reticulata Blanco cv. Ponkan v1.0 chromosome | chr1:35812311-35813101 | Liu et al., 2022 ^5^ | http://citrus.hzau.edu.cn/ |
| **20** | Cme-*S_14_*-locus | Citron | *Citrus medica* | Citrus medica v1.0 scaffold | scaffold_12:46448-47971 | Wang et al., 2017 ^8^ | PRJNA320023 |
| **21** | Cme-*S_30_*-locus | Citron | *Citrus medica* | Citrus medica v1.0 scaffold | scaffold_1716:7488-8292 | Wang et al., 2017 ^8^ | PRJNA320023 |
| **22** | Cme-*S_8_*-locus | Citron | *Citrus medica* | Citrus medica v2.0 contig | contig282:220269-221095 | Liu et al., 2022 ^5^ | http://citrus.hzau.edu.cn/ |
| **23** | Csi-*S_m_*-locus | Sweet orange | *Citrus sinensis* | Citrus sinensis v1.0 chromosome | chr1:27642952-27643734 | Xu et al., 2013 ^10^ | PRJNA86123 |
| **24** | Csi-*S_m_*-locus | Sweet orange | *Citrus sinensis* | Citrus sinensis v2.0 chromosome | chr1:28306923-28307705 | Wang et al., 2021 ^11^ | PRJNA347609 |
| **25** | Csi-*S_m_*-locus | Sweet orange | *Citrus sinensis* | Citrus sinensis v3.0 chromosome | chr1:29336694-29337476 | Wang et al., 2021 ^11^ | PRJNA347609 |
| **26** | Cgr-*S_18_*-locus | Pummelo | *Citrus maxima* | Citrus grandis (L.) Osbeck.cv.’Majiayou’ v1.0 contig | utg161:355879-356680 | Lu et al., 2022 ^12^ | PRJNA318855 |
| **27** | Cgr-*S_22_*-locus | Pummelo | *Citrus maxima* | Citrus grandis (L.) Osbeck.cv.’Majiayou’ v1.0 contig | utg1001:75925-76710 | Lu et al., 2022 ^12^ | PRJNA318855 |
| **28** | Cic-*S_13_*-locus | Ichang papeda | *Citrus ichangensis* | Citrus ichangensis v1.0 scaffold | scaffold_539:140624-141656 | Wang et al., 2017 ^8^ | PRJNA321657 |
| **29** | Cic-*S_31_*-locus | Ichang papeda | *Citrus ichangensis* | Citrus ichangensis v1.0 scaffold | scaffold_523:41129-41925 | Wang et al., 2017 ^8^ | PRJNA321657 |
| **30** | Cic-*S_14_*-locus | Ichang papeda | *Citrus ichangensis* | Citrus ichangensis v2.0 contig | contig157:262216-263739 | Liu et al., 2022 ^5^ | http://citrus.hzau.edu.cn/ |
| **31** | Cic-*S_26_*-locus | Ichang papeda | *Citrus ichangensis* | Citrus ichangensis v2.0 contig | contig334:235945-236743 | Liu et al., 2022 ^5^ | http://citrus.hzau.edu.cn/ |
| **32** | Cgr-*S_3_*-locus | Pummelo | *Citrus maxima* | Citrus grandis (L.) Osbeck.cv.’Zipiyou’ v1.0 chromosome | chr1:1028364-1029155 | Liu et al., 2022 ^5^ | http://citrus.hzau.edu.cn/ |
| **33** | Cgr-*S_5_*-locus | Pummelo | *Citrus maxima* | Citrus grandis (L.) Osbeck.cv.’Zipiyou’ v1.0 chromosome | contig89:165736-166769 | Liu et al., 2022 ^5^ | http://citrus.hzau.edu.cn/ |
| **34** | Ptr-*S_2_*-locus | Hardy orange | *Poncirus trifoliata* | Poncirus trifoliata v1.0 chromosome | chr7:20469272-20470060 | Huang et al., 2021 ^2^ | PRJNA554539 |
| **35** | Ptr-*S_31_*-locus | Hardy orange | *Poncirus trifoliata* | Poncirus trifoliata v1.0 chromosome | chr7:20174759-20175555 | Huang et al., 2021 ^2^ | PRJNA554539 |
| **36** | Cun-*S_m_*-locus | Satsuma | *Citrus unshiu* | Satsuma assembly unshiu v1.0 scaffold | scaffold00356:86101-86883 | Tokurou Shimizu et al., 2017 ^13^ | PRJDB5882 |
| **37** | Cun-*S_9_*-locus | Satsuma | *Citrus unshiu* | Satsuma assembly unshiu v1.0 scaffold | scaffold00169:436590-437366 | Tokurou Shimizu et al., 2017 ^13^ | PRJDB5882 |
| **38** | Csi-*S_m_*-locus | Sweet orange | *Citrus sinensis* | Citrus sinensis genome v1.1 (JGI) | scaffold07828:1662-2443 | Wu et al., 2014 ^6^ | PRJNA225968 |
| **39** | Csi-*S_7_*-locus | Sweet orange | *Citrus sinensis* | Citrus sinensis genome v1.1 (JGI) | scaffold11212:1912-2153 | Wu et al., 2014 ^6^ | PRJNA225968 |
| **40** | Ptr-*S_10_*-locus | Hardy orange | *Poncirus trifoliata* | P. trifoliata (v1.0) | P_trifoliata_00771:45845-46673 | Kawahara Y et al., 2020 ^14^ | https://mikan.dna.affrc.go.jp |
| **41** | Ptr-*S_30_*-locus | Hardy orange | *Poncirus trifoliata* | P. trifoliata (v1.0) | P_trifoliata_00865:22229-23033 | Kawahara Y et al., 2020 ^14^ | https://mikan.dna.affrc.go.jp |
| **42** | Ptr-*S_31_*-locus | Hardy orange | *Poncirus trifoliata* | P. trifoliata (v1.0) | P_trifoliata_00672:42673-43469 | Kawahara Y et al., 2020 ^14^ | https://mikan.dna.affrc.go.jp |
| **43** | Cgr-*S_17_*-locus | Pummelo | *Citrus maxima* | Citrus grandis (L.) Osbeck.cv. ‘Huajuhong’ v1.0 chromosome | chr1:586695-587471 | Zheng et al., 2023 ^15^ | PRJNA911419 |
| **44** | Fhi-*S_2_*-locus | Hongkong kumquat | *Fortunella hindsii* | DB02gv1 contig | tig00002062_pilon:1480026-1480851 | In this study | CNP0003278 |
| **45** | Fhi-*S_29_*-locus | Hongkong kumquat | *Fortunella hindsii* | DB02gv1 contig | tig00001167_pilon:222144-222918 | In this study | CNP0003278 |
| **46** | Fhi-*S_8_*-locus | Hongkong kumquat | *Fortunella hindsii* | PN02gv1 contig | tig00003332_pilon:390974-391801 | In this study | CNP0003278 |
| **47** | Fhi-*S_19_*-locus | Hongkong kumquat | *Fortunella hindsii* | PN02gv1 contig | tig00001555_pilon:278065-278846 | In this study | CNP0003278 |
| **48** | Cau*-S_8_*-locus | Australian lime | *Citrus australasica* | AZMgv1 contig | tig00000102:1193949-1194775 | In this study | CNP0003278 |
| **49** | Cau*-S_31_*-locus | Australian lime | *Citrus australasica* | AZMgv1 contig | tig00007128:1030109-1030905 | In this study | CNP0003278 |

The 200-bp sequences of two non-recombinant markers (K961397 and K1247997) were used as the boundaries of the *S*-locus.

>K961397-Seq200

TGCTATGTGTATATCAGAATGAACAGGGATCATAATTCATCAAATCCATGGACCACTTTCACTGTTTTCACGCGAAGCTGCAATATCTTAGGCCTTCGTGATGAGAAAATTTGGAGGCACTTTTACACAAGGTTCAGTTCAATCATATAACTGGATCCAATATATAATTCATCCAAAGTACAAAAGCTAACTCTCAAATTA

>K1247997-Seq200

ATCACTTCTTCTTCATCGGGACGGCGTGCCGACACATTATCTGGTGTAGTCGAGCCCATCGAACTCATCTTCCCTCTTGAAATCAGAAGGTACCTTCGCGCGATCATCATCCATCCAAATCCTTCTTGTTATGTGCTTGATTGAACTCAATTTTGTTCTCATTTGATGTACGTATGTTTACTATTTTCTGCTTTTTTTTTT

**Supplementary Table 15. Detailed information for MITE annotation in the 5-kb flanking regions of *S-RNase* alleles in citrus**

| **No.** | **Accession name** | **Catalog** | **Scientific name** | **Start-End** | **Length** | **Subfamily** | **Family** | **TIR/Location** | **TSD** | **RepeatMasker** | **MITE Hunter** | **Accession number** |
| --- | --- | --- | --- | --- | --- | --- | --- | --- | --- | --- | --- | --- |
| **1** | Abu-*S_12_*-F | Atalantia | *Atalantia buxifolia* | 9387-10187 | 801 | DTH | PIF | GGACCAGTTTAGGATTGCGG-  CCACAATCTCAAACTAGCCC /9387-10168 | ATA | 9387-9677;  9742-10182 |  | OQ672683 |
| **2** | Ptr-*S_10_*-F | Trifoliate orange | *Poncirus trifoliata* | 2262-3064 | 803 | DTM | Mutator | TCAACATTAG-  CTAATGTTGA/2262-3055 | TA | 2195-2430;  3008-3133 | 2202-3124;  2312-2603 | OQ672684 |
| **3** | Ptr-*S_10_*-F | Trifoliate orange | *Poncirus trifoliata* | 9135-9452 | 318 | DTM | Mutator | CCGCGA-  TTGCGG/9135-9447 | AT | 9125-9410 |  | OQ672684 |
| **4** | Cre-*S_10_*-F | Mandarin | *Citrus reticulata* | 4512-4579 | 68 | DTA | hAT | TTTCCC-  GGGGAA/4512-4574 | AAT | 4435-4624 | 4339-4684 | OQ672685 |
| **5** | Cre-*S_10_*-F | Mandarin | *Citrus reticulata* | 3809-4017 | 209 | DTA | hAT | TTTTGAGT-  ATTCAAAA/3809-4010 | TTA | 3651-4176 |  | OQ672685 |
| **6** | Cgr-*S_3_*-F | Pummelo | *Citrus maxima* | 6086-6705 | 620 | DTH | PIF | GGTAGCGTTT-  AAACGCCACC/6086-6696 | TAA | 6086-6706 | 6026-6765 | OQ672686 |
| **7** | Ptr-*S_30_*-F | Trifoliate orange | *Poncirus trifoliata* | 4004-4369 | 366 | DTH | PIF | TACCCTTATT-  AATAAAGGTA/4004-4360 | AAA | 4288-4476 | 3944-4429 | OQ672687 |
| **8** | Cme-*S_30_*-F | Citron | *Citrus medica* | 3948-4311 | 364 | DTH | PIF | TACCCTTATT-  AATAAAGGTA/3948-4302 | AAA | 4232-4419 | 3888-4371 | OQ672688 |
| **9** | Cma-*S_23_*-F | Wild mandarin | *Citrus mangshanensis* | 9604-10313 | 710 | DTH | PIF | GCAATATGAG-  CTCAATTC/9604-10306 | TAAAT | 9614-9673;  9970-10325 | 9425-10411 | OQ672689 |
| **10** | Cme-*S_21_*-F | Citron | *Citrus medica* | 8711-9103 | 369 | DTM | Mutator | TTTTAAAA-  TAAAGAAA/8711-9096 | TA | 8719-9086 |  | OQ672690 |
| **11** | Cme-*S_21_*-F | Citron | *Citrus medica* | 4386-4682 | 298 | DTM | Mutator | AATTTGTAGC-  GCTACACATT/4446-4613 | TA | 4328-4623 | 4386-4682 | OQ672690 |
| **12** | Cgr-*S_17_*-F | Pummelo | *Citrus maxima* | 4602-4775 | 174 | DTH | PIF | AAGAGAAAATAA-  TTATTTAACTCTT/4602-4763 | TTA |  | 3890-4666 | OQ672691 |
| **13** | Cho-*S_17_*-F | Honghe papeda | *Citrus hongheensis* | 4602-4775 | 174 | DTH | PIF | AAGAGAAAATAA-  TTATTTAACTATT/4602-4763 | TTA |  | 3815-4666 | OQ672692 |
| **14** | Fhi-*S_19_*-F | Hongkong kumquat | *Fortunella hindsii* | 8082-8819 | 738 | DTM | Mutator | TGTTCATCCA-  TGAATGAACA/8082-8810 | TA |  | 8022-8879 | OQ672693 |
| **15** | Fhi-*S_29_*-F | Hongkong kumquat | *Fortunella hindsii* | 7061-7835 | 775 | DTM | Mutator | CCTAGAGGTGTTTAG-  CTCAATATACCCTTGT/7061-7820 | TA |  | 7181-7921 | OQ672695 |
| **16** | Fhi-*S_29_*-F | Hongkong kumquat | *Fortunella hindsii* | 8065-8616 | 552 | DTM | Mutator | GTGTTTGGTA-  TACCAAATAC/8065-8607 | TA | 7979-8679 | 8005-8676 | OQ672695 |
| **17** | Fhi-*S_2_*-F | Hongkong kumquat | *Fortunella hindsii* | 3152-3929 | 778 | DTA | hAT | CAGGGCCGGCTTTAG-  CTAAAGCCACCCCTG/3152-3915 | AATGGTTC |  |  | OQ672697 |
| **18** | Cgr-*S_2_*-F | Pummelo | *Citrus maxima* | 6698-7178 | 481 | DTH | PIF | GTTAGAGATA-  AATCTCTAAC/6698-7169 | TTT | 6793-7068 |  | OQ672698 |
| **19** | Cre-*S_2_*-F | Mandarin | *Citrus reticulata* | 6666-7146 | 481 | DTH | PIF | GTTAGAGATA-  AATCTCTAAC/6666-7137 | TTT | 6761-7022 | 6606-7206 | OQ672699 |
| **20** | Cre-*S_2_*-F | Mandarin | *Citrus reticulata* | 7797-8100 | 304 | DTH | PIF | TGCGAGGTAT-  ATTGCTCCT/7797-8092 | TTA | 7834-8071 |  | OQ672699 |
| **21** | Cma-*S_2_*-F | Wild mandarin | *Citrus mangshanensis* | 6667-7147 | 481 | DTH | PIF | GTTAGAGATA-  AATCTCTAAC/6667-7138 | TTT | 6763-7023 | 6607-7207 | OQ672700 |
| **22** | Cma-*S_2_*-F | Wild mandarin | *Citrus mangshanensis* | 7812-8089 | 278 | DTH | PIF | TGCGAGGTAT-  ATTGCTCCT/7812-8081 | TTA | 7871-8060 |  | OQ672700 |
| **23** | Cme-*S_14_*-F | Citron | *Citrus medica* | 1734-2329 | 596 | DTM | Mutator | TCTTATCTGA-  TTAGATAAGA/1734-2320 | TA |  | 1674-2389 | OQ672701 |
| **24** | Cme-*S_14_*-F | Citron | *Citrus medica* | 4053-4695 | 643 | DTM | Mutator | TCAATTTACC-  GGTAAATTGA/4053-4686 | TA | 4031-4795 | 3993-4755 | OQ672701 |
| **25** | Cme-*S_14_*-F | Citron | *Citrus medica* | 6741-7693 | 953 | DTA | hAT | TAGGGATGGCAA-  TTGCCATCCCTA/6741-7682 | CACTGTTT | 6741-6923;  7090-7601 | 7359-7759 | OQ672701 |
| **26** | Cic-*S_14_*-F | Ichang papeda | *Citrus ichangensis* | 654-1421 | 768 | DTM | Mutator | AGGGAAATTA-  TAATTTCCCA/654-1412 | ATTAAAA | 655-1420 | 594-1481 | OQ672702 |
| **27** | Cic-*S_14_*-F | Ichang papeda | *Citrus ichangensis* | 4007-4737 | 731 | DTH | PIF | TTGCCGAAAA-  TTTTCAGCAA/4007-4728 | AGT | 3950-4795 | 3947-4797 | OQ672702 |
| **28** | Cic-*S_13_*-F | Ichang papeda | *Citrus ichangensis* | 10211-10349 | 139 | DTM | Mutator | GAATCG-  CGATTG/10211-10344 | GTG | 10079-10405 |  | OQ672703 |
| **29** | Cgr-*S_5_*-F | Pummelo | *Citrus maxima* | 1504-2194 | 691 | DTA | hAT | GGGAAATTTA-  TAAATTTCCC/1504-2185 | AAAAACTTA | 1504-2195 | 1520-2179 | OQ672704 |
| **30** | Cgr-*S_5_*-F | Pummelo | *Citrus maxima* | 10261-10497 | 237 | DTM | Mutator | TGGTCGGA-  TCCGACCG/10261-10490 | GGT | 10198-10522 |  | OQ672704 |
| **31** | Cau-*S_31_*-F | Australian limes | *Citrus australasica* | 9607-9764 | 158 | DTA | hAT | TTCCATTC-  GAATTGAA/9607-9757 | TTA | 9504-9797 |  | OQ672707 |
| **32** | Ptr-*S_31_*-F | Trifoliate orange | *Poncirus trifoliata* | 2738-3081 | 344 | DTA | hAT | CACTTT-  AAATTG/2738-3076 | ACCTTA |  |  | OQ672708 |
| **33** | Ptr-*S_31_*-F | Trifoliate orange | *Poncirus trifoliata* | 8226-8316 | 91 | DTA | hAT | ACATCATT-  AATGATCT/8226-8309 | CAAAC | 8155-8451 |  | OQ672708 |
| **34** | Cre-*S_31_*-F | Mandarin | *Citrus reticulata* | 2040-2184 | 137 | DTA | hAT | TAATAAT-  ATTAGTTA/2040-2181 | TAAT |  |  | OQ672709 |
| **35** | Cgr-*S_18_*-F | Pummelo | *Citrus maxima* | 7588-8055 | 468 | DTH | PIF | TATCAATGAG-  CTCATGGATA/7588-8046 | TAA |  | 7528-8115 | OQ672711 |
| **36** | Cgr-*S_6_*-F | Pummelo | *Citrus maxima* | 1308-1973 | 666 | DTA | hAT | TAGGGGTAAGTA-  TGCCCACCCCTA/1309-1962 | GTTTATT | 1309-1974 | 1308-1552 | OQ672712 |
| **37** | Csi-*S_m_*-F | Sweet orange | *Citrus sinensis* | 3010-3719 | 710 | DTM | Mutator | CAAGAGGTTTAATG-  CAATAGTCCCACTC/3010-3706 | TA | 3098-3459;  3540-3720 |  | OQ672715 |
| **38** | Cme-*S_8_*-F | Citron | *Citrus medica* | - | - | - | - | - | - | - | - | OQ672680 |
| **39** | Cau-*S_8_*-F | Australian limes | *Citrus australasica* | - | - | - | - | - | - | - | - | OQ672681 |
| **40** | Fhi-*S_8_*-F | Hongkong kumquat | *Fortunella hindsii* | - | - | - | - | - | - | - | - | OQ672682 |
| **41** | Cho-*S_19_*-F | Honghe papeda | *Citrus hongheensis* | - | - | - | - | - | - | - | - | OQ672694 |
| **42** | Ptr-*S_2_*-F | Trifoliate orange | *Poncirus trifoliata* | - | - | - | - | - | - | - | - | OQ672696 |
| **43** | Cgr-*S_22_*-F | Pummelo | *Citrus maxima* | - | - | - | - | - | - | - | - | OQ672705 |
| **44** | Abu-*S_22_*-F | Atalantia | *Atalantia buxifolia* | - | - | - | - | - | - | - | - | OQ672706 |
| **45** | Cle-*S_11_*-F | Clementine | *Citrus clementina* | - | - | - | - | - | - | - | - | OQ672710 |
| **46** | Cun-*S_9_*-F | Satsuma | *Citrus unshiu* | - | - | - | - | - | - | - | - | OQ672713 |
| **47** | Cic-*S_26_*-F | Ichang papeda | *Citrus ichangensis* | - | - | - | - | - | - | - | - | OQ672714 |
| **48** | Cgr-*S_1_*-F | Pummelo | *Citrus maxima* | - | - | - | - | - | - | - | - | OQ672716 |

**Supplementary Table 16. Detailed information for MITE annotation near the *S-RNase* genes in three common GSI families**

| **No.** | **Accession name** | **Catalog** | **Scientific name** | **SI/SC** | **Start-End** | **Length** | **Subfamily** | **Family** | **TIR** | **TSD** | **Accession number** | ***S*-RNase accession** |
| --- | --- | --- | --- | --- | --- | --- | --- | --- | --- | --- | --- | --- |
| **1** | Pce-*S_6m_*-F | Sour cherry | *Prunus cerasus* | SC ^16^ | 3220-3750 | 531 | DTA | hAT | CTCTCTCTCA-AGAGAGAGAG | CGA | OR359651 | ABD49100.1 |
| **2** | Pav-*S_6_*-F | Sweet cherry | *Prunus avium* | SI ^16,17^ | - | - | - | - | - | - | OR359652 | XP 021802050.1 |
| **3** | Ppe-*S*-F | Wild peach | *Prunus persica* | SC ^18^ | 3814-3996 | 183 | DTM | Mutator | AGGATGAATACTTTG-CAAATTATATTACGTG | AT | OR359654 | XP 007207578.1 |
| **4** | Ppe-*S*-F | Wild peach | *Prunus persica* | SC ^18^ | 4048-4232 | 185 | DTM | Mutator | TTGGCTCCTTAA-TTAAGGAGCCAA | AC | OR359654 | XP 007207578.1 |
| **5** | Ppe-*S*-F | Wild peach | *Prunus persica* | SC ^18^ | 4664-4840 | 177 | DTM | Mutator | TTTTGGAAAAA-TTTTTCCAAAAA | TGA | OR359654 | XP 007207578.1 |
| **6** | Pmu-*S*-F | Japanese apricot | *Prunus mume* | SI ^19^ | 9570-10083 | 514 | DTA | hAT | CTTCACAATA-TTTCTTGAAG | CT | OR359653 | XP_008245498.1 |
| **7** | Par-*S_4_*-F | Apricot | *Prunus armeniaca* | SI ^19-21^ | 1541-2093 | 553 | DTH | PIF | TTTTGTTTTTTT-AAAAGGACAAAGA | CATTT | OR359648 | AAT69248.1 |
| **8** | Pav-*S_3_*-F | Sweet cherry | *Prunus avium* | SI ^17,22^ | 1570-2178 | 609 | DTM | Mutator | AGAGATTTTCTATTTAAA-TTTAAATAGAAAATCTCT | TTAATT | OR359658 | XP_021800842.1 |
| **9** | Pav-*S_3_*-F | Sweet cherry | *Prunus avium* | SI ^17^ | 3977-4312 | 336 | DTH | PIF | TTTGGATGA-TCCTCCAAA | TG | OR359658 | XP_021800842.1 |
| **10** | Eja-*S_6_*-F | Loquat | *Eriobotrya japonica* | SC ^23^ | 5845-5932 | 88 | DTA | hAT | CTAGTGTGTGTGT-ATATATATATAAG | TAG | OR400937 | GQ202269.4 |
| **11** | Eja-*S_6_*-F | Loquat | *Eriobotrya japonica* | SC ^23^ | 6098-6326 | 229 | DTM | Mutator | GGGTTTTTAT-AAAAAAACCC | TAAAA | OR400937 | GQ202269.4 |
| **12** | Pbr-*S_27_*-F | Chinese white pear | *Pyrus bretschneideri* | SI ^24^ | - | - | - | - | - | - | OR359650 | O80325.1 |
| **13** | Eja-*S_2_*-F | Loquat | *Eriobotrya japonica* | SI ^25,26^ | 10372-10659 | 288 | DTA | hAT | GATCCCAAAC-GTTTGGGATC | GGTTCGGTATGG | OR359645 | EVM0013706.1 |
| **14** | Mdo-*S_3_*-F | Apple | *Malus domestica* | SI ^27^ | 7722-8080 | 359 | DTM | Mutator | TTTTTATCCAAAAT-ATTTTGGCTAAAAA | AAC | OR359646 | XP 028953775.1 |
| **15** | Ahi-*S_7_*-F | Snapdragon | *Antirrhinum hispanicum* | SI ^28^ | - | - | - | - | - | - | OR359640 | OR359659.1 |
| **16** | Ahi-*S_8_*-F | Snapdragon | *Antirrhinum hispanicum* | SI ^28^ | - | - | - | - | - | - | OR359641 | OR359660.1 |
| **17** | Stu-*S_8_*-F | Potato (A6-26) | *Solanum tuberosum* | SC ^29^ | 7053-7314 | 262 | DTT | Mariner | CTCTCTCTGTCCA-TGAACGGAAGAAG | TA | OR359639 | MZ561411.1 |
| **18** | Pax-*S_1_*-F | Large white petunia | *Petunia axillaris* | SI ^30,31^ | 6317-6458 | 142 | DTA | hAT | ATATATATATA-TGTGTATATGT | TAAT | OR359649 | AAA60465.1 |
| **19** | Stu-*S_4_*-F | Potato (E4-63) | *Solanum tuberosum* | SC ^29^ | 4538-4826 | 289 | DTH | PIF | TAAAAGTTAGAG-CTATTTTTTTTAG | CCA | OR359644 | MZ561407.1 |
| **20** | Sly-*S_3_*-F | Cultivated tomato | *Solanum lycopersicum* | SC ^32^ | 4161-4390 | 230 | DTT | Mariner | AATAATTACTTTC-GAAAGTACATTATT | CATT | OR359656 | XP 004229063.1 |
| **21** | Sly-*S_3_*-F | Cultivated tomato | *Solanum lycopersicum* | SC ^32^ | 4545-4832 | 288 | DTH | PIF | TAAAAGTTAGA-TCTTTTTAGC | CA | OR359656 | XP 004229063.1 |
| **22** | Sly-*S_3_*-F | Cultivated tomato | *Solanum lycopersicum* | SC ^32^ | 6238-6543 | 306 | DTM | Mutator | GAGAAATTGC-GCATTTTCTC | TA | OR359656 | XP 004229063.1 |
| **23** | Sch-*S_2_*-F | Wild potato (M6) | *Solanum chacoense* | SC ^33^ | 6150-6567 | 418 | DTA | hAT | CAGTGGTGGATCCA-TGGGTCCGCCCCTG | TAT | OR359655 | MZ561405.1 |
| **24** | Stu-*S_3_*-F | Potato (DM) | *Solanum tuberosum* | SI ^34,35^ | 236-1080 | 845 | DTM | Mutator | AGAGAAAAGACATAAAGAC-GTCTTTATGTCTTTTCTCT | AATATAAT | OR359657 | MZ561406.1 |
| **25** | Can-*S*-F | Pepper | *Capsicum annuum* | SC ^36,37^ | - | - | - | - | - | - | OR359642 | XP 047269761.1 |
| **26** | Nta-*S*-F | Tobacco TN90 | *Nicotiana tabacum* | SC ^38^ | 3333-4018 | 686 | DTH | PIF | AAATGGCGAA-TTCGACATTT | CAA | OR400938 | OR359661.1 |

**Supplementary Table 17. List of primers used in this study**

| **Name** | **Direction** | **Primer sequence (5’ to 3’)** | **Purpose** |
| --- | --- | --- | --- |
| *Actin*_F | Forward | CCGACCGTATGAGCAAGGAAA | SqRT-PCR of *Actin* |
| *Actin*_R | Reverse | TTCCTGTGGACAATGGATGGA |  |
| *S_1_-RNase*-SpeA_F | Forward | CACTTTTGGCTGGTTCAGAGC | Specific amplification of *S_1_-RNase* |
| *S_1_-RNase*-SpeA_R | Reverse | AGTCTGATAGCCGCTTGGAG |  |
| *S_2_-RNase*-SpeA_F | Forward | CGCTGGGGGAAAAACATTGG | Specific amplification of *S_2_-RNase* |
| *S_2_-RNase*-SpeA_R | Reverse | CGGTGAACCGACTCCGTAAT |  |
| *S_3_-RNase*-SpeA_F | Forward | GGCCTTTGGCCAGTAAATGC | Specific amplification of *S_3_-RNase* |
| *S_3_-RNase*-SpeA_R | Reverse | ATCGTAACCGGTTTTGGCCT |  |
| *S_4_-RNase*-SpeA_F | Forward | TCCTCAGGTGCAGCACAAAA | Specific amplification of *S_4_-RNase* |
| *S_4_-RNase*-SpeA_R | Reverse | TAGCCCTTCGTAAGGTTGGC |  |
| *S_5_-RNase*-SpeA_F | Forward | GCCTCTGGCCAGTAACCTTT | Specific amplification of *S_5_-RNase* |
| *S_5_-RNase*-SpeA_R | Reverse | AGCCTTTATCGCGTCCTTGT |  |
| *S_6_-RNase*-SpeA_F | Forward | GCAATAACCCGCCATTCGAC | Specific amplification of *S_6_-RNase* |
| *S_6_-RNase*-SpeA_R | Reverse | CGCACGTAGTTGATCCCCTT |  |
| *S_7_-RNase*-SpeA_F | Forward | GTGGACCGCAATGGAACAAG | Specific amplification of *S_7_-RNase* |
| *S_7_-RNase*-SpeA_R | Reverse | ATGACCTCCAATGCGCTCTT |  |
| *S_8_-RNase*-SpeA_F | Forward | GTTCGTCATACACGGCCTCT | Specific amplification of *S_8_-RNase* |
| *S_8_-RNase*-SpeA_R | Reverse | CATGATTGTGTCACGGCAGC |  |
| *S_9_-RNase*-SpeA_F | Forward | TCGACTTCGTCCTACATGGC | Specific amplification of *S_9_-RNase* |
| *S_9_-RNase*-SpeA_R | Reverse | CCTCAGTCTGATAGCCGCTT |  |
| *S_10_-RNase*-SpeA_F | Forward | AAGTCAGATGCGCCCGTAAT | Specific amplification of *S_10_-RNase* |
| *S_10_-RNase*-SpeA_R | Reverse | CATGATTGTGTCACGGCAGC |  |
| *S_11_-RNase*-SpeA_F | Forward | TCGTGTTACATGGCCTCTGG | Specific amplification of *S_11_-RNase* |
| *S_11_-RNase*-SpeA_R | Reverse | TTGACCGGTGCCATGTTCTT |  |
| *S_12_-RNase*-SpeA_F | Forward | GCGCCCGAAATGTATCAAGG | Specific amplification of *S_12_-RNase* |
| *S_12_-RNase*-SpeA_R | Reverse | GCGCCCGAAATGTATCAAGG |  |
| *S_13_-RNase*-SpeA_F | Forward | GCCTCTGGCCAGTAACCTTT | Specific amplification of *S_13_-RNase* |
| *S_13_-RNase*-SpeA_R | Reverse | AGCCTTTATCGCGTCCTTGT |  |
| *S_14_-RNase*-SpeA_F | Forward | TGGCCATCTGGCTATTGCTC | Specific amplification of *S_14_-RNase* |
| *S_14_-RNase*-SpeA_R | Reverse | AGACCTGCACCTCTCAGTGT |  |
| *S_15_-RNase*-SpeA_F | Forward | CAGTGAACTCCACGGAGCAT | Specific amplification of *S_15_-RNase* |
| *S_15_-RNase*-SpeA_R | Reverse | TAGATCGACAGCCCTTCGGA |  |
| *S_16_-RNase*-SpeA_F | Forward | CCCTGATATCGACGGGATGG | Specific amplification of *S_16_-RNase* |
| *S_16_-RNase*-SpeA_R | Reverse | CTTGATCGTCAGCGCAAAGG |  |
| *S_17_-RNase*-SpeA_F | Forward | CCGTCTGGCTATTGCTTGGA | Specific amplification of *S_17_-RNase* |
| *S_17_-RNase*-SpeA_R | Reverse | TAGATCGACAGCCCTTCGGA |  |
| *S_18_-RNase*-SpeA_F | Forward | CCCCTGCAAAAATAACCCGC | Specific amplification of *S_18_-RNase* |
| *S_18_-RNase*-SpeA_R | Reverse | GGATCTAAAGGTTGGCCGCT |  |
| *S_19_-RNase*-SpeA_F | Forward | AATTGTTCGCGGACTAGCGA | Specific amplification of *S_19_-RNase* |
| *S_19_-RNase*-SpeA_R | Reverse | AGCCGAGAACTGGGATCTGA |  |
| *S_20_-RNase*-SpeA_F | Forward | GGGACAAACTCTGGAAGGCA | Specific amplification of *S_20_-RNase* |
| *S_20_-RNase*-SpeA_R | Reverse | CACCTCCTTTAGTGCCCGTT |  |
| *S_21_-RNase*-SpeA_F | Forward | GAACTCGACGGAGCAGACAT | Specific amplification of *S_21_-RNase* |
| *S_21_-RNase*-SpeA_R | Reverse | GCGGTTCGGGGAACTTGATA |  |
| *S_22_-RNase*-SpeA_F | Forward | GCCGTTGCATTTCGTCCTAC | Specific amplification of *S_22_-RNase* |
| *S_22_-RNase*-SpeA_R | Reverse | GATTTTGAGCGCTGCCATGT |  |
| *S_23_-RNase*-SpeA_F | Forward | CAGTGAACTCCACGGAGCAT | Specific amplification of *S_23_-RNase* |
| *S_23_-RNase*-SpeA_R | Reverse | ACTGCCTCCGTTTGCCAATA |  |
| *S_24_-RNase*-SpeA_F | Forward | CAGTAAGCTCCAGTGGGCAA | Specific amplification of *S_24_-RNase* |
| *S_24_-RNase*-SpeA_R | Reverse | CACCCTACAGCTCCCCCTAT |  |
| *S_25_-RNase*-SpeA_F | Forward | GCAGACCACTGTTGGCTAGT | Specific amplification of *S_25_-RNase* |
| *S_25_-RNase*-SpeA_R | Reverse | GGCTCTACACCTTCGGCTTT |  |
| *S_26_-RNase*-SpeA_F | Forward | TTTCCTGCATTGCCTCAGGT | Specific amplification of *S_26_-RNase* |
| *S_26_-RNase*-SpeA_R | Reverse | AGGCACCCTGTCCTTCTGTA |  |
| *S_27_-RNase*-SpeA_F | Forward | CCTTTGGCCAGTGAACTCCA | Specific amplification of *S_27_-RNase* |
| *S_27_-RNase*-SpeA_R | Reverse | GATCGACAGCCCTTCGGAAA |  |
| *S_28_-RNase*-SpeA_F | Forward | TTGGCTAGTTCTGGTGTGGC | Specific amplification of *S_28_-RNase* |
| *S_28_-RNase*-SpeA_R | Reverse | CGGGATCAACGCACAAAGTC |  |
| *S_29_-RNase*-SpeA_F | Forward | TTGGCTAGTTCAGGTGTGGC | Specific amplification of *S_29_-RNase* |
| *S_29_-RNase*-SpeA_R | Reverse | TTAGTTCGACCGCTCTTCGG |  |
| *S_30_-RNase*-SpeA_F | Forward | TGCTTTGCTTGTAGCCAACG | Specific amplification of *S_30_-RNase* |
| *S_30_-RNase*-SpeA_R | Reverse | AGCACTACCATGGATTCGCC |  |
| *S_31_-RNase*-SpeA_F | Forward | GCAGCTCAAAACTCTTCGGG | Specific amplification of *S_31_-RNase* |
| *S_31_-RNase*-SpeA_R | Reverse | AGCGCTACCATGTTTTTGCC |  |
| *S_32_-RNase*-SpeA_F | Forward | CAACGGTGCAGCTCAATTCG | Specific amplification of *S_32_-RNase* |
| *S_32_-RNase*-SpeA_R | Reverse | GTACTGGAAGACGACCGGTTT |  |
| *S_33_-RNase*-SpeA_F | Forward | AAGATTCTGCAAACCACGGC | Specific amplification of *S_33_-RNase* |
| *S_33_-RNase*-SpeA_R | Reverse | CTAGGCTCTAAACCTGCGCC |  |
| *S_34_-RNase*-SpeA_F | Forward | GAAGTCAGATGCGCCCGTAA | Specific amplification of *S_34_-RNase* |
| *S_34_-RNase*-SpeA_R | Reverse | CCACCTGTCGTTGAGCACTA |  |
| *S_35_-RNase*-SpeA_F | Forward | AGCTGGCCACCTGTCTATTG | Specific amplification of *S_35_-RNase* |
| *S_35_-RNase*-SpeA_R | Reverse | TGTGTCCTGGTAAGGTTGGC |  |
| *FhiS_2_-RNase*-Ful_F | Forward | ATGAAGGCGACCAACCTCTTTTGC | gene clone and vector construction |
| *FhiS_2_-RNase*-Ful_R | Reverse | TTAAAAAGAATTAAGATGGATGTC |  |
| *FhiS_2_-RNase*-qRT_F | Forward | GGCCACATGGCTATTGCTTG | Quantitative real time polymerase chain reaction (qRT-PCR) of *S_2_-RNase* |
| *FhiS_2_-RNase*-qRT_R | Reverse | GTGCTCCTAAGGTCCGTTGT |  |
| *FhiS_2_-RNase*-SqRT_F | Forward | CACAGCTCAAAACACCTCGC | Semi-quantitative polymerase chain reaction (SqRT-PCR) of *S_2_-RNase* |
| *FhiS_2_-RNase*-SqRT_R | Reverse | GGCCAAAGCCCATGTAGGAT |  |
| *FhiS_8_-RNase*-SqRT_F | Forward | GTTCGTCATACACGGCCTCT | SqRT-PCR of *S_8_-RNase* |
| *FhiS_8_-RNase*-SqRT_R | Reverse | CACCACCTGTTGTTGAGCAC |  |
| *FhiS_19_-RNase*-SqRT_F | Forward | GGCCACATGGCTATTGCTTG | SqRT-PCR of *S_19_-RNase* |
| *FhiS_19_-RNase*-SqRT_R | Reverse | ACAAGTGGACTCTTGATGCGA |  |
| *FhiS_29_-RNase*-SqRT_F | Forward | TCATCCTACACGGCCTTTGG | SqRT-PCR) of *S_29_-RNase* |
| *FhiS_29_-RNase*-SqRT_R | Reverse | TTAGTTCGACCGCTCTTCGG |  |
| Fhi_p*S_29_*-Frg1_F | Forward | AACCCTTCTCCATGTTCCC | Fh_*pS_29_*::g*S_2_* clone and vector construction |
| Fhi_p*S_29_*-g*S_2_*-overlap_R | Reverse | AAGAGGTTGGTCGCCTTCATCTTCATGTTTGTCAATATAGGTTC |  |
| Fhi_g*S_2_*-p*S_29_*-overlap_F | Forward | GAACCTATATTGACAAACATGAAGATGAAGGCGACCAACCTCTT |  |
| Fhi_g*S_2_*-Frg2_R | Reverse | TTAAAAAGAATTAAGATGGATGTCT |  |
| Fhi_p*S_2_*-Frg1-F | Forward | CACGGGTGTCACCATCAAGAG | Fh_*pS_2_*-∆MITE promoter clone and vector construction |
| Fhi_p*S_2_*-Mite-Ovlp-Frg1-R | Reverse | GATCCAAATCGAACCATTAAGCAGTTGTTCTCATC |  |
| Fhi_p*S_2_*-Mite-Ovlp-Frg2-F | Forward | TTAATGGTTCGATTTGGATCTTAGCTAAAATCCCG |  |
| Fhi_p*S_2_*-Frg2-R | Reverse | CTTTGCTTTTTTCGATATCGTTTTC |  |
| p*S_2_*_F | Forward | CACGGGTGTCACCATCAAGAG | Fh_*pS_2_*, Fh_*pS_2_*-∆MITE, Ptr_*pS_2_* promoter clone and vector construction |
| p*S_2_*_R | Reverse | CTTTGCTTTTTTCGATATCGTTTTC |  |
| Fhi_p*S_29_*_F | Forward | AACCCTTCTCCATGTTCCC | Fh_*pS_29_* promoter clone and vector construction |
| Fhi_p*S_29_*_R | Reverse | CTTCATGTTTGTCAATATAGGTTC |  |
| Fhi_p*S_8_*_F | Forward | GGGGCATGGCGGAAGTAA | Fh_*pS_8_* promoter clone and vector construction |
| Fhi_p*S_8_*_R | Reverse | ATTTGTGAATTCAAGTTCGTAGT |  |
| Fhi_p*S_19_*_F | Forward | GGAGGAGCAATTTGTGGAATC | Fh_*pS_19_* promoter clone and vector construction |
| Fhi_p*S_19_*_R | Reverse | CTTCATGTTTGTCAATGTAGGTTC |  |
| K48949-F1 | Forward | GAAGGTGACCAAGTTCATGCTGCTTCCGTTTACTTACCTTG | KASP Genotyping Analysis |
| K48949-F2 | Forward | GAAGGTCGGAGTCAACGGATTGCTTCCGTTTACTTACCTTT |  |
| K48949-R | Reverse | GGCACTTGGTGAAAATCGGG |  |
| K110285-F1 | Forward | GAAGGTGACCAAGTTCATGCTAAGGAAGGAAGCTGAAAGA | KASP Genotyping Analysis |
| K110285-F2 | Forward | GAAGGTCGGAGTCAACGGATTAAGGAAGGAAGCTGAAAGC |  |
| K110285-R | Reverse | CCATCAAGATCAAGCCCACC |  |
| K701162-F1 | Forward | GAAGGTGACCAAGTTCATGCTGTGGCAGAGCAGTGTAATTA | KASP Genotyping Analysis |
| K701162-F2 | Forward | GAAGGTCGGAGTCAACGGATTGTGGCAGAGCAGTGTAATTG |  |
| K701162-R | Reverse | TGCGGAGTTCAAATAGCAGA |  |
| K729768-F1 | Forward | GAAGGTGACCAAGTTCATGCTGGAATAGATCGGTCACTTCG | KASP Genotyping Analysis |
| K729768-F2 | Forward | GAAGGTCGGAGTCAACGGATTGGAATAGATCGGTCACTTCC |  |
| K729768-R | Reverse | GCTCTGGCTTCTTGTGTTGT |  |
| K812738-F1 | Forward | GAAGGTGACCAAGTTCATGCTACAATAATGCACCCCCCTTT | KASP Genotyping Analysis |
| K812738-F2 | Forward | GAAGGTCGGAGTCAACGGATTACAATAATGCACCCCCCTTC |  |
| K812738-R | Reverse | GTGTAGTCATGTCAAGGTCA |  |
| K936336-F1 | Forward | GAAGGTGACCAAGTTCATGCTCATCAGCGGACGATCCATCT | KASP Genotyping Analysis |
| K936336-F2 | Forward | GAAGGTCGGAGTCAACGGATTCATCAGCGGACGATCCATCA |  |
| K936336-R | Reverse | GCTACTTGCTCGATCGGTGA |  |
| K961397-F1 | Forward | GAAGGTGACCAAGTTCATGCTAATATCTTAGGCCTTCGTGA | KASP Genotyping Analysis |
| K961397-F2 | Forward | GAAGGTCGGAGTCAACGGATTAATATCTTAGGCCTTCGTGG |  |
| K961397-R | Reverse | CCTTGTGTAAAAGTGCCTCC |  |
| K982090-F1 | Forward | GAAGGTGACCAAGTTCATGCTGTCGGGGGTGTGTTAGGATG | KASP Genotyping Analysis |
| K982090-F2 | Forward | GAAGGTCGGAGTCAACGGATTGTCGGGGGTGTGTTAGGATA |  |
| K982090-R | Reverse | GAGAAAATGGATAGACACCCC |  |
| K1200030-F1 | Forward | GAAGGTGACCAAGTTCATGCTAACCTGATAGGCCAACAGCC | KASP Genotyping Analysis |
| K1200030-F2 | Forward | GAAGGTCGGAGTCAACGGATTAACCTGATAGGCCAACAGCT |  |
| K1200030-R | Reverse | TGCATGGGTTGGTCATTTGC |  |
| K1247997-F1 | Forward | GAAGGTGACCAAGTTCATGCTATCAGAAGGTACCTTCGCGC | KASP Genotyping Analysis |
| K1247997-F2 | Forward | GAAGGTCGGAGTCAACGGATTATCAGAAGGTACCTTCGCGT |  |
| K1247997-R | Reverse | AGCACATAACAAGAAGGATTTGG |  |
| K1270101-F1 | Forward | GAAGGTGACCAAGTTCATGCTCAGGCTGGAACAACCCTAAA | KASP Genotyping Analysis |
| K1270101-F2 | Forward | GAAGGTCGGAGTCAACGGATTCAGGCTGGAACAACCCTAAC |  |
| K1270101-R | Reverse | TTAATCGCTCGGTCGCTTCT |  |
| K1314434-F1 | Forward | GAAGGTGACCAAGTTCATGCTGAAAAGGCCTTTGAATGGGGT | KASP Genotyping Analysis |
| K1314434-F2 | Forward | GAAGGTCGGAGTCAACGGATTGAAAAGGCCTTTGAATGGGGG |  |
| K1314434-R | Reverse | TGGACGACTTGGGTTTTGGC |  |
| K1372852-F1 | Forward | GAAGGTGACCAAGTTCATGCTCATGCGCAGGTTAGAGAGGC | KASP Genotyping Analysis |
| K1372852-F2 | Forward | GAAGGTCGGAGTCAACGGATTCATGCGCAGGTTAGAGAGGT |  |
| K1372852-R | Reverse | TCAGGCTACCTCGGCTCCAG |  |
| K1512779-F1 | Forward | GAAGGTGACCAAGTTCATGCTGGACATTAATTAATTAGTTGGTTCG | KASP Genotyping Analysis |
| K1512779-F2 | Forward | GAAGGTCGGAGTCAACGGATTGGACATTAATTAATTAGTTGGTTCA |  |
| K1512779-R | Reverse | TACCTAAATCCACCTTCACT |  |
| K1850875-F1 | Forward | GAAGGTGACCAAGTTCATGCTCTAATGTCGCTCCTAAAACA | KASP Genotyping Analysis |
| K1850875-F2 | Forward | GAAGGTCGGAGTCAACGGATTCTAATGTCGCTCCTAAAACG |  |
| K1850875-R | Reverse | ACCTCAGATGCTGTGGTTCG |  |
| K2206344-F1 | Forward | GAAGGTGACCAAGTTCATGCTGACGTTCCTCAGACCTTTCT | KASP Genotyping Analysis |
| K2206344-F2 | Forward | GAAGGTCGGAGTCAACGGATTGACGTTCCTCAGACCTTTCG |  |
| K2206344-R | Reverse | CATCCCCACCAATCGTCAGA |  |
| Fhi_p*S_29_* :: *FhiS_2_-RNase*_F | Forward | GAAGGTGGGAAGGAGAGCAT | Positive identification of *FhiS_29_-RNase_pro_* :: *FhiS_2_-RNase* transgenic lines |
| Fhi_p*S_29_* ::*FhiS_2_-RNase*_R | Reverse | GTGCTCCTAAGGTCCGTTGT |  |
| p35*S* :: *FhiS_2_-RNase*_F | Forward | GACGTAAGGGATGACGCACA | Positive identification of 35*S_pro_* :: *FhiS_2_-RNase* transgenic lines |
| p35*S* :: *FhiS_2_-RNase*_R | Reverse | GCAACACTCCCTTCTCTGCT |  |
| Fhi_p*S_2∆MITE_* :: *FhiS2-RNase*_F | Forward | ATGCGTAGGTCTCCAAGCAA | Positive identification of *FhiS_2_-RNase_pro∆MITE_* :: *FhiS_2_-RNase* transgenic lines |
| Fhi_p*S_2∆MITE_* :: *FhiS2-RNase*_R | Reverse | GTAACGCCAGGGTTTTCCCA |  |
| Fhi_p*S_2_* :: *FhiS_2_-RNase*_F | Forward | ATGAGCCCTTAGCGTGCTTG | *FhS_2_* haplotype identification |
| Fhi_p*S_2_* :: *FhiS_2_-RNase*_R | Reverse | GTGCTCCTAAGGTCCGTTGT |  |
| p35*S*-F | Forward | TGAGACTTTTCAACAAAGGGTAA | p*35S* promoter clone and vector construction |
| p35*S*-R | Reverse | TGTCCTCTCCAAATGAAATGAACT |  |
| Cgr_p*S_2_*_F | Forward | GAAAAATGTTAAAATCCATATTAAA | *CgS_2_-RNase* promoter clone and vector construction |
| Cgr_p*S_2_*_R | Reverse | TTTCGTCTTTGTCGATATCA |  |
| Prt_pS2-Frg1_F | Forward | CACGGGTGTCACCATCAAG | *PtrS_2_-RNase_pro+MITE_* clone and vector construction |
| Prt_p*S_2_*-Frg1-Ovlap1_R | Reverse | GCCGGCCCTGGAACCATTAAGTAGTTGTTC |  |
| Prt_p*S_2_*+mite-Ovlap2_F | Forward | TTAATGGTTCCAGGGCCGGCTTTAGAGTCT |  |
| Prt_p*S_2_*+mite-Ovlap2_R | Reverse | AGACTCTAAAGCCGGCCCTGGAACCATTAA |  |
| Prt_p*S_2_*-Frg2-Ovlap3_F | Forward | TGAATGGTTCGATTTGGATCTTAGCTAAAA |  |
| Prt_p*S_2_*-Frg2_R | Reverse | CTTTGCTTTTTTCGATATCGTTTTC |  |
| Fhi_p*S_2_*-Frg1_F | Forward | CACGGGTGTCACCATCAAGAG | *FhS_2_-RNase_pro∆MITE_* :: *FhS_2_-RNase* clone and vector construction |
| Fhi_p*S_2_*-∆mite-Ovlap_R | Reverse | GGTCGCCTTCATCTTTGCTTTTTTCGATATCGTTTTC |  |
| Fhi_g*S_2_*-Ovlap_F | Forward | CGAAAAAAGCAAAGATGAAGGCGACCAACCTCTT |  |
| Fhi_g*S_2_*-Frg2_R | Reverse | CGAGGAAGTAACGAATTCAGTGG |  |

**Supplementary Table 18. Isolation and identification of *S*-ribonuclease alleles in citrus**

| **No.** | **Gene** | **Organism** | **Full length (bp)** | **Amino acid (aa)** | **Molecular mass (kDa)** | **Isoelectric point** | **Source** | **Accession number** |
| --- | --- | --- | --- | --- | --- | --- | --- | --- |
| 1 | *S_1_-RNase* | *Citrus maxima* | 669 | 222 | 23.38 | 8.23 | Liang et al., 2020 ^3^ | MN652897 |
| 2 | *S_2_-RNase* | *Citrus maxima* | 699 | 232 | 24.17 | 9.22 | Liang et al., 2020 ^3^ | MN652898 |
| 3 | *S_3_-RNase* | *Citrus maxima* | 696 | 231 | 24.06 | 9.11 | Liang et al., 2020 ^3^ | MN652899 |
| 4 | *S_4_-RNase* | *Citrus maxima* | 675 | 224 | 23.16 | 8.84 | Liang et al., 2020 ^3^ | MN652900 |
| 5 | *S_5_-RNase* | *Citrus maxima* | 660 | 219 | 23.16 | 7.71 | Liang et al., 2020 ^3^ | MN652901 |
| 6 | *S_6_-RNase* | *Citrus maxima* | 660 | 219 | 22.96 | 9.39 | Liang et al., 2020 ^3^ | MN652902 |
| 7 | *S_7_-RNase* | *Citrus maxima* | 696 | 231 | 24.02 | 8.93 | Liang et al., 2020 ^3^ | MN652903 |
| 8 | *S_8_-RNase* | *Citrus maxima* | 690 | 229 | 24.47 | 7.67 | Liang et al., 2020 ^3^ | MN652904 |
| 9 | *S_9_-RNase* | *Citrus maxima* | 690 | 229 | 24.1 | 9.33 | Liang et al., 2020 ^3^ | MN652905 |
| 10 | *S_10_-RNase* | *Citrus reticulata* | 690 | 229 | 24.02 | 9.12 | Liang et al., 2020 ^3^ | MN652906 |
| 11 | *S_11_-RNase* | *Citrus reticulata* | 690 | 229 | 24.08 | 9.47 | Liang et al., 2020 ^3^ | MN652907 |
| 12 | *S_12_-RNase* | *Atalantia buxifolia* | 690 | 229 | 24.25 | 9.30 | Liang et al., 2020 ^3^ | MN652908 |
| 13 | *S_13_-RNase* | *Citrus cavaleriei* | 660 | 219 | 23.22 | 7.71 | Liang et al., 2020 ^3^ | MN652909 |
| 14 | *S_14_-RNase* | *Citrus medica* | 678 | 225 | 22.91 | 8.27 | Liang et al., 2020 ^3^ | MN652910 |
| 15 | *S_15_-RNase* | *Citrus tamurana* | 678 | 225 | 25.6 | 9.31 | Honsho.C et al., 2021 ^39^ | LC575202 |
| 16 | *S_16_-RNase* | *Citrus maxima* | 660 | 219 | 25.07 | 8.42 | Honsho.C et al., 2021 ^39^ | LC575207 |
| 17 | *S_17_-RNase* | *Citrus hassaku* | 678 | 225 | 25.97 | 9.17 | Honsho.C et al., 2021 ^39^ | LC575209 |
| 18 | *S_18_-RNase* | *Citrus maxima* | 690 | 229 | 26.12 | 7.64 | In this study | ON227020 |
| 19 | *S_19_-RNase* | *Citrus maxima* | 687 | 228 | 26.49 | 9.42 | In this study | ON227021 |
| 20 | *S_20_-RNase* | *Citrus maxima* | 702 | 233 | 26.93 | 9.25 | In this study | ON227022 |
| 21 | *S_21_-RNase* | *Citrus maxima* | 678 | 225 | 26.05 | 9.21 | In this study | ON227023 |
| 22 | *S_22_-RNase* | *Citrus maxima* | 684 | 227 | 26.16 | 9.30 | In this study | ON227024 |
| 23 | *S_23_-RNase* | *Citrus maxima* | 678 | 225 | 25.69 | 9.29 | In this study | ON227025 |
| 24 | *S_24_-RNase* | *Citrus maxima* | 684 | 227 | 26.31 | 9.32 | In this study | ON227026 |
| 25 | *S_25_-RNase* | *Citrus maxima* | 660 | 219 | 25.25 | 8.51 | In this study | ON227027 |
| 26 | *S_26_-RNase* | *Citrus maxima* | 678 | 225 | 25.91 | 9.23 | In this study | ON227028 |
| 27 | *S_27_-RNase* | *Citrus maxima* | 678 | 225 | 25.85 | 9.31 | In this study | ON227029 |
| 28 | *S_28_-RNase* | *Fortunella hindsii* | 681 | 226 | 26.48 | 9.30 | In this study | ON227030 |
| 29 | *S_29_-RNase* | *Fortunella hindsii* | 681 | 226 | 26.43 | 9.46 | In this study | ON227031 |
| 30 | *S_30_-RNase* | *Poncirus trifoliata* | 693 | 230 | 26.56 | 9.41 | In this study | ON227032 |
| 31 | *S_31_-RNase* | *Citrus reticulata* | 696 | 231 | 26.45 | 8.63 | In this study | ON227033 |
| 32 | *S_32_-RNase* | *Fortunella hindsii* | 687 | 228 | 26.3 | 9.40 | In this study | ON227034 |
| 33 | *S_33_-RNase* | *Fortunella hindsii* | 672 | 223 | 25.16 | 8.96 | In this study | ON227035 |
| 34 | *S_34_-RNase* | *Fortunella hindsii* | 690 | 229 | 26.6 | 9.51 | In this study | ON227036 |
| 35 | *S_35_-RNase* | *Citrus medica* | 669 | 222 | 25.74 | 9.05 | In this study | ON227037 |
| 36 | *FhiS_2_-RNase* | *Fortunella hindsii* | 735 | 244 | 28.11 | 9.37 | In this study | ON227038 |
| 37 | *PtrS_2_-RNase* | *Poncirus trifoliata* | 699 | 232 | 26.62 | 9.23 | In this study | ON227039 |
| 38 | *AbuS_2_-RNase* | *Atalantia buxifolia* | 699 | 232 | 26.73 | 9.30 | In this study | ON227040 |
| 39 | *CmeS_2_-RNase* | *Citrus medica* | 699 | 232 | 26.65 | 9.24 | In this study | ON227041 |
| 40 | *CreS_2_-RNase* | *Citrus reticulata* | 699 | 232 | 26.46 | 9.29 | In this study | ON227042 |
| 41 | *PtrS_31_-RNase* | *Poncirus trifoliata* | 696 | 231 | 26.44 | 8.78 | In this study | ON227043 |
| 42 | *CicS_31_-RNase* | *Citrus ichangensis* | 696 | 231 | 26.45 | 8.63 | In this study | ON227044 |
| 43 | *CauS_31_-RNase* | *Citrus australasica* | 696 | 231 | 26.45 | 8.79 | In this study | ON227045 |
| 44 | *CauS_8_-RNase* | *Citrus australasica* | 690 | 229 | 26.68 | 9.44 | In this study | OQ656800 |
| 45 | *CmeS_8_-RNase* | *Citrus medica* | 690 | 229 | 26.68 | 9.44 | In this study | OQ656800 |
| 46 | *PtrS_10_-RNase* | *Poncirus trifoliata* | 690 | 229 | 26.63 | 9.20 | In this study | OQ656801 |
| 47 | *CleS_11_-RNase* | *Citrus clementina* | 690 | 229 | 24.08 | 9.47 | Liang et al., 2020 ^3^ | MN652907 |
| 48 | *CicS_14_-RNase* | *Citrus ichangensis* | 678 | 225 | 25.48 | 8.66 | In this study | OQ656802 |
| 49 | *CmeS_14_-RNase* | *Citrus medica* | 678 | 225 | 22.91 | 8.27 | Liang et al., 2020 ^3^ | MN652910 |
| 50 | *AbuS_22_-RNase* | *Atalantia buxifolia* | 684 | 227 | 26.14 | 9.44 | In this study | OQ656803 |
| 51 | *PtrS_30_-RNase* | *Poncirus trifoliata* | 693 | 230 | 26.57 | 9.41 | In this study | OQ656804 |
| 52 | *CreS_30_-RNase* | *Citrus reticulata* | 693 | 230 | 26.56 | 9.41 | In this study | ON227032 |

**Supplementary Table 19. Detailed information relating to checking the *S* alleles for 24 mandarin accessions**

| **No.** | **Accession name** | **Mapped *S-RNase* ^a^** | | **Catalog** | **Scientific** | **Source** | **SRR ID** |
| --- | --- | --- | --- | --- | --- | --- | --- |
| 1 | CZG | *S_11_* | *S_m_* | Cultivated mandarin | *Citrus reticulata* | Wang et al., 2017 ^8^ | SRR3747583 |
| 2 | CSNJ | *S_x_* | *S_m_* | Cultivated mandarin | *Citrus reticulata* | Wang et al., 2017 ^8^ | SRR3747609 |
| 3 | 19P | *S_2_* | *S_m_* | Cultivated mandarin | *Citrus reticulata* | Wang et al., 2017 ^8^ | SRR3747617 |
| 4 | 20H | *S_9_* | *S_m_* | Cultivated mandarin | *Citrus reticulata* | Wang et al., 2017 ^8^ | SRR3747635 |
| 5 | 18H | *S_7_* | *S_m_* | Cultivated mandarin | *Citrus reticulata* | Wang et al., 2017 ^8^ | SRR3749605 |
| 6 | HPJ | *S_28_* | *S_m_* | Cultivated mandarin | *Citrus reticulata* | Wang et al., 2017 ^8^ | SRR3750611 |
| 7 | YSJ | *S_30_* | *S_m_* | Cultivated mandarin | *Citrus reticulata* | Wang et al., 2017 ^8^ | SRR3750648 |
| 8 | NJ | *S_31_* | *S_m_* | Cultivated mandarin | *Citrus reticulata* | Wang et al., 2017 ^8^ | SRR3750668 |
| 9 | MLTJ | *S_30_* | *S_31_* | Cultivated mandarin | *Citrus reticulata* | Wang et al., 2017 ^8^ | SRR3750679 |
| 10 | MSJ | *S_30_* | *S_31_* | Cultivated mandarin | *Citrus reticulata* | Wang et al., 2017 ^8^ | SRR3751832 |
| 11 | LYJ | *S_7_* | *S_26_* | Cultivated mandarin | *Citrus reticulata* | Wang et al., 2017 ^8^ | SRR3756887 |
| 12 | BTJ | *S_2_* | *S_30_* | Cultivated mandarin | *Citrus reticulata* | Wang et al., 2017 ^8^ | SRR3756893 |
| 13 | STJ | *S_2_* | *S_30_* | Cultivated mandarin | *Citrus reticulata* | Wang et al., 2017 ^8^ | SRR3756933 |
| 14 | WLK | *S_11_* | *S_m_* | Cultivated mandarin | *Citrus reticulata* | Wang et al., 2017 ^8^ | SRR3820551 |
| 15 | ORI | *S_11_* | *S_m_* | Cultivated mandarin | *Citrus reticulata* | Wang et al., 2017 ^8^ | SRR3820595 |
| 16 | KYM | *S_7_* | *S_m_* | Cultivated mandarin | *Citrus reticulata* | Wang et al., 2017 ^8^ | SRR3820643 |
| 17 | QH117 | *S_7_* | *S_m_* | Cultivated mandarin | *Citrus reticulata* | Wang et al., 2017 ^8^ | SRR3822244 |
| 18 | NFJ | *S_7_* | *S_m_* | Cultivated mandarin | *Citrus reticulata* | Wang et al., 2017 ^8^ | SRR5796630 |
| 19 | WHPG | *S_2_* | *S_m_* | Cultivated mandarin | *Citrus reticulata* | Wang et al., 2017 ^8^ | SRR5796644 |
| 20 | JGA | *S_2_* | *S_m_* | Cultivated mandarin | *Citrus reticulata* | Wang et al., 2017 ^8^ | SRR5796822 |
| 21 | QTJ | *S_2_* | *S_x_* | Cultivated mandarin | *Citrus reticulata* | Wang et al., 2017 ^8^ | SRR5796863 |
| 22 | RT | *S_7_* | *S_26_* | Cultivated mandarin | *Citrus reticulata* | Wang et al., 2017 ^8^ | SRR5796864 |
| 23 | YJNJ | *S_x_* | *S_m_* | Cultivated mandarin | *Citrus reticulata* | Wang et al., 2017 ^8^ | SRR5796865 |
| 24 | ZHJ | *S_7_* | *S_26_* | Cultivated mandarin | *Citrus reticulata* | Wang et al., 2017 ^8^ | SRR5796927 |

Genome sequences of 24 mandarin accessions containing published sources were mapped to 36 *S-RNase* sequences (**Supplementary Table 18**). As a result, all accessions were found to contain these *S-RNase* alleles and 8/24 accessions do not contain the *S_m_-RNase* allele. In addition, 3/24 accessions contain the other SC allele, *S_31_-RNase*, and which of two do not contain the *S_m_-RNase* allele.

**a**: We assumed all accessions were heterozygous with two *S* alleles segregating. *S_x_* indicates the unmapped *S-RNase* allele.

**Reference:**

1 Wang, N. *et al.* Structural variation and parallel evolution of apomixis in citrus during domestication and diversification. *Natl Sci Rev* **9**, nwac114 (2022). <https://doi.org:10.1093/nsr/nwac114>

2 Huang, Y. *et al.* Genome of a citrus rootstock and global DNA demethylation caused by heterografting. *Hortic Res* **8**, 69 (2021). <https://doi.org:10.1038/s41438-021-00505-2>

3 Liang, M. *et al.* Evolution of self-compatibility by a mutant Sm-RNase in citrus. *Nat Plants* **6**, 131-142 (2020). <https://doi.org:10.1038/s41477-020-0597-3>

4 Hu, J. *et al.* Downregulated expression of S2-RNase attenuates self-incompatibility in "Guiyou No. 1" pummelo. *Hortic Res* **8**, 199 (2021). <https://doi.org:10.1038/s41438-021-00634-8>

5 Liu, H. *et al.* Citrus Pan-genome to Breeding Database (CPBD): A comprehensive genome database for citrus breeding. *Mol Plant* (2022). <https://doi.org:10.1016/j.molp.2022.08.006>

6 Wu, G. A. *et al.* Sequencing of diverse mandarin, pummelo and orange genomes reveals complex history of admixture during citrus domestication. *Nat Biotechnol* **32**, 656-662 (2014). <https://doi.org:10.1038/nbt.2906>

7 Zhu, C. *et al.* Genome sequencing and CRISPR/Cas9 gene editing of an early flowering Mini-Citrus (Fortunella hindsii). *Plant Biotechnol J* **17**, 2199-2210 (2019). <https://doi.org:10.1111/pbi.13132>

8 Wang, X. *et al.* Genomic analyses of primitive, wild and cultivated citrus provide insights into asexual reproduction. *Nat Genet* **49**, 765-772 (2017). <https://doi.org:10.1038/ng.3839>

9 Wang, L. *et al.* Genome of Wild Mandarin and Domestication History of Mandarin. *Mol Plant* **11**, 1024-1037 (2018). <https://doi.org:10.1016/j.molp.2018.06.001>

10 Xu, Q. *et al.* The draft genome of sweet orange (Citrus sinensis). *Nat Genet* **45**, 59-66 (2013). <https://doi.org:10.1038/ng.2472>

11 Wang, L. *et al.* Somatic variations led to the selection of acidic and acidless orange cultivars. *Nat Plants* **7**, 954-965 (2021). <https://doi.org:10.1038/s41477-021-00941-x>

12 Lu, Z. *et al.* The high-quality genome of pummelo provides insights into the tissue-specific regulation of citric acid and anthocyanin during domestication. *Hortic Res* **9**, uhac175 (2022). <https://doi.org:10.1093/hr/uhac175>

13 Shimizu, T. *et al.* Draft Sequencing of the Heterozygous Diploid Genome of Satsuma (Citrus unshiu Marc.) Using a Hybrid Assembly Approach. *Front Genet* **8**, 180 (2017). <https://doi.org:10.3389/fgene.2017.00180>

14 Kawahara, Y. *et al.* Mikan Genome Database (MiGD): integrated database of genome annotation, genomic diversity, and CAPS marker information for mandarin molecular breeding. *Breeding Science* **70**, 200-211 (2020). <https://doi.org:10.1270/jsbbs.19097>

15 Zheng, W. *et al.* Evolution-guided multiomics provide insights into the strengthening of bioactive flavone biosynthesis in medicinal pummelo. *Plant Biotechnol J* (2023). <https://doi.org:10.1111/pbi.14058>

16 Yamane, H., Ikeda, K., Hauck, N. R., Iezzoni, A. F. & Tao, R. Self-incompatibility (S) locus region of the mutated S6-haplotype of sour cherry (Prunus cerasus) contains a functional pollen S allele and a non-functional pistil S allele. *J Exp Bot* **54**, 2431-2437 (2003). <https://doi.org:10.1093/jxb/erg271>

17 Tao, R. *et al.* Molecular typing of S-alleles through identification, characterization and cDNA cloning for S-RNases in sweet cherry. *Journal of the American Society for Horticultural Science* **124**, 224-233 (1999). <https://doi.org:Doi> 10.21273/Jashs.124.3.224

18 Tao, R. *et al.* Self-compatible peach (Prunus persica) has mutant versions of the S haplotypes found in self-incompatible Prunus species. *Plant Mol Biol* **63**, 109-123 (2007). <https://doi.org:10.1007/s11103-006-9076-0>

19 Ushijima, K. *et al.* The S haplotype-specific F-box protein gene, SFB, is defective in self-compatible haplotypes of Prunus avium and P. mume. *Plant J* **39**, 573-586 (2004). <https://doi.org:10.1111/j.1365-313X.2004.02154.x>

20 Romero, C. *et al.* Analysis of the S-locus structure in Prunus armeniaca L. Identification of S-haplotype specific S-RNase and F-box genes. *Plant Mol Biol* **56**, 145-157 (2004). <https://doi.org:10.1007/s11103-004-2651-3>

21 Munoz-Sanz, J. V., Zuriaga, E., Lopez, I., Badenes, M. L. & Romero, C. Self-(in)compatibility in apricot germplasm is controlled by two major loci, S and M. *BMC Plant Biol* **17**, 82 (2017). <https://doi.org:10.1186/s12870-017-1027-1>

22 Mau, S. L., Raff, J. & Clarke, A. E. Isolation and partial characterization of components of Prunus avium L. styles, including an antigenic glycoprotein associated with a self-incompatibility genotype. *Planta* **156**, 505-516 (1982). <https://doi.org:10.1007/BF00392772>

23 Niska, R., Goldway, M. & Schneider, D. S6-RNase Is a Marker for Self-compatibility in Loquat (Eriobotrya japonica Lindl.). *Hortscience* **45**, 1146-1149 (2010). <https://doi.org:Doi> 10.21273/Hortsci.45.8.1146

24 Ishimizu, T., Shinkawa, T., Sakiyama, F. & Norioka, S. Primary structural features of rosaceous S-RNases associated with gametophytic self-incompatibility. *Plant Mol Biol* **37**, 931-941 (1998). <https://doi.org:10.1023/a:1006078500664>

25 Wang, S., Wang, Q., Zhang, Y., Qie, H. & Wang, H. Identification of two new S-RNases and molecular S-genotyping of twenty loquat cutivars [Eriobotrya japonica (Thunb.) Lindl.]. *Scientia Horticulturae* **218**, 48-55 (2017). <https://doi.org:10.1016/j.scienta.2017.02.002>

26 Wu, C., Zhang, Y., Wang, H., Yan, M. & Wang, S. Self-compatibility of ‘Zaohuang’ loquat is attributed to the lack of a 52 bp fragment in the S-RNase promoter. *Scientia Horticulturae* **301** (2022). <https://doi.org:10.1016/j.scienta.2022.111093>

27 Broothaerts, W., Janssens, G. A., Proost, P. & Broekaert, W. F. cDNA cloning and molecular analysis of two self-incompatibility alleles from apple. *Plant Mol Biol* **27**, 499-511 (1995). <https://doi.org:10.1007/BF00019317>

28 Zhu, S. *et al.* The Snapdragon Genomes Reveal the Evolutionary Dynamics of the S-Locus Supergene. *Mol Biol Evol* **40** (2023). <https://doi.org:10.1093/molbev/msad080>

29 Tang, D. *et al.* Genome evolution and diversity of wild and cultivated potatoes. *Nature* **606**, 535-541 (2022). <https://doi.org:10.1038/s41586-022-04822-x>

30 Clark, K. R. & Sims, T. L. The S-Ribonuclease Gene of Petunia-Hybrida Is Expressed in Nonstylar Tissue, Including Immature Anthers. *Plant Physiology* **106**, 25-36 (1994). <https://doi.org:DOI> 10.1104/pp.106.1.25

31 Clark, K. R., Okuley, J. J., Collins, P. D. & Sims, T. L. Sequence variability and developmental expression of S-alleles in self-incompatible and pseudo-self-compatible petunia. *Plant Cell* **2**, 815-826 (1990). <https://doi.org:10.1105/tpc.2.8.815>

32 Kondo, K. *et al.* Cultivated tomato has defects in both S-RNase and HT genes required for stylar function of self-incompatibility. *Plant Journal* **29**, 627-636 (2002). <https://doi.org:DOI> 10.1046/j.0960-7412.2001.01245.x

33 Hosaka, K. & Hanneman, R. E. Genetics of self-compatibility in a self-incompatible wild diploid potato species Solanum chacoense. 1. Detection of an S locus inhibitor (Sli) gene. *Euphytica* **99**, 191-197 (1998). <https://doi.org:Doi> 10.1023/A:1018353613431

34 Pham, G. M. *et al.* Construction of a chromosome-scale long-read reference genome assembly for potato. *Gigascience* **9** (2020). <https://doi.org:10.1093/gigascience/giaa100>

35 Ye, M. *et al.* Generation of self-compatible diploid potato by knockout of S-RNase. *Nat Plants* **4**, 651-654 (2018). <https://doi.org:10.1038/s41477-018-0218-6>

36 Hulse-Kemp, A. M. *et al.* Reference quality assembly of the 3.5-Gb genome of Capsicum annuum from a single linked-read library. *Hortic Res* **5**, 4 (2018). <https://doi.org:10.1038/s41438-017-0011-0>

37 Onus, A. N. & Pickersgill, B. Unilateral incompatibility in Capsicum (Solanaceae): occurrence and taxonomic distribution. *Ann Bot* **94**, 289-295 (2004). <https://doi.org:10.1093/aob/mch139>

38 McClure, B. A. *et al.* Style self-incompatibility gene products of Nicotiana alata are ribonucleases. *Nature* **342**, 955-957 (1989). <https://doi.org:10.1038/342955a0>

39 Honsho, C. *et al.* Association of T2/S-RNase With Self-Incompatibility of Japanese Citrus Accessions Examined by Transcriptomic, Phylogenetic, and Genetic Approaches. *Front Plant Sci* **12**, 638321 (2021). <https://doi.org:10.3389/fpls.2021.638321>
