# Supplementary figures and images for: Transposable elements cause the loss of self‐incompatibility in citrus
Source: Plant Biotechnol J. 2023 Dec 1;22(5):1113–31. doi: 10.1111/pbi.14250 (PMC11022811; doi:10.1111/pbi.14250)

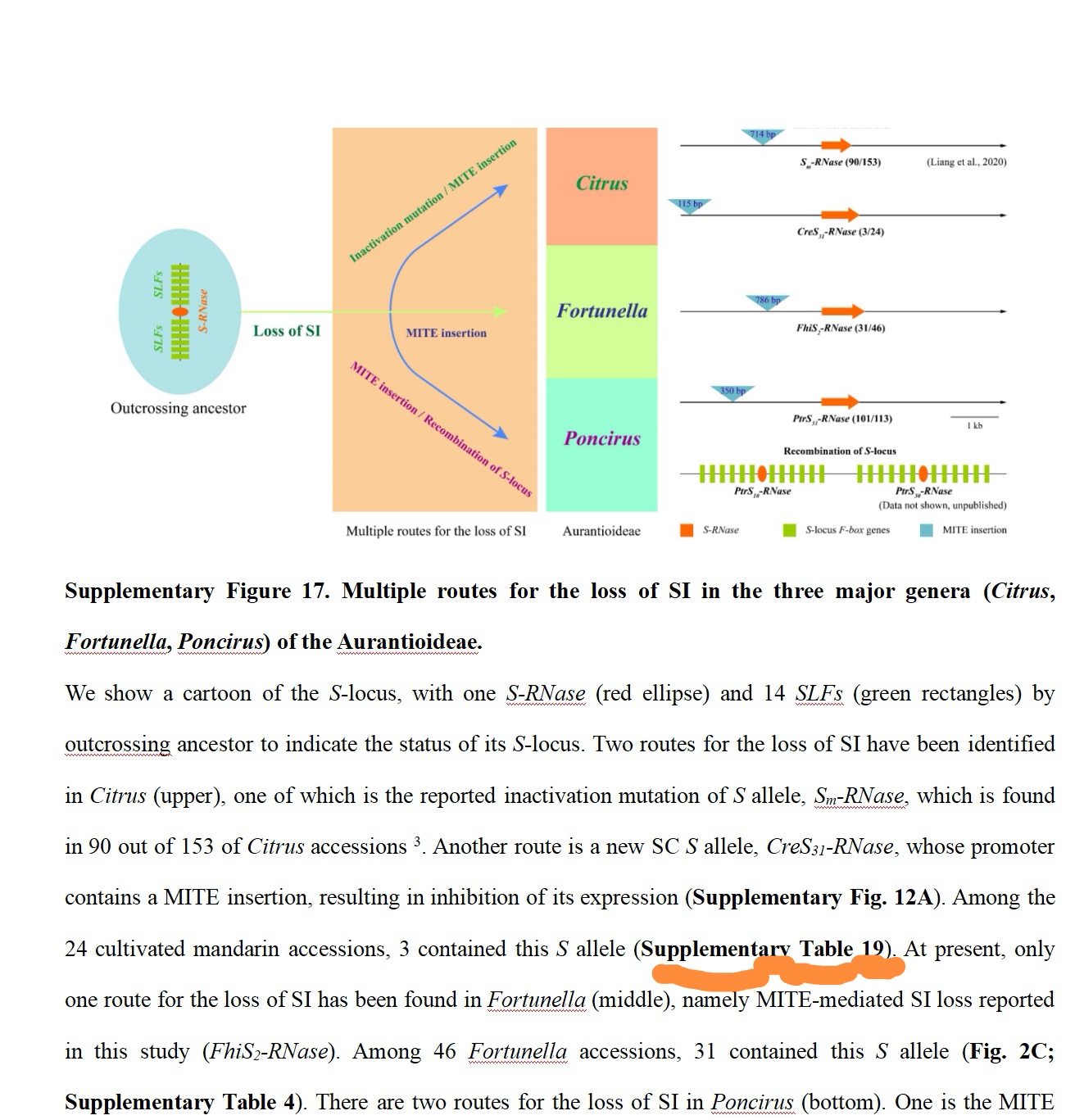

Supplement: Supplementary file 2 — Figure S17 Multiple routes for the loss of SI in the three major genera (Citrus, Fortunella, Poncirus) of the Aurantioideae. [file PBI-22-1113-s001.jpg]
